# Determination of ADP/ATP translocase isoform ratios in malignancy and cellular senescence

**DOI:** 10.1002/1878-0261.70039

**Published:** 2025-04-27

**Authors:** Zuzana Liblova, Dominika Maurencova, Barbora Salovska, Marek Kratky, Tomas Mracek, Zuzana Korandova, Alena Pecinova, Pavla Vasicova, David Rysanek, Ladislav Andera, Ivo Fabrik, Rudolf Kupcik, Pavel Kashmel, Pinky Sultana, Vojtech Tambor, Jiri Bartek, Josef Novak, Marie Vajrychova, Zdenek Hodny

**Affiliations:** ^1^ Laboratory of Genome Integrity Institute of Molecular Genetics of the Czech Academy of Sciences Prague Czech Republic; ^2^ Laboratory of Bioenergetics Institute of Physiology of the Czech Academy of Sciences Prague Czech Republic; ^3^ Biomedical Research Center University Hospital Hradec Kralove Czech Republic; ^4^ Danish Cancer Society Research Center Copenhagen Denmark; ^5^ Division of Genome Biology, Department of Medical Biochemistry and Biophysics, Science for Life Laboratory Karolinska Institute Stockholm Sweden; ^6^ Present address: Yale Cancer Biology Institute Yale University West Haven CT USA; ^7^ Present address: Department of Adipose Tissue Biology Institute of Physiology of the Czech Academy of Sciences Prague Czech Republic

**Keywords:** ADP/ATP translocase, cellular senescence, glioblastoma, targeted mass spectrometry

## Abstract

Cellular senescence has recently been recognized as a significant contributor to the poor prognosis of glioblastoma, one of the most aggressive brain tumors. Consequently, effectively eliminating senescent glioblastoma cells could benefit patients. Human ADP/ATP translocases (ANTs) play a role in oxidative phosphorylation in both normal and tumor cells. Previous research has shown that the sensitivity of senescent cells to mitochondria‐targeted senolytics depends on the level of ANT2. Here, we systematically mapped the transcript and protein levels of ANT isoforms in various types of senescence and glioblastoma tumorigenesis. We employed bioinformatics analysis, targeted mass spectrometry, RT‐PCR, immunoblotting, and assessment of cellular energy state to elucidate how individual ANT isoforms are expressed during the development of senescence in noncancerous and glioblastoma cells. We observed a consistent elevation of ANT1 protein levels across all tested senescence types, while ANT2 and ANT3 exhibited variable changes. Alterations in ANT protein isoform levels correlated with shifts in the cellular oxygen consumption rate. Our findings suggest that ANT isoforms are mutually interchangeable for oxidative phosphorylation and manipulating individual ANT isoforms could have potential for senolytic therapy.

## Graphical abstract

The individual functions of three isoforms exchanging ADP and ATP (ADP/ATP translocases; ANTs) on the mitochondrial membrane remain unclear. We developed a method for quantitatively differentiating highly similar human ANT1, ANT2, and ANT3 using parallel reaction monitoring. This method allowed us to assess changes in translocase levels during cellular senescence and correlate them with alterations in energy metabolism.

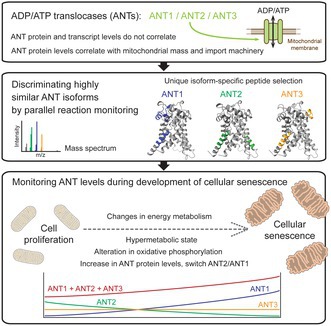

**Abbreviations**
AcN: acetonitrile
AJCC: American Joint Committee on Cancer
ANT: ADP/ATP translocase
ATCC: American Type Culture Collection
BSA: bovine serum albumin
DIS: drug‐induced senescence
DMEM: Dulbecco's modified Eagle medium
DMSO: dimethylsulfoxide
DOX: doxycycline
DTT: dithiothreitol
DTX: docetaxel
ECAR: extracellular acidification rate
ECOG: Eastern Cooperative Oncology Group Performance Status
EDTA: ethylenediaminetetraacetic acid
FA: formic acid
FC: fold change
FCCP: carbonyl cyanide 4‐(trifluoromethoxy)phenylhydrazone
FDR: false discovery rate
HCAECS: human coronary artery endothelial cells
IRIS: ionizing radiation‐induced cellular senescence
LC–MS: liquid chromatography coupled to mass spectrometry
NHBE: normal human bronchial epithelial cells
OCR: oxygen consumption rate
OXPHOS: mitochondrial oxidative phosphorylation
PBS: phosphate‐buffered saline
PBST: phosphate‐buffered saline with Tween
RPE‐1: telomerase‐immortalized human retinal pigment epithelial cell line
SASP: senescence‐associated secretory phenotype
SA‐β‐gal: senescence‐associated β‐galactosidase
SDC: sodium deoxycholate
SLC: solute carrier
TEAB: tetraethylammonium bromide
TFA: trifluoroacetic acid
TMZ: temozolomide
*μ*LC‐PRM: liquid chromatography coupled to parallel reaction monitoring

## 1 Introduction

Cellular senescence is a complex transformation of cellular phenotype primarily triggered by genotoxic stresses. These stresses can result from oncogene activation, redox disbalance, upregulation of specific signaling pathways, exposure to bacterial toxins, radiochemotherapy, and inflammation. As a primary response to unhealed DNA damage, cellular senescence acts as an anticancer barrier, preventing the proliferation of cells with damaged and unrepaired genomes. Under physiological conditions, senescent cells contribute to tissue regeneration and thrombocyte biogenesis and have also secretory function known as the senescence‐associated secretory phenotype (SASP; [1]). However, excessive accumulation of senescent cells in tissues can lead to mild but chronic inflammation and other complex effects associated with age‐related diseases, including cancer. One characteristic feature of senescent cells is alterations in their energetic metabolism. Consequently, targeting specific aspects of senescent cell metabolism hold promise for senolytic and senomorphic approaches [2, 3, 4].

Mitochondrial oxidative phosphorylation serves as a central cellular energy source, and its dysfunction has been linked to the early stage of cellular senescence [5, 6, 7]. During the evolution of eukaryotic cells, an essential event occurred–the adaptation of oxidative phosphorylation as a new energy source–facilitated by the symbiotic relationship between the bacterial precursor of the mitochondrion and the primordial eukaryotic cell. The proteins responsible for ADP/ATP exchange across the mitochondrial membrane are ADP/ATP translocases (ANTs), members of the solute carrier family (SLC). ANTs are the most abundant proteins in the inner mitochondrial membrane. In humans, four genes encode ANTs: ANT1 (*SLC25A4*), ANT2 (*SLC25A5*), ANT3 (*SLC25A6*), and ANT4 (*SLC25A31*). Although these genes are co‐expressed at the mRNA level in all tissues, their proportion vary depending on the tissue type [8, 9]. ANT1 mRNA is predominantly expressed in the heart and skeletal muscle, with lesser expression in the brain [10]. ANT2 transcript levels are generally low, except in the liver and proliferating cells *in vitro* [11, 12, 13]. ANT3 is a housekeeping gene ubiquitously expressed in all tissues [13]. The last discovered family member, ANT4, is expressed in the liver and brain, but its predominant expression occurs in the testis [14].

Although the primary structure of ANT proteins, except for ANT4 [14], is highly similar, functional differences have been reported as recently reviewed [15, 16]. Notably, in the context of aging, previous studies demonstrated that ANT2 transcription is suppressed during the transition of proliferative cells to a senescent state *in vitro* and during the development of cellular senescence in cancer cells [17]. Additionally, downregulation of ANT2 transcripts was observed in dermal fibroblasts from human aging skin, and ectopic expression of ANT2 reversed senescence in replicative senescent dermal fibroblasts [18]. Conversely, restoring ANT2 expression in both proliferative and senescent cells conferred resistance to the anticancer agent MitoTam (tamoxifen targeted to mitochondria through triphenyl phosphonium moiety; [19]). On the other side, siRNA knockdown of ANT1 induced senescence in lung epithelial cells [20].

To clearly dissect the differences in physiological function for individual ANT isoforms, it is necessary to have selective, sensitive, and quantitative methods for their evaluation at the protein level. ANT1, ANT2, and ANT3 share approximately 90% of their amino acid sequence [14], making it challenging to prepare isoform‐specific antibodies. Although isoform‐specific antibodies against ANT1 and ANT2 (but not ANT3) are commercially available, immunoblotting, as a semiquantitative technique, is limited by experimental variability due to uneven sample loading, protein transfer, and signal background. Moreover, varying antibody affinities hinder the straightforward determination of ANT stoichiometric ratios. While full scan bottom‐up MS techniques are well‐established for exploring thousands of proteins in biological systems [21], mapping protein isoforms remains challenging even with modern next‐generation mass spectrometry [22].

In this study, we conducted a bioinformatic analysis of publicly available proteome and transcriptome datasets from human glioma and lung carcinoma tumor sample. Our findings revealed that ANT1, ANT2, and ANT3 protein levels do not correlate with their transcript levels. To quantify ANT isoform ratios at the protein level, we developed a targeted mass spectrometry‐based quantitative method for monitoring isoform‐specific ANT1, ANT2, and ANT3 peptides. We quantified changes in ANT isoforms in selected cellular senescence models at both the protein and transcript levels. By this approach, we observed an increase in ANT1 protein levels in response to various senescence inducers across all tested senescence types. At the same time, ANT2 and ANT3 exhibited variable changes depending on the specific senescence model. Furthermore, we noted an association between increased oxidative phosphorylation and ANT protein levels in senescent cells, suggesting tight regulation of ANT isoform protein levels linked to mitochondrial biogenesis.

## 2 Experimental procedures

### 2.1 Cell culture

Unless otherwise indicated in the text, chemicals and reagents were purchased from Sigma?Aldrich (St. Louis, MO, USA) in the highest available purity grade. Telomerase‐immortalized human retinal pigment epithelial cell line hTERT RPE‐1 (RPE‐1), human foreskin fibroblasts (BJ), and human glioma A‐172 and U‐87 MG cell lines were purchased from the American Type Culture Collection (ATCC, Manassas, VA, USA, cat. no. CLR‐4000, CRL‐2522, CRL‐1620, HTB‐14, respectively). A‐172, U‐87 MG, BJ, and parental RPE‐1 cells were authenticated less than year ago by the GENERI BIOTECH (Hradec Kralove, Czech Republic).

RPE‐1 cells, A‐172, and U‐87 MG cell lines were cultured in Dulbecco's modified Eagle medium with 4.5 g·L^−1^ glucose (DMEM, Gibco, Thermo Fisher Scientific, Waltham, MA, USA) supplemented with 10% fetal bovine serum (FBS), penicillin (100 U·mL^−1^), and streptomycin (100 μg·mL^−1^).

BJ cells were cultured in Dulbecco's modified Eagle medium with 1 g·L^−1^ glucose (DMEM, Gibco, Thermo Fisher Scientific) supplemented with 10% FBS, penicillin (100 U·mL^−1^), and streptomycin (100 μg·mL^−1^).

For the specific long‐term (32 days) experiment (Fig. 3), RPE‐1 cells were cultured in Dulbecco's modified Eagle medium with 4.5 g·L^−1^ glucose (DMEM, Gibco, Thermo Fisher Scientific) supplemented with 200 mg·L^−1^ l‐proline, 10% fetal bovine serum (FBS), penicillin (100 U·mL^−1^), and streptomycin (100 μg·mL^−1^).

All cells were maintained at 37 °C under a 5% CO_2_ atmosphere and 95% humidity and tested regularly for mycoplasma infection.

### 2.2 Preparation of cell lines with ectopic expression of cyclin‐dependent kinase inhibitors

RPE‐1 cells with tetracycline‐inducible expression of p16INK4A (RPE‐1 p16) and p21WAF1 (RPE‐1 p21) were prepared by transducing cells with recombinant lentiviruses LentiX‐Tet‐ONE puro (San Jose, CA, USA, Takara Bio USA, Inc.) containing cDNA encoding p16INK4A or p21WAF1. Puromycin (2 μg·mL^−1^ final concentration) was used to select positive cells. Control RPE‐1 tet‐ONE empty cells contained an empty vector only.

### 2.3 Induction of cellular senescence and contact inhibition

To prepare ionizing radiation‐induced senescent cells (IRIS), RPE‐1 cells were seeded at a density of 2 × 10^4^ cells·cm^−2^ [23]. The next day, cells were exposed to a single dose of 20 Gy (150 kV, 10 mA) using Pantak HF160 (Gulmay, Surrey, UK) X‐ray instrument equipped with Pantak Seifert HF320 generator, MXR‐161 X‐ray tube (Comet, AG, Flamatt, Switzerland), and aluminum filter using current 1–10 mA [23]. The cells were harvested in 2, 4, 8, 16, and 32 days in three biological replicates. Nonirradiated proliferating RPE‐1 cells served as control.

Docetaxel (DTX; Thermo Scientific, Bremen, Germany, ACRO456262500) drug‐induced senescent BJ cells (BJ DTX) were prepared according to the protocol of Sapega et al. [24] with minor modifications. Briefly, BJ cells were exposed to 5 nm DTX followed by medium exchange every third day for 8 days.

To prepare temozolomide‐(TMZ; Selleckchem, Houston, TX, USA, S1237) induced glioblastoma senescent cells, A‐172 and U‐87 MG were seeded at a density of 15 000 cells·cm^−2^ and, after 24 h, exposed to TMZ at a final concentration of 100 μm (applied with every medium exchange) for 14 days.

To induce cellular senescence by ectopic expression of cyclin‐dependent kinase inhibitors p21 [25] and p16 [26], p21‐RPE‐1 and p16‐RPE‐1 cells were exposed to 1 μg·mL^−1^ doxycycline (DOX) to induce the transgene expression for 7 days (applied three times with exchange of fresh media).

To induce quiescence by contact inhibition, RPE‐1 cells were seeded at 15 000 cells·cm^−2^ density and cultured for 10 days to reach full confluence. Quiescence was determined by the absence of cell proliferation.

### 2.4 Senescence‐associated beta‐galactosidase assay

The senescence‐associated beta‐galactosidase activity of control and senescent cells was performed according to the modified protocol of Dimri et al. [27]. Briefly, cells grown on glass coverslips were fixed with 0.5% glutaraldehyde at room temperature for 15 min, washed with PBS supplemented with 1 mm MgCl_2_, and then incubated with prewarmed X‐gal solution (1 mg·mL^−1^ X‐gal (Sigma‐Aldrich), 0.12 mm K_3_Fe[CN]_6_, 0.12 mm K_4_Fe[CN]_6_ × 3 H_2_O, 1 mm MgCl_2_ in PBS, pH 6.0) at 37 °C and 5% CO_2_ for 1–3 h (or until blue color in senescent cells was visible). After the development of visible blue coloring inside the cells, coverslips were mounted in Mowiol containing 4',6‐diamidino‐2‐phenylindole (DAPI; Sigma‐Aldrich) and viewed by fluorescence microscope Leica DM6000 (Leica Microsystems, Wetzlar, Germany) equipped with a color camera and objective HC PLAN APO 20×/0.70 DRY PH2.

### 2.5 DNA replication

DNA replication was estimated using the EdU DNA incorporation assay (Invitrogen Click‐iT EdU, Waltham, MA, USA, Thermo Fisher Scientific) according to the manufacturer's protocol. Briefly, control and irradiated (day 14) RPE‐1 cells were exposed to a 24‐h‐long pulse of EdU before harvest. Cell nuclei were counterstained by 4',6‐diamidino‐2‐phenylindole (DAPI; Sigma‐Aldrich).

### 2.6 SDS/PAGE and immunoblotting

Immunoblotting experiments were performed in biological triplicates. Cells were washed three times with 1× PBS, harvested into 95 °C‐heated SDS sample lysis buffer (2% SDS, 50 mm Tris – Cl, pH 6.8, 10% glycerol in double distilled H_2_O), and sonicated on Diagenode Bioruptor 300 (Diagenode, Liege, Belgium, 4 °C, 5 cycles, high intensity, 30 s on/off). The concentration of proteins was estimated by the Pierce BCA Protein Assay (Thermo Fisher Scientific, Waltham). DTT (100 mm) and 0.01% bromophenol blue were added to the lysates before separation by SDS/PAGE (10% gels were used). Equal protein amounts were loaded into each well. Proteins were electrotransferred onto a nitrocellulose membrane (Amersham™ Protran™ 0.45 μm NC, GE Healthcare, Chalfont St Giles, UK) using semi‐dry transfer and detected by specific primary antibodies (at 4 °C overnight) ANT1/SLC25A4 (Cell Signaling Technology, Danvers, MA, USA; #51755S, E3E9Y, dilution 1 : 1000) or ANT2/SLC25A5 (Cell Signaling Technology; #14671S, E2B9D, dilution 1 : 1000) in a blocking solution (5% nonfat dry milk in PBST). Alpha‐tubulin (GenScript, Piscataway, NJ, USA; A01410, dilution 1 : 1000) or actin (Sigma, St. Louis, MO, USA; A2066, dilution 1 : 10 000) were used as loading controls. Membranes were incubated with secondary antibody (RT/1 h) IgG‐HRP (Bio‐Rad, Hercules, CA, USA, dilution 1 : 10 000) anti‐Rabbit (#170‐6515) or anti‐Mouse (#170‐6516) diluted in blocking buffer. Peroxidase activity was detected by using the Fusion Solo S imaging system (Vilber, France, Collégien).

### 2.7 Evaluation of the specificity of ANT antibodies by RNA interference

To assess the specificity of anti‐ANT1 and anti‐ANT2 antibodies for immunoblotting analyses, the knockdown of ANT1 and ANT2 by RNA interference was performed using A‐172 and HeLa cells with high ANT1 and ANT2 expression, respectively. The following siRNAs were used: siANT1 #2 [28] 5′‐TTG TAT TGG TGT TGT ATG AdTdT‐3′, and siANT2 #2 (Austin, TX, USA, Ambion; s1375) 5′‐GAA GAU UGC UCG UGA UGA AdTdT‐3′, to knock down ANT1 and ANT2, respectively. siRNAs were manufactured by Merck (Sigma‐Aldrich). siNC (MISSION® siRNA Universal Negative Control #1 #SIC001) was used as a reference. Cells were seeded 24 h before transfection at a density of 16 000 and 28 000 cells·cm^−2^ for HeLa and A‐172, respectively. Transfection of siRNAs was performed with Lipofectamine™ RNAiMAX (Invitrogen) according to the manufacturer's protocol, followed by incubation for 48 h.

### 2.8 Real‐time quantitative reverse transcription PCR

Cells were lysed using RLT lysis buffer (RNeasy Mini Kit Qiagen, Germantown, MD, USA) with DTT (20 μL of 2 m DTT/1 mL RLT). Total RNA was isolated according to the manufacturer's protocol. The amount of RNA was determined spectrophotometrically by NanoDrop 2000 (Thermo Fisher Scientific, Carlsbad, CA, USA) and reverse‐transcribed into cDNA with random hexamer primers and MultiScribe reverse transcriptase (Applied Biosystems, Foster City, CA, USA). Expression levels of ANT1, ANT2, and ANT3 were quantified using qPCR relative quantification (Applied Biosystems SYBR™ Select Master Mix, Applied Biosystems 7300 Real‐Time PCR System with SDS software, Thermo Fisher Scientific). The relative quantity of cDNA was determined by the ΔΔ*C*_t_ method [29]; data were normalized to β‐actin (ACTB). Samples were measured in triplicates. Primers used are listed in Table S3.

### 2.9 Transcriptome analysis

Transcriptomic data of senescent cells, brain tumors, pigment naevi, adenomas, and liver lesions were downloaded from Gene Expression Omnibus ([30]) and BioStudies [31] ArrayExpress databases. Bioconductor [32] package was utilized for loading and manipulating microarray data in r statistical software [33]. The RMA (Robust Multiarray Averaging) method for normalization was applied to all datasets. The limma bioconductor package [34] was used to perform differential expression analysis and statistical tests. In these datasets, we were only interested in changes in ANTs, thus *P*‐values were used to determine if these changes were significant. The statistical significance threshold was set to 0.01. Outliers were identified using PCA (Principal Component Analysis) and hierarchical clustering methods. Points that occurred too far from the other data from the same group were declared as outliers and removed. Several outliers were found in the data: 3 in dataset E‐GEOD‐50161 and 1 in dataset E‐GEOD‐4183. In dataset GSE4290, 7 outliers and 4 samples of patients without a precise diagnosis were found and removed. Dataset E‐MTAB‐949 included 5 samples of patients with unique diagnoses that could not be incorporated into any investigated groups; thus, they were removed. Several groups of samples unsuitable for our analysis were also removed. In the case of dataset E‐GEOD‐13712, only young and senescent cells under static conditions were used for our analysis. Similarly, only young and senescent cells from datasets E‐GEOD‐19864 and E‐MEXP‐2683 were used. Only untreated senescent and young cells were used from the E‐GEOD‐77239 dataset. The GSE13276 dataset contains samples from the normal brain, tumor, and tissues surrounding the tumor. Only tumor and healthy brain samples were used.

In total, senescent datasets contain two (E‐MEXP‐2683, E‐MEXP‐2283, E‐GEOD‐11954, E‐GEOD‐19864), three (E‐GEOD‐77239, E‐GEOD‐13712, GSE100014), four (E‐GEOD‐16058), and five (GSE35957) experimental replicates. The brain tumor datasets contain 8 (GSE13276), 127 (GSE4290), and 169 (E‐GEOD‐50161) samples. The pigment naevi, adenomas, liver lesions datasets contain 6 (E‐GEOD‐2487), 11 (E‐GEOD‐53223), 12 (E‐GEOD‐15960), 17 (GSE46517), 23 (E‐GEOD‐4183), and 275 (E‐MTAB‐950) samples.

Publicly available single‐cell transcriptomics data of glioblastoma tumor cells [35] extracted from 11 tumors were downloaded, analyzed using python 3.11.4 [36], and visualized using the seaborn 0.12.0 [37].

### 2.10 mRNA and protein correlation

python 3.11.4 [36] was used for transcriptome and proteome correlation analysis. Two different datasets containing samples from different tumors were downloaded. The first dataset [38] contains 99 samples of glioblastoma patients. To compare correlations between mRNA expression and protein amount, we focused on 19 938 protein‐coding mRNAs and a global proteome containing 10 977 quantified proteins. In total, 10 965 genes had both a transcriptome and a proteome. We used gene‐level read count, Fragments Per Kilobase of transcript per Million mapped reads (FPKM) for mRNA and normalized zero‐centered values of intensity for proteins. Only 10 941 genes were used for calculating correlations between protein intensity and mRNA since 24 genes had an mRNA level equal to 0 FPKM. The dataset also contains 10 normal brain samples used to compare changes between normal brain and glioblastoma. The second dataset [39] contains 108 lung squamous cell carcinoma samples. Again, we focused on 19 938 protein‐coding mRNAs and a global proteome containing 11 485 quantified proteins. In total, 11 469 genes had both mRNA and protein. Again, we used FPKM for mRNA and two‐component normalized proteome data, as described in the article. However, only 11 442 genes were used for calculating correlations between protein intensity and mRNA because 27 genes had an mRNA level equal to 0 FPKM. Pearson and Spearman correlation coefficients were calculated for each gene in each dataset and their *P*‐values. The matplotlib 3.7.2 [40] and the seaborn 0.12.0 [37] were used for visualizations. A database of 1136 human mitochondrial genes was downloaded from MitoCarta3.0 [41]. In the glioblastoma dataset, 991 mitochondrial genes were present. However, correlations were calculated for only 988 genes because 3 had an mRNA level equal to 0 FPKM. There were 985 mitochondrial genes in the lung cancer dataset, and correlations were calculated for only 982 genes because 3 had null mRNA levels.

### 2.11 Cellular respiration (OCR and ECAR)

Analysis of glycolysis and mitochondrial oxidative phosphorylation (OXPHOS) via quantification of the extracellular acidification rate (ECAR) and oxygen consumption rate (OCR) were processed according to Fitch et al. [42] using Seahorse Analyzers XFe24 (Agilent Technologies, Inc., Santa Clara, CA, USA). To induce glycolysis levels, glucose was used as an ECAR level enhancer. One day before measurement, cells were passaged using trypsin/EDTA (trypsin Biowest, Nuaillé, France, P5957; EDTA, Sigma‐Aldrich, 6381‐92‐6) 0.025% solution, DMEM (Thermo Scientific, Bremen, with GlutaMAX instead of glutamine) was added to neutralize trypsin, and the uniform cell suspension was created by pipetting. Cells were counted using Bürker chamber and seeded in growth media in total volume 250 μL, 15 000 cells (U‐87 MG and A‐172), 5000 cells (RPE‐1 for IRIS, p16, p21) or 2800 cells (for BJ and all controls) into each well of Seahorse XF24 V7 PS Cell Culture Microplates (24‐well), four wells were without cells (250 μL of media only) for background correction. Plates were placed back into the incubator (5% CO_2_, 37 °C, humidified) for 24 h. RPE‐1 cells for contact inhibition were seeded directly into Seahorse XF24 V7 PS Cell Culture Microplates (5000 cells per well) and cultivated for 10 days. On the day of the assay, cells were washed with 1 mL of freshly prepared assay media [DMEM without phenol red, with 0.2% bovine serum albumin (BSA), pH 7.4, Sigma‐Aldrich, A9647] prewarmed to 37 °C followed with media replacement with final 500 μL of assay media. Then, cell culture plates were placed in a humidified 37 °C incubator without CO_2_ for 30 min before measurement. In the meantime, 10× drug solutions were prepared by diluting the drug stocks in dimethylsulfoxide (DMSO) and loaded in a drug port for each well of the sensor cartridge. The final concentrations of drugs in wells were as follows: Port B oligomycin 1 μm (Sigma‐Aldrich, O4876); Port C carbonyl cyanide 4‐(trifluoromethoxy)phenylhydrazone (FCCP) 6 μm (Sigma‐Aldrich, C2920); Port D rotenone 1 μm (Sigma‐Aldrich, R8875)/antimycin A (1 μm·mL^−1^; Sigma‐Aldrich, A8674) in 50 mm 2‐deoxy‐D‐glucose (Sigma‐Aldrich, D8375) with 0.2% BSA (Sigma‐Aldrich, A7030) and Hoechst 33342 (Thermo Scientific, Bremen, 62 249). To induce glycolysis and enhance ECAR levels, Port A was loaded with glucose (Sigma‐Aldrich, G7528) in the final concentration of 10 mm as a substrate. After 30 min of incubation at 37 °C without CO_2_, the plate was ready for measuring. Data were normalized by counting nuclear‐stained cells in each well of the plate using the BioTek Instruments' Cytation 3. To compute OCR and ECAR characteristics, two measurements were performed before each injection of drugs and two at the end of the experiment. The following OCR characteristics were computed: baseline OCR as time points 1–5, ATP‐linked OCR as time points 1–3, maximal respiration OCR as 4–5, spare capacity OCR as 4–2, and proton leak as 3–5. Note that the presence of glucose lowered measured OCR, and therefore, the basal OCR is computed in the absence of added glucose, and maximal OCR is computed in the presence of added glucose. For ECAR, the following characteristics were computed: baseline ECAR as 1–5, glycolic capacity ECAR as 3–5, and glycolytic reserve ECAR as 3–2. The OCR/ECAR ratio was computed as an ATP‐linked OCR divided by glycolytic ECAR. Statistical significance was computed using the Mann–Whitney–Wilcoxon test, and the significance level was set at 0.05.

### 2.12 Targeted ADP/ATP translocase quantification by parallel reaction monitoring

#### 2.12.1 Cellular protein extraction

To extract cellular proteins, cells were washed once with PBS, trypsinized, neutralized by growth medium, transferred to an Eppendorf tube, centrifuged (500 ***g***, 5 min at RT), 2× washed in PBS, and stored at −80 °C. Cells were then harvested with lysis buffer (1% SDC/50 mm Tris‐Cl) and sonicated using a Bioruptor (4 °C, 3–15 cycles, high intensity, 30 s on/off). The remaining cell debris was removed by centrifugation (4 °C, 3000 ***g***, 10 min), and protein concentration was measured by the BCA assay.

#### 2.12.2 Liquid chromatography coupledto parallel reaction monitoring

LC–MS solvents were purchased from Honeywell (Morris Plains, NJ, USA). Synthetic isotopically labeled ANT peptides with ‘heavy’ lysine (^13^C_6_^15^N_2_) or arginine (^13^C_6_^15^N_4_) (JPT Peptide Tech, Berlin, Germany) were redissolved using 100 mm triethylammonium bicarbonate buffer, pH 8.5 (TEAB, Thermo Scientific, Rockford, IL, USA) to get a concentration of 2.5 pmol·μL^−1^ and added to RPE‐1 cell lysate before protein digestion. Heavy peptides were added to 6 μg of total protein as follows: 50 fmol of each ANT1 peptide, 100 fmol of each ANT2 peptide, and 100 fmol of each ANT3 peptide. Enzymatic digestion of proteins was processed in 1% sodium deoxycholate (SDC)/100 mm TEAB. Reversibly oxidized cysteines were reduced with 5 mm tris(2‐carboxyethyl) phosphine hydrochloride at 60 °C for 60 min. Free thiol groups in reduced cysteines were further blocked using 10 mm methyl methanethiosulfonate at room temperature for 30 min. Proteins were digested using rLys‐C (Wako, Pure Chemical Industries, Osaka, Japan) at 37 °C for 3 h, followed by sequencing grade trypsin (Promega, Madison, WI, USA) digestion at 37 °C at a 1 : 50 ratio (enzyme/substrate) overnight. Digestion was stopped by the addition of trifluoroacetic acid (TFA) to a final concentration of 1% (v/v), and precipitated SDC was removed by extraction in water‐saturated ethyl acetate. Samples were desalted on Empore C18‐SD SPE cartridges (3M, St. Paul, MN, USA), previously conditioned by methanol, and equilibrated using 0.1% TFA in 5% AcN. Peptides were eluted by 0.05% TFA in 50% ACN and evaporated to dryness.

Digested protein samples were diluted in 0.1% formic acid (FA), 3% dimethylsulfoxide (DMSO), and 0.4% acetic acid (HAc) to a concentration of 1 μg·μL^−1^ and injected to an UltiMate 3000 binary RSLC System (Thermo Scientific, Bremen) in three technical replicates. The analytical system consisted of a Halo Peptide ES‐C18, 2.7 μm, 160 Å, 1.0 mm × 250 mm analytical column (Advanced Materials Technology, Wilmington, DE, USA). Tryptic peptides were separated with a linear gradient from 0.2% to 45% of 0.1% formic acid, 78% acetonitrile (AcN), 0.4% HAc, and 3% DMSO at a flow rate of 68 μL·min^−1^ at 55 °C for 20 min. Peptides were sprayed to a hybrid quadrupole‐Orbitrap mass spectrometer Q Exactive Plus using a HESI‐II probe (Thermo Scientific, Bremen) at 3.0 kV. Positive ion MS spectra were acquired for selected *m*/*z* within a defined 3‐min window with a 1 × 10^6^ AGC target at 35 000 resolution and a maximum ion time of 110 ms. An isolation window of 2.0 *m*/*z* and a normalized collision energy of 28% were used.

#### 2.12.3 PRM data processing

Survey PRM records were processed in Proteome Discoverer v. 3.0 (PD) to design the PD output .msf and .pdResult files used to build MS/MS library in Skyline [43]. The target false discovery (FDR) rate was set to 0.01 for highly confident peptide hits calculated by the Target Decoy PSM Validator. Monoisotopic precursor (2^+^), y, and b ions (1^+^) were monitored. Product ions were selected from *m*/*z* > precursors to 5 ions. Peptide settings were as follows: trypsin [KR|P] as protease, length of peptide sequence 6–25 amino acids, peptide matching based on library and filter, heavy arginine (^13^C_6_^15^N_4_) and lysine (^13^C_6_^15^N_2_) as isotope modifications, thiomethylation of cysteine residues and methionine oxidation as structural modifications. Transitions of target ANT peptides and heavy labeled standards were extracted, and total peak areas of target peptides were normalized to heavy peptide equivalents as a ratio target‐to‐heavy. Each ratio was expressed as a mean from three technical replicates. The level of each ANT isoform was defined based on the target‐to‐heavy ratio of selected peptides in three biological replicates.

#### 2.12.4 Selection of ANT peptides for parallel reaction monitoring

Due to the restricted tissue expression of ANT4, only ANT1, ANT2, and ANT3 were selected for identification in RPE‐1 cells by μLC‐PRM analysis. Owing to a high degree of ANT1, ANT2, and ANT3 sequence similarity, unique areas were firstly predicted based on multiple sequence alignment conducted in clustal omega, as shown in Fig. S3A [44]. From the identified peptide candidates, we excluded three N‐terminal signal peptides (Table S2) prone to acetylation (Fig. S3A); see below for details. During data evaluation of the ionizing radiation‐induced senescence (IRIS) dynamic experiment, we additionally excluded one ANT2‐unique peptide (Table S2) due to significant artificial methionine oxidation of the standard peptide (Fig. S3B) and one ANT3‐unique peptide (Table S2) because of the insufficient signal of transitions (Fig. S3C) and RT shift (Fig. S3D). Peptides with missed cleavage are typically excluded from target mass analysis. However, considering the lack of peptides unique for ANT3, we included the miss‐cleaved ANT3‐unique peptide IFRDEGGK. Despite one miss cleavage (IF **R** DEGGK), in the case of this peptide, we found consistent fragmentation (Fig. S3E), the stable signal between injections (Fig. S3F), reproducible retention time, and the efficient number of transitions in the case of both the target and the standard peptide (Fig. S3G). In conclusion, we quantified ANTs based on three peptides unique to ANT1 and two unique to ANT2 and ANT3 during IRIS development in RPE‐1 (Table S1).

#### 2.12.5 *μ*LC‐PRM optimization

In the initial PRM analysis, we searched for optimal injection of RPE‐1 peptides to achieve signal stability in the 5 min‐acquisition time windows. In the initial experiment, we injected 2, 4, and 6 μg of complex sample spiked with an equal amount of heavy ANT standards (200 fmol). We observed the trend of increasing signal of both target and standard ANT peptides (Fig. S3I) and lower variability of peak area toward the higher injection loading (Fig. S3J). Injection of 6 μg was therefore used in further experiments as making a compromise between efficient ion signal and reasonable consumption of samples. The default value of normalized collision energy (NCE; 28%) was the most appropriate for the targeted monitoring of ANTs (Fig. S3K). Subsequent experiments assessed the proper concentration of ANT‐labeled ANT standards to be added to RPE‐1 lysate. We designed the concentration range from 10 to 1000 fmol of each standard spiked into 6 μg of RPE‐1 lysate. To avoid overloading of ANT‐labeled peptide standards compared to ANT targets, we aimed to use a concentration of ANT‐labeled peptides that did not exceed fivefold of ANT target level. This corresponded with labeled peptide concentrations of 50 fmol for ANT1 (Fig. S3L) and 100 fmol for ANT2 and ANT3 (Fig. S3M,N), respectively. Considering a low number of target peptides, we reduced the length of LC‐PRM from 78 to 35 min to save instrument time, and we also narrowed the 5 min‐acquisition windows to 3 min. *μ*LC‐PRM with a duration of 35 min showed slightly lower peak area variability but comparable peak areas of heavy ANT standards (Fig. S3O,P) even after using lower resolution and reduced time of ion filling (35 000 and 110 ms; Fig. S3Q).

### 2.13 2D LC–MS/MS analysis of U87 senescent proteome after temozolomide treatment

#### 2.13.1 Sample preparation

Cell pellets (see Section 2.12.1) were lysed in lysis buffer 3% SDC in 200 mm TEAB. Total protein concentration was determined in all samples using a micro BCA protein assay (Thermo Fisher Sci, Rockford, IL, USA). Enzymatic digestion of proteins (50 μg) was processed in 100 mm TEAB. Disulfide bonds were reduced with 5 mm tris(2‐carboxyethyl) phosphine hydrochloride at 37 °C for 30 min, and the free thiol groups were blocked using 10 mm methyl methanethiosulfonate at room temperature for 10 min. To remove contaminant reagents, 6 volumes of (~ 300 μL) of prechilled acetone were added, and protein precipitation was allowed to proceed at −20 °C overnight (O/N). Samples were centrifuged at 12 000 ***g*** at 4 °C for 10 min, and acetone was decanted without disturbing the pellet. The pellet was allowed to dry for 2 min to remove the acetone remnants and redissolved in 50 μL of 100 mm TEAB. Finally, proteins were digested by a mixture of lysyl endopeptidase and trypsin (Promega) at a 1 : 25 enzyme‐to‐protein ratio (w/w) at 37 °C overnight. After digestion, total peptide concentration was determined using Fluorometric Peptide Assay (Thermo Fisher Scientific, Rockford, IL, USA) according to the manufacturer's instruction. Tandem Mass Tag (TMT) 10‐plex (Thermo Scientific, Rockford) isobaric label reagents were dissolved in 20 μL of AcN. Isobaric labeling of peptides was allowed to proceed at room temperature for 60 min, and the reaction was terminated by 5% hydroxylamine according to the manufacturer's instruction. An equal amount of labeled peptides (30 μg) was mixed to TMT multiplex, and remnants of reagents were removed by solid phase extraction on Peptide Desalting Spin Columns (Thermo Fisher Scientific, Rockford) packed by C18 reversed phase according to the manufacturer's instruction, and prepared TMT multiplexes were evaporated to dryness.

#### 2.13.2 High‐pH prefractionation

TMT multiplex was redissolved in 2% AcN/10 mm NH_4_FA. Peptides were separated using XBridge BEH column C18, 2.5 μm, 2.1 μm × 150 mm (Waters, Milford, MA, USA) in a linear gradient of mobile phase B (80% AcN/10 mm NH_4_FA) at a flow rate of 0.3 mL·min^−1^. The gradient was running from 0% B to 2% B in 2 min, followed by 2% B to 20% B in 9 min, from 20% B to 50% B in 41 min, and from 50% B to 52% B in 5.5 min. In total, the gradient time was 57.5 min. Fractions were collected into a 96‐well polypropylene plate (Agilent Technologies) at 45 s from intervals 3.7 to 57.5 min, yielding 72 fractions in a volume of 225 μL. Collected fractions were subsequently concatenated into 24 fractions and evaporated to dryness.

#### 2.13.3 Liquid chromatography coupled to tandem mass spectrometry (nanoLC–MS/MS)

Collected fractions were redissolved in 0.1% trifluoroacetic acid (TFA), 2% acetonitrile (AcN) and injected on an UltiMate 3000 RSLCnano System (Thermo Scientific, Bremen) in two technical replicates. The analytical system consisted of a PepMap100 C18, 3 μm, 100 Å, 75 μm × 20 mm trap column and a PepMap RSLC C18, 2 μm, 100 Å, 75 μm × 250 mm analytical column (both from Thermo Scientific, Bremen). The samples were loaded onto the trap column at a flow rate 5 μL·min^−1^ of 0.1% TFA, 2% AcN for 5 min. Tryptic peptides were separated from mobile phase A (2% ACN/0.1% FA) and B (80% ACN/0.1% FA), running from 2% B to 34.5% B in 70 min, followed by 34.5% B to 45% B in 10 min at a flow rate of 250 nL·min^−1^. Eluted peptides were sprayed into Exploris 480 using a NanoSpray Flex (NG) ion source (Thermo Scientific, Bremen) at 1.8 kV spray voltage for 107 min. Positive ion full scan MS spectra were acquired in the range of *m*/*z* 350–1400, at 60 000 resolution (at *m*/*z* 200), 300% AGC target value, and maximum IT of 25 ms. FAIMS Pro Duo interface operated under compensation voltage values of −45 and −60 V and at a gas flow rate of 4.6 L·min^−1^. The fragmentation (MS/MS) spectra were acquired for the 10 most intense precursors in TurboTMT mode. An isolation window of 1.3 *m*/*z (*precursor fit 70%) and a normalized collision energy of 35% were used. Each fragmentation spectrum was acquired at a resolution of 15 000 (at *m*/*z* 200), with a normalized AGC target of 200% and a maximum IT of 34 ms. The first mass was fixed to 110 *m*/*z*. Charge states < 2 and > 5 were excluded, and dynamic exclusion was set to 17 s after fragmentation *n* = 1 times.

#### 2.13.4 Data acquisition and evaluation

Recorded MS and MS/MS spectra were processed and searched in Proteome Discoverer 3.0 against a reviewed UniProt human reference protein database. Trypsin digestion specificity with up to two missed cleavages was used. Cysteine thiomethylation was set as a fixed modification, and oxidation of methionine and proline was selected as a variable modification. The mass tolerance in MS and MS/MS mode was left at the default value for the initial search, and 6 ppm was set as mass tolerance in MS mode for the main search. The false discovery rate for protein identification was left at the default value. Output files from Proteome Discoverer were processed in Perseus and R statistical environment v. 4.4.1 [33]. Reporter ion intensities were log_2_‐transformed, and differential expression LIMMA analysis [34] was performed in an R statistical environment with Benjamini–Hochberg false discovery rate (FDR) correction at the significance level of 0.05. All quantified proteins were classified according to the significant difference between the control and TMZ‐treated group (B‐H FDR *P*‐value <0.05), and down/upregulation based on log_2_FC value.

### 2.14 Statistical analyses

The scipy python library 1.11.2 [45] was used for statistics. The statistical significance of qPCR data was calculated for log_2_(FC), which has a normal distribution; thus, the *t*‐test was used. The Mann–Whitney–Wilcoxon test was used to compute statistical significances of cellular respiration OCR and ECAR characteristics. Statistical significance for Pearson and Spearman correlations between mRNA and proteins was calculated using the ‘pearsonr’ and ‘spearmanr’ functions from the scipy 1.11.2 library [45]. The adjusted *P*‐values were calculated using the Benjamini–Hochberg procedure. The Mann–Whitney–Wilcoxon test was used to calculate the significance of changes in ANT protein levels in patient groups defined by glioblastoma size, as the distribution in individual groups is not normal. The Mann–Whitney–Wilcoxon test was used to compare the distributions of mRNA‐protein correlation coefficients for all proteins and mitochondrial proteins. The significance level was set at 0.05 for the statistical tests mentioned above. The limma bioconductor r package was used for statistical analysis of microarray data [34], and the significance level was set to 0.01. The ANT levels of treated telomerase‐immortalized human retinal pigment epithelial cells (RPE‐1; IRIS) were compared to proliferative control by two‐way ANOVA on significance level 0.05, followed by Dunn's test for multiple comparisons. The graphpad prism 6 was used for ANOVA and for the construction of bar plots. Scatter plots were designed using the r package ‘ggplot2’ [46]. Spearman's correlation coefficients and *P*‐value were used to characterize the correlation of levels of ANT protein with the respective transcripts, which were determined using the R default corr.test() [33]. For the analysis of differentially expressed genes in U‐87 MG cells after TMZ, the significance level was set to 0.05 and the threshold for log_2_FC was set to 0.5. The magnitude of *P*‐values is indicated using asterisk (*0.01 ≤ *P* ≤ 0.05; **0.001 ≤ *P* ≤ 0.01; ***0.0001 ≤ *P* ≤ 0.001; ****P ≤ 0.0001). *P*‐values higher than 5e‐02 indicate nonsignificant change; thus, they are marked with ‘ns’.

## 3 Results

### 3.1 Changes of ANTs transcript levels in various *in vitro* models of cellular senescence and premalignant and malignant lesions

**Fig. 1:**
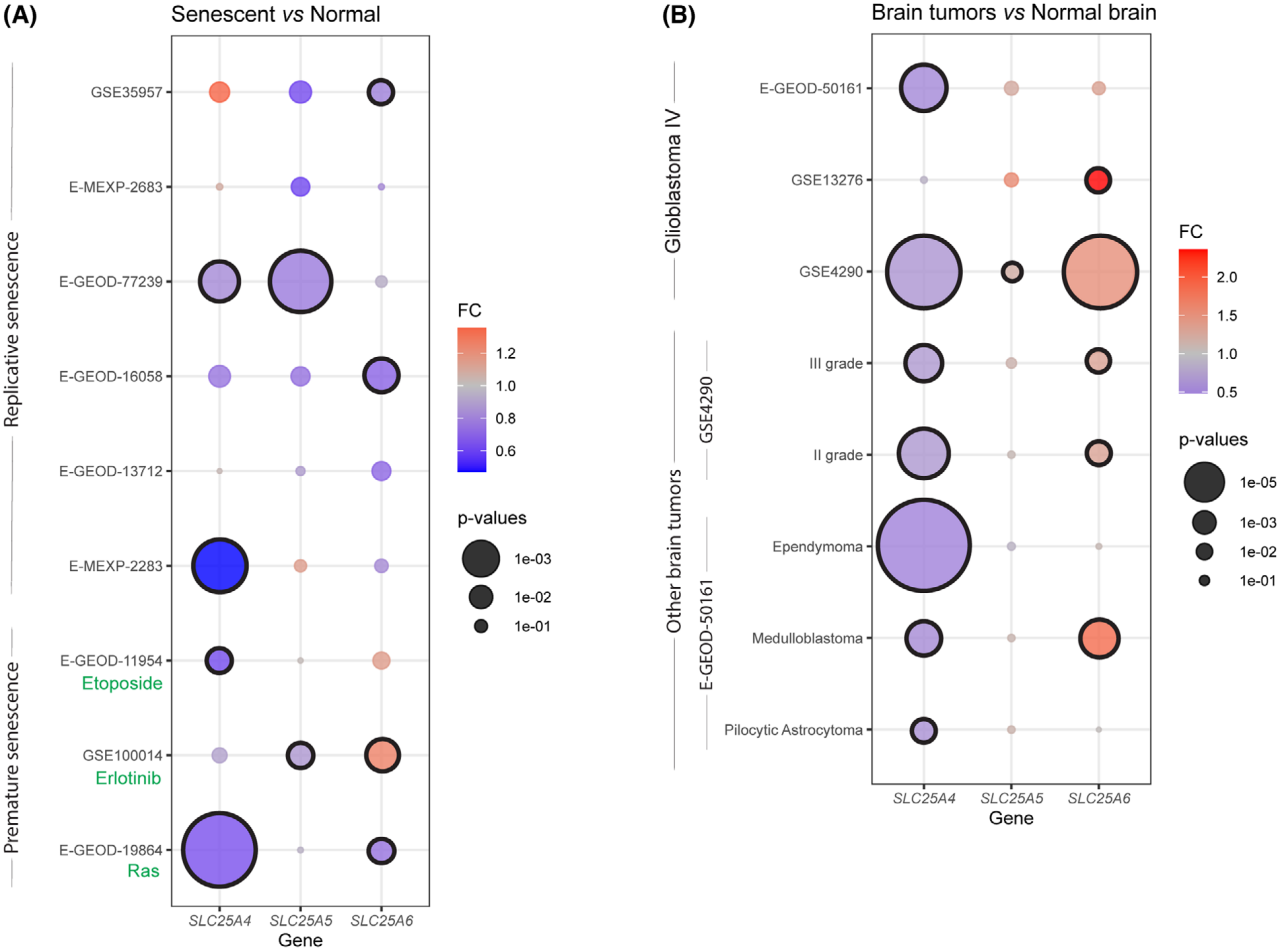
Changes in ANT transcripts in publicly available expression profiling datasets. Changes in ANT transcripts in control and senescent cells (A) and in normal brain and brain tumors (B) are shown with their statistical significance level (*P*‐value) represented by the area of the individual dots and color coded by fold change (FC) value. Significant changes (*P*‐value < 0.05) are marked with a black circle. (A, B) Empirical Bayes moderated *t*‐statistics were used for statistics. Senescent datasets contain 2 (E‐MEXP‐2683, E‐MEXP‐2283, E‐GEOD‐11954, E‐GEOD‐19864), 3 (E‐GEOD‐77239, E‐GEOD‐13712, GSE100014), 4 (E‐GEOD‐16058), and 5 (GSE35957) experimental replicates. The brain tumor datasets contain 8 (GSE13276), 127 (GSE4290), and 169 (E‐GEOD‐50161) samples.

Given that ANT2 expression is actively repressed during the transition from the growth phase to cellular senescence *in vitro* by components of the TGF‐beta signaling pathway [11, 17, 19, 47], we aimed to verify whether this decrease in ANT2 transcript levels is a general phenomenon associated with *in vitro* senescence and whether it also occurs under different conditions associated with senescence *in vivo*. To this end, we analyzed publicly available expression profiling datasets of various types of senescent cells (Table 1) to compare changes in ANT1, ANT2, ANT3, and ANT4 transcripts during the establishment of cellular senescence *in vitro* (Fig. 1A), in various premalignant and malignant lesions (Fig. S1A), and in several types of human brain tumors (Fig. 1B). These analyses suggest that the expression levels of ANT1 (*SLC25A4*), ANT2 (*SLC25A5*), and ANT3 (*SLC25A6*) isoforms change under different conditions, but no consistent pattern corresponding to the *in vitro* findings can be discerned from the data. Note that ANT4 mRNA levels were generally low and not affected during the establishment of senescence.

**Table 1:** List of datasets analyzed for ANT isoform transcript levels in cellular senescence *in vitro* and in human benign and malignant tumors.

ID	Cell type	References	LINK
E‐GEOD‐13712	HUVEC replicative senescence	[81]	https://www.ebi.ac.uk/biostudies/arrayexpress/studies/E‐GEOD‐13712
E‐GEOD‐19864	IMR‐90 Ras‐induced senescence	[82]	https://www.ebi.ac.uk/biostudies/arrayexpress/studies/E‐GEOD‐19864
GSE100014	NHBE erlotinib‐induced senescence	[83]	https://www.ncbi.nlm.nih.gov/geo/query/acc.cgi?acc=GSE100014
E‐GEOD‐16058	HMEC replicative senescence	[84]	https://www.ebi.ac.uk/biostudies/arrayexpress/studies/E‐GEOD‐16058
E‐GEOD‐77239	HCAECS replicative senescence	[85]	https://www.ebi.ac.uk/biostudies/arrayexpress/studies/E‐GEOD‐77239
E‐GEOD‐11954	HSC etoposide‐induced senescence	[86]	https://www.ebi.ac.uk/biostudies/arrayexpress/studies/E‐GEOD‐11954
GSE35957	hMSC replicative senescence	[87]	https://www.ncbi.nlm.nih.gov/geo/query/acc.cgi?acc=GSE35957
E‐MEXP‐2283	HUVEC replicative senescence	–	https://www.ebi.ac.uk/biostudies/arrayexpress/studies/E‐MEXP‐2283
E‐MEXP‐2683	PTEC replicative senescence	–	https://www.ebi.ac.uk/biostudies/arrayexpress/studies/E‐MEXP‐2683
E‐GEOD‐50161	Glioblastoma	[88]	https://www.ebi.ac.uk/biostudies/arrayexpress/studies/E‐GEOD‐50161
GSE13276	Glioblastoma	[89]	https://www.ncbi.nlm.nih.gov/geo/query/acc.cgi?acc=GSE13276
GSE4290	Glioblastoma	[90]	https://www.ncbi.nlm.nih.gov/geo/query/acc.cgi?acc=GSE4290
E‐GEOD‐4183	Adenoma	[91]	https://www.ebi.ac.uk/biostudies/arrayexpress/studies/E‐GEOD‐4183
E‐GEOD‐15960	Adenoma	[92]	https://www.ebi.ac.uk/biostudies/arrayexpress/studies/E‐GEOD‐15960
GSE46517	Nevus	[93]	https://www.ncbi.nlm.nih.gov/geo/query/acc.cgi?acc=GSE46517
E‐GEOD‐53223	Nevus	–	https://www.ebi.ac.uk/biostudies/arrayexpress/studies/E‐GEOD‐53223
E‐MTAB‐950	Virus‐induced benign tumors	[94]	https://www.ebi.ac.uk/biostudies/arrayexpress/studies/E‐MTAB‐950
E‐GEOD‐2487	ITV, ITM, ITM‐E6E7 induced and bypassed senescence	[95]	https://www.ebi.ac.uk/biostudies/arrayexpress/studies/E‐GEOD‐2487?query=E‐GEOD‐2487

With the availability of single‐cell sequencing data from glioblastoma patients [35], we analyzed the expression of individual ANT isoforms in tumor subpopulations. As shown in Fig. S1B,C, the expression of ANT isoform transcripts within the tumor is highly heterogeneous. A certain proportion of tumor‐derived cells expressed all three ANT isoforms (Dividing B cells, OPC, Dividing OPC, Immature Astrocyte, Mature IPC/Newborn Neuron; 22.9% cells). Notably, there were specific subpopulations that expressed dominantly a single isoform (Neuron, Dividing Neuron, CGE iN; ANT1 2.4%; ANT2 6.5%; ANT3 16.5%). Additionally, we investigated whether ANT2 expression overlaps with KI67 (MKI67), a proliferation marker. Although some subpopulations expressed both transcripts (12.4% cells), some ANT2‐positive cells were KI67‐negative (Fig. S1D), suggesting that ANT2 expression may not always correlate with cell proliferation status.

### 3.2 Correlation of ANT isoform transcript and protein levels in glioblastoma and lung cancer

**Fig. 2:**
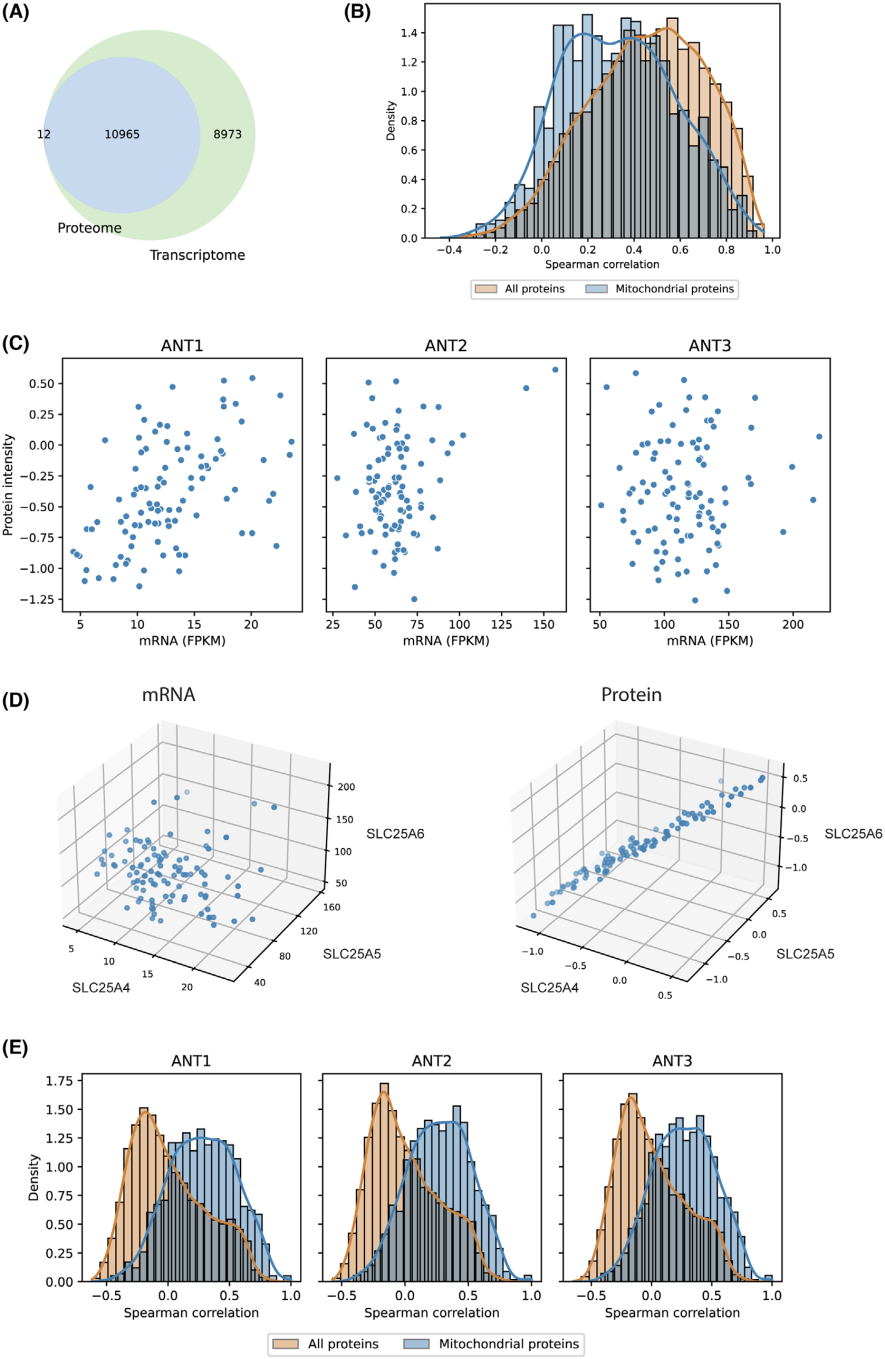
Protein and mRNA correlation in glioblastoma. (A) Venn diagram for genes recorded in the transcriptome and proteome. (B) Distribution of mRNA‐protein correlations for all genes and mitochondrial proteins. Mann–Whitney–Wilcoxon test was used for statistics. (C) Scatter plots for ANT with transcript level (mRNA) on the *x*‐axis and protein intensity on the *y*‐axis. (D) 3D scatter plots for ANT transcript level and ANT protein intensity. (E) Comparison of distributions of ANT protein correlations with all proteins and with mitochondrial proteins.

The previous findings led us to question whether ANT transcript and protein levels are correlated. To clarify this, we utilized a previously published omics analysis dataset of 99 glioblastoma patients containing both transcriptome and proteome data [38]. We identified 10 965 proteins with their mRNA levels (Fig. 2A). First, we performed a general correlation of the identified proteins with their mRNA levels. Median Spearman correlation is 0.47 (Fig. 2B and for examples of mRNA/protein correlation modes, see Fig. S2A). Mitochondrial proteins (from MitoCarta3.0 database [41]) have a significantly lower Spearman correlation between transcript and protein levels compared to all proteins (*P* = 2.88e‐56, Fig. 2B). The median correlation for mitochondrial proteins is 0.33. ANTs belong to mitochondrial proteins, and just like the other mitochondrial proteins, they have a poor correlation between transcript and protein levels in glioblastoma (Spearman correlation: ANT1 0.5; ANT2 0.04; ANT3 0.01; note ANT4 was not detected at protein level; Fig. 2C and Fig. S2B). Surprisingly, all three ANT proteins, but not their mRNA levels, were strongly intercorrelated across all 99 samples, indicating synchronicity in maintaining their protein level (Fig. 2D and Fig. S2C). These data suggested that the ANT protein levels follow mitochondrial mass. Indeed, correlations of ANT proteins with mitochondrial proteins are stronger compared to correlations with all proteins (Fig. 2E). The most correlated proteins with ANTs are other members of the SLC group (SLC25A3, SLC30A9) and import receptor of the outer mitochondrial membrane TOMM70. Furthermore, the lists of the strongest correlations with ANT1–3 differ slightly. However, all three ANT proteins are also strongly correlated with most other mitochondria internal and external membrane proteins (TIMM50, TIMM29, TIMM22, TIMM21, TOMM22, etc.), mitochondria‐encoded subunits of oxidative phosphorylation complexes (MT‐ND1, MT‐ND2, MT‐ND4, MT‐ND5, MT‐ND6, MT‐CYB, MT‐CO1, MT‐CO2, MT‐CO3, MT‐ATP6, and MT‐ATP8), and proteins that regulate mitochondrial dynamics (such as GDAP1, OPA1, IMMT, SNPH, MFN2, DNM1L, and MFN1; Fig. S2D).

Next, we investigated whether this is a general phenomenon or specificity of glioblastoma using a previously published omics analysis dataset of 108 patients with squamous cell lung carcinoma [39]. Overall, ANT correlations are similar to glioblastoma. There is no close correlation between transcript and protein levels for ANT isoforms (Spearman correlation: ANT1 0.17; ANT2 0.25; ANT3 0.26; note ANT4 was not detected at protein level; Fig. S2E,F), all three ANT proteins were strongly intercorrelated across all 108 samples (Fig. S2G,H), and ANT proteins are correlated with mitochondrial mass (Fig. S2I,J). Furthermore, we asked whether ANT transcript and protein levels were associated with the clinicopathological state of glioblastoma patients (Fig. S2K). ANT2 and ANT3 transcript levels mildly positively correlated with the time from diagnosis to death. All three ANTs weakly positively correlated with tumor size at the protein level, indicating a supportive role of energetic metabolism on tumor growth. Note that the American Joint Committee on Cancer (AJCC) Cancer Staging System was used to define the tumor size categories in the datasets. Glioblastomas in the T4 category (containing tumors greater than 7 cm) have a significantly higher level of ANT protein intensity than other categories (Fig. S2L). For lung carcinoma, there are not enough data points for a statistical test in the T4 category (only 2 cases), but the trend indicates a similar dependence as for glioblastoma (Fig. S2M). In conclusion, these data indicate no close correlation between transcript and protein levels for ANT isoforms in glioblastoma and in lung carcinoma. In both datasets, all three ANT proteins were strongly intercorrelated and correlated with mitochondrial mass, such as the proteins that regulate mitochondrial dynamics and mitochondrial import machinery.

### 3.3 Development of the mass spectrometry determination of ANT relative protein levels revealed a switch between ANT1 and ANT2 isoforms during the development of radiation‐induced senescence

Based on the noncorrelation between ANT transcript and protein levels and the lack of antibodies specific to all three ANT1–3 isoforms, we aimed to develop a fast and reliable approach to determine the relative levels of individual ANT protein isoforms in cell protein lysates. For this purpose, we employed a mass spectrometry method based on microbore liquid chromatography coupled with parallel reaction monitoring (*μ*LC‐PRM). Due to the restricted tissue expression of ANT4, only ANT1, ANT2, and ANT3 were selected for identification by *μ*LC–MS/MS analysis. Briefly, we selected three peptides unique to ANT1 and two unique to ANT2 or ANT3 for specific detection of ANT proteins (Fig. S3A and Table S1). We performed a basic analysis for rational peptide selection to exclude peptides inappropriate from the analytical point of view (Fig. S3B–H). We optimized *μ*LC–MS/MS analysis of RPE‐1 cells and proved that ANT1, ANT2, and ANT3 can be distinguished based on these unique peptides even in the background of more than 1700 proteins identified in 6 μg of RPE‐1 lysate (Fig. S3I–K). Finally, the concentration of ANT heavy labeled peptides was defined as an optimization of the *μ*LC‐PRM method (Fig. S3L–Q).

**Fig. 3:**
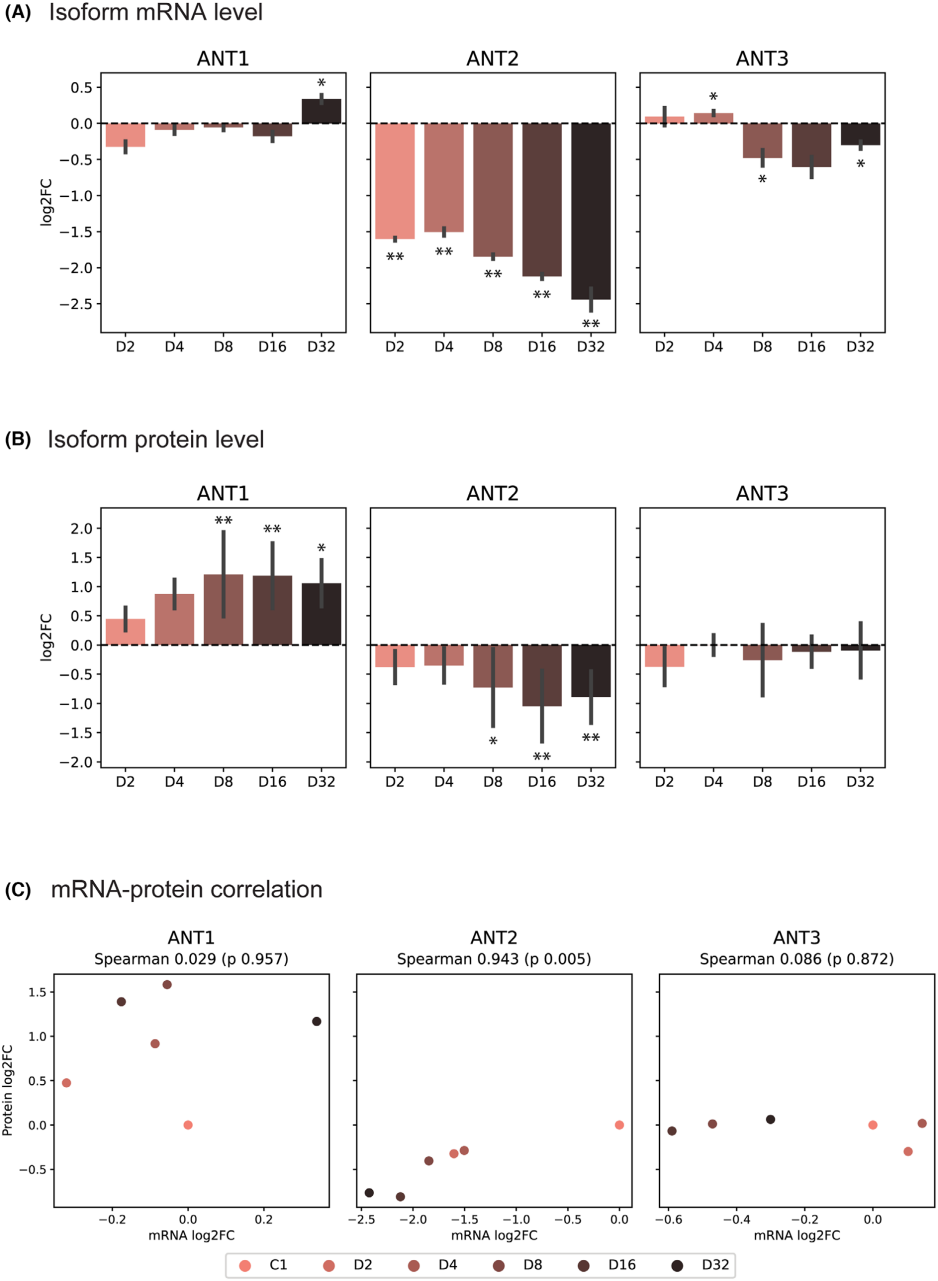
Partial switch of ANT2 with ANT1 protein levels during IRIS development. (A) The ANT mRNA detected by qPCR and (B) protein levels detected by *μ*LC‐PRM were determined in IRIS RPE‐1 cells up to 32 days after γ‐radiation (D2–D32) and compared to control (C1; proliferating) RPE‐1. Each ANT mRNA or protein is expressed as a mean of log_2_ fold change between the treated and proliferating RPE‐1 calculated from three biological replicates. The standard errors are depicted as error bars. The significant protein changes during the development of IRIS are indicated by asterisks (*P ≤ 0.05, **P ≤ 0.01). (C) Correlation of log_2_ fold change of ANT mRNA and protein levels. Spearman's correlation coefficient (*Spearman*) and *P*‐value are shown. (A) One sampled *t*‐test, (B) two‐way ANOVA followed by Dunn's test for multiple comparisons, and (C) Spearman's rank correlation coefficient were used for statistics.

To validate *μ*LC‐PRM usability, we followed dynamic changes in ANT isoform protein levels using an established model of ionizing radiation‐induced senescence (IRIS) in human telomerase‐immortalized RPE‐1 cells. For this purpose, RPE‐1 cells were exposed to 20 Gy to induce IRIS, as previously described [25, 48]. After IR, control and irradiated RPE‐1 cells were harvested on days 2, 4, 8, 16, and 32, and the ANT transcript and protein levels were determined using RT‐PCR and *μ*LC‐PRM, respectively. The development of the senescent phenotype was confirmed by the determination of elevated senescence‐associated β‐galactosidase (SA‐β‐gal) activity and lack of cell proliferation in the EdU incorporation assay at day 14 (Fig. S3R,S). As shown in Fig. 3A, ANT2 mRNA levels decreased significantly during IRIS development in accordance with previous examinations of ANT2 mRNA in response to the induction of quiescence and oxidative stress‐induced senescence [11, 17]. In contrast, ANT1 mRNA changed significantly only in the last observed time point, 32 days after IR. ANT3 mRNA levels did not show an unequivocal trend, as we observed significant elevation (D4) but also a decline (D8 and D32) compared to proliferating controls.

*μ*LC‐PRM analysis of ANT isoform peptides (Fig. 3B and Fig. S3T) showed that the ANT2 signal continuously decreased during the IRIS development, with the highest decline observed 32 days after IR. On the contrary, the ANT1 protein level was significantly upregulated on day 8 after IR compared to proliferating RPE‐1 cells. No significant changes in ANT3 protein levels were observed during the IRIS development up to 32 days in conformity with ANT3 transcript levels.

During IRIS development, the correlation between ANT transcript and protein levels demonstrated low concordance between mRNA and protein levels for ANT1 and ANT3. In contrast, ANT2 exhibited a significant downregulation of both mRNA and protein levels on the 16th and 32nd days of IRIS development. Thus, consistent with our analyses of patients' data, changes in protein levels did not correlate with changes in transcript levels (Fig. 3C).

In conclusion, *μ*LC‐PRM proved to be a suitable method to monitor changes in ANT protein isoforms. With its help, we found that IRIS is accompanied by a decrease in ANT2 but an increase in ANT1 protein levels, which in the case of ANT1 was not followed by an increase in the level of ANT1 mRNA.

### 3.4 Radiation‐induced senescent RPE‐1 cells show the hypermetabolic phenotype

**Fig. 4:**
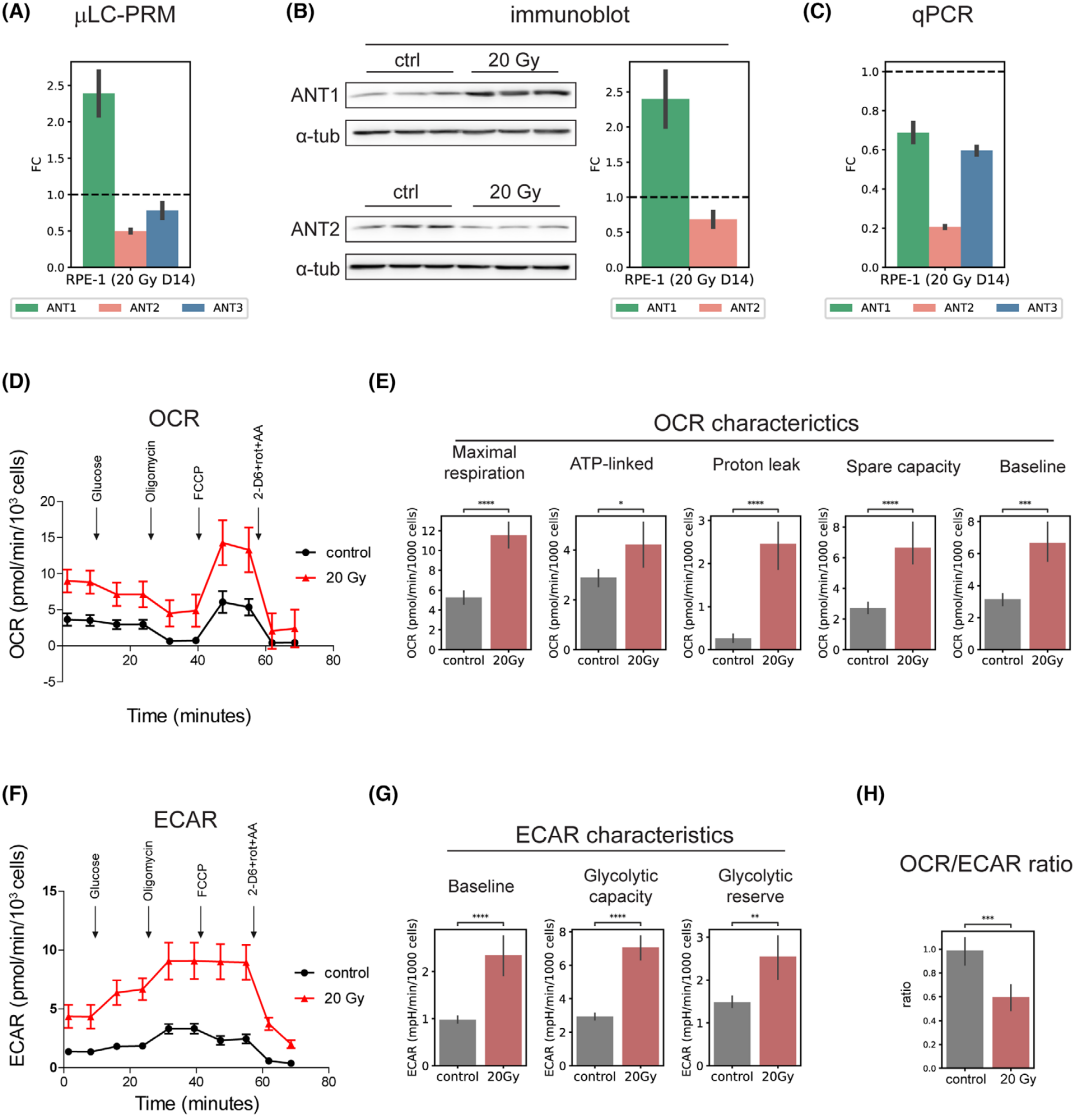
Metabolic parameters of IRIS RPE‐1 cells. The ANT1, ANT2, and ANT3 (A) protein levels detected by *μ*LC‐PRM, (B) protein levels detected by immunoblotting, with α‐tubulin serving as a loading control, and (C) transcript levels normalized to β‐actin detected by qPCR were determined for IRIS RPE‐1 cells 14 days (D14) after γ‐radiation (20 Gy) and for the proliferating control. The mean fold change between the treatment and proliferating RPE‐1, calculated from three experimental replicates, is shown. The standard errors are depicted as error bars. Upon the same experimental condition, analysis of glycolysis and OXPHOS using a Seahorse analyzer in IRIS RPE‐1 cells (20 Gy; D14; three biological replicates) was done. (D) the oxygen consumption rate (OCR) and its derived characteristics (E) together with the extracellular acidification rate (ECAR) (F) and its characteristics (G) are shown. (H) OCR/ECAR ratio indicates dependence on glycolysis or oxidative phosphorylation. (D–H) The mean obtained from at least three experimental replicates with the standard errors are shown. (E, G, H) Mann–Whitney–Wilcoxon test was used for statistics.

We further asked whether changes in the protein levels of ANT isoforms during IRIS would be reflected in a change in energy metabolism. Thus, we exposed the cells to a single 20 Gy dose of IR and let the IRIS develop for 14 days. We verified changes in ANT1 protein elevation, ANT2 protein decrease, and a slight change in ANT3 by the *μ*LC‐PRM (Fig. 4A). In this case, we also determined the protein levels of ANT1 and ANT2 using immunoblotting using commercially available antibodies against ANT1 and ANT2 and obtained the same results as with the *μ*LC‐PRM (Fig. 4B; the specificity of ANT1 and ANT2 antibodies was validated by RNA interference, see Fig. S4A–C). As in the previous IRIS experiment, we obtained the same results when measuring the transcript levels of ANT isoforms (Fig. 4C). The development of the IRIS phenotype was confirmed by the determination of elevated SA‐β‐gal activity (Fig. S4D).

Concurrently, changes in cellular metabolic phenotype, as represented by oxygen consumption rate (OCR) and extracellular acidification rate (ECAR), were determined in proliferating cells and their senescent counterparts using the Seahorse real‐time metabolic analyzer. Measurement of OCR (Fig. 4D,E) showed a significant increase in baseline and maximal respiration in IRIS cells. Interestingly, senescent cells exhibited an increased proton leak, which may indicate mitochondrial damage. Measurement of ECAR (Fig. 4F,G) showed increased basal glycolysis and glycolytic capacity following senescence induction. Overall, we observed an increase in OCR and an even more substantial increase in ECAR, indicating a shift toward glycolysis in the IRIS model (Fig. 4H).

In conclusion, OCR and ECAR measurements show the hypermetabolic phenotype of radiation‐induced senescent cells.

### 3.5 The increase of ANT1 protein level is a general feature of senescence development

To confirm whether changes in ANT levels are a general feature of senescent cells, we analyzed several additional types of senescence in normal, nontransformed, and cancerous cells. Specifically, we examined: (a) normal human skin fibroblasts BJ induced to drug‐induced senescence (DIS) by docetaxel (DTX); (b) a senescent‐like phenotype in RPE‐1 cells induced by doxycycline (DOX)‐regulated ectopic expression of CDK protein inhibitors p21^waf1^ (p21) or p16^INK4A^ (p16; [25, 26]); and (c) a senescent‐like phenotype in two glioblastoma cell lines, U‐87 MG and A‐172, induced by the anticancer drug temozolomide (TMZ). The establishment of senescence was validated by the SA‐β‐Gal assay (Fig. S5A–E). Additionally, cellular senescence in RPE‐1 cells was compared to quiescence induced by contact inhibition.

**Fig. 5:**
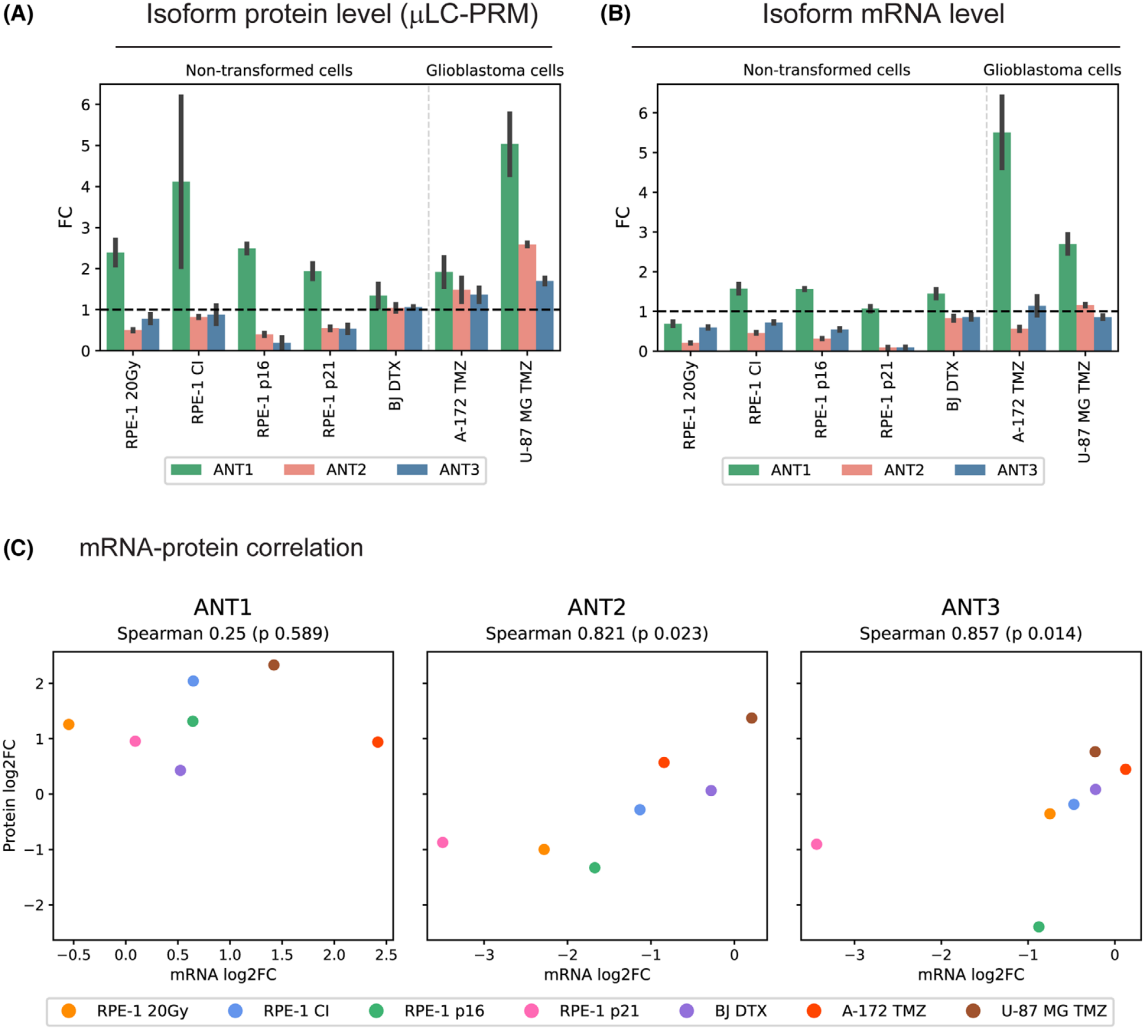
Changes in ANT1, ANT2, and ANT3 protein and transcript levels in various types of cellular senescence. The ANT1, ANT2, and ANT3 (A) protein levels detected by *μ*LC‐PRM and (B) transcript levels normalized to β‐actin transcript level detected by qPCR were determined in different senescent models and corresponding proliferating control. The mean of fold change between the treatment and proliferating control, calculated from three experimental replicates, is shown. (A, B) The data of IRIS RPE‐1 cells (20 Gy; D14) from Fig. 4A,C are shown again for easier comparison of all senescence models. Error bars represent standard errors. (C) The correlation of log_2_ fold change of ANT mRNA and protein levels was calculated. Spearman's correlation coefficient *(Spearman)* and *P*‐value are shown. The tested models include contact inhibition (RPE‐1 CI), IRIS (RPE‐1 20 Gy), p16 and p21 CDK‐inhibitor overexpression (RPE‐1 p16 and RPE‐1 p21) in RPE‐1 cells, docetaxel‐induced senescence in normal BJ fibroblasts (BJ DTX), and TMZ‐induced senescence in glioblastoma cell lines (A‐172 TMZ and U‐87 MG TMZ), calculated from three experimental replicates. Spearman's rank correlation coefficient was used for statistics.

In all senescent and quiescent cell populations, *μ*LC‐PRM analysis revealed an elevation of ANT1 protein levels related to untreated cells. However, ANT2 and ANT3 exhibited different changes depending on the cell type and senescence inducer (Fig. 5A). Note that the already presented analysis of IRIS RPE‐1 cells (20 Gy; D14) is shown again for an easier comparison of all senescent models. Immunoblotting using commercially available antibodies confirmed changes in ANT1 and ANT2 protein levels (Fig. S5F). Notably, ANT transcripts determined by RT‐qPCR (Fig. 5B) again did not closely mirror the levels of ANT protein isoforms across all models. Correlation analysis between protein levels measured by *μ*LC‐PRM with mRNA determined by RT‐qPCR showed a weak nonsignificant correlation for ANT1 (Spearman 0.250, *P* = 0.589) and a robust significant correlation for ANT2 (Spearman 0.821, *P* = 0.023) and ANT3 (Spearman 0.857, *P* = 0.014; Fig. 5C).

In conclusion, *μ*LC‐PRM analysis revealed an increase in the level of ANT1 protein during the establishment of cellular senescence in all tested senescence types. At the same time, ANT2 and ANT3 exhibited variable changes depending on the type of senescence. The increase in ANT1 protein levels was not followed closely by the increase in ANT1 transcript levels.

### 3.6 The increase in the metabolic activity of senescent cells correlates with an increase in total ANT protein levels

As we showed that the IRIS RPE‐1 cells revealed a hypermetabolic phenotype, next, we determined changes in cellular metabolic phenotype (OCR and ECAR) for proliferating cells and their growth‐inhibited counterparts across all tested models (see Fig. S6A,B for OCR and ECAR profiles, respectively, and Fig. S6C,D for derived metabolic characteristics). Similar to IRIS RPE‐1, A‐172 TMZ, and U‐87 TMZ senescent cells exhibited increased OCR characteristics as baseline, ATP‐linked respiration, maximal respiration, spare capacity, and increased proton leak compared to proliferation control, whereas RPE‐1 p21 and BJ DTX exhibited a significant increase in some of the followed OCR characteristics. Notably, RPE‐1 p16 cells showed a significant decrease of ATP‐linked respiration, maximal respiration, and spare capacity OCR characteristics. Additionally, all senescence models (A‐172 TMZ, U‐87 TMZ, BJ DTX, and RPE‐1 p21) except RPE‐1 p16 exhibited significantly increased glycolytic capacity, similar as was observed for IRIS RPE‐1. The changes in other ECAR characteristics were variable. The analysis of quiescent RPE‐1 cells showed the increase in maximal respiration, proton leak, and spare capacity compared to proliferation control, a similar trend as observed in IRIS RPE‐1. Conversely, quiescent RPE‐1 cells showed the decrease in baseline and glycolytic capacity compared to control.

Concerning the utilization of mitochondrial OXPHOS versus glycolysis as a source of cellular energy, RPE‐1 contact inhibition, p21‐induced senescence, and TMZ‐treated glioblastoma cells demonstrated an overall significant shift toward oxidative phosphorylation (OXPHOS) metabolism. Conversely, RPE‐1 IRIS and p16‐induced senescence led to a significant shift toward glycolysis as the cellular energy source (Fig. S6E).

**Fig. 6:**
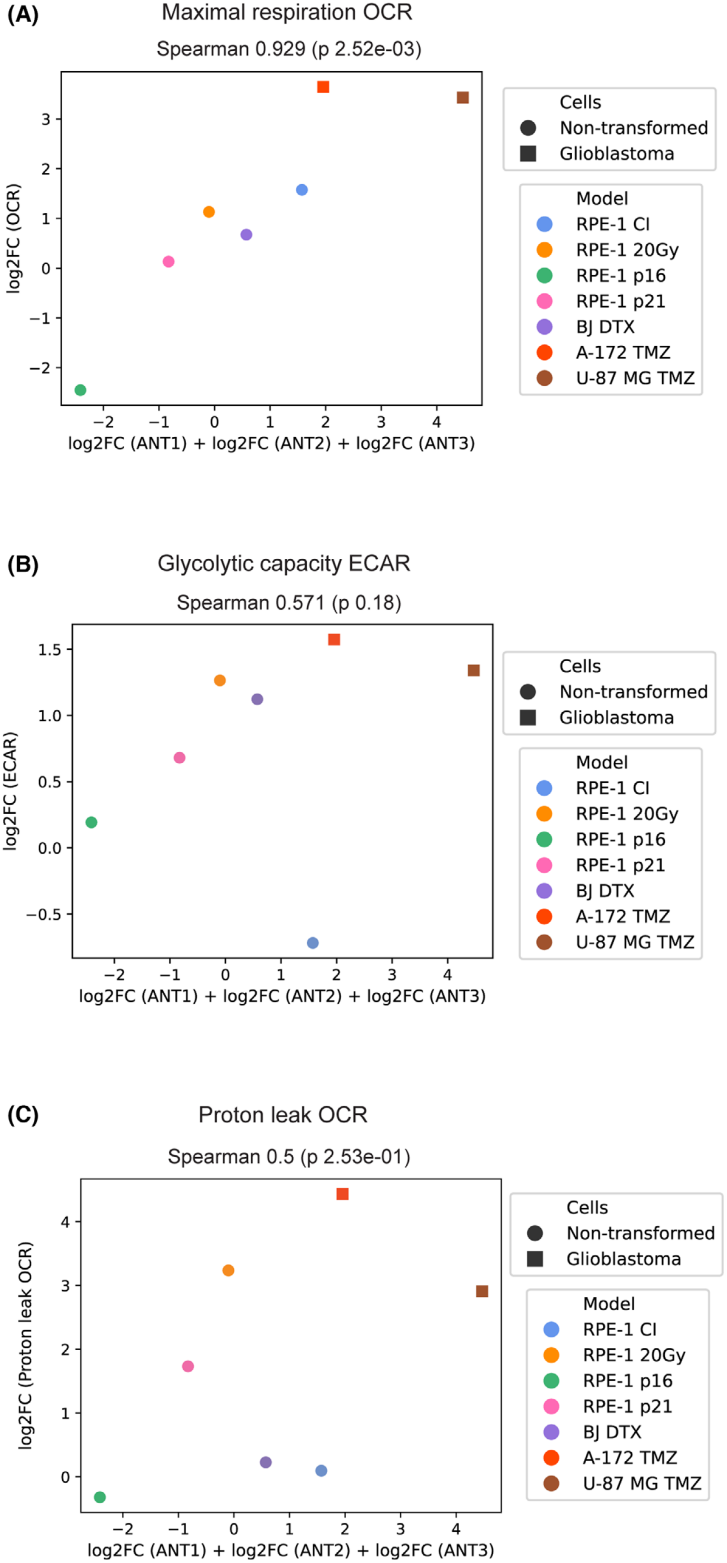
Correlation of ANT protein changes detected by *μ*LC‐PRM and maximal respiration OCR. ANT protein levels were measured in quiescent RPE‐1 (contact inhibition; CI) and different models of senescence (IRIS RPE‐1; 20 Gy), CDK‐inhibitor overexpression (p16) and (p21) in RPE‐1 cells, docetaxel‐induced senescence (DTX) in normal BJ fibroblasts and TMZ‐induced senescence in glioblastoma cell lines A‐172 and U‐87 MG. The pooled fold changes of ANT protein levels were correlated with maximal respiration OCR (A), glycolytic capacity ECAR (B), and proton leak OCR (C). Spearman's correlation coefficient (*Spearman*) and *P*‐value calculated from three experimental replicates are shown. (A–C) Spearman's rank correlation coefficient was used for statistics.

As our bioinformatic analyses revealed a link between the levels of ANT proteins and mitochondrial mass, we anticipated a correlation between ANT protein levels and OCR. Indeed, the pooled fold changes of ANTs exhibited a robust correlation (Spearman correlation 0.929, *P* = 2.52e‐03) with changes in OCR, suggesting that increased ANT proteins during the establishment of cellular senescence led to an elevated oxygen consumption rate (Fig. 6A). When analyzed individually (Fig. S6F), ANT1 protein level did not correlate with OCR, whereas ANT2 and ANT3 levels showed strong correlation (Spearman correlation 0.786, *P* = 0.036 and Spearman correlation 0.857, *P* = 0.014, respectively). Interestingly, our results indicate that ECAR does not correlate with either individual ANT proteins (Fig. S6G) or pooled ANT proteins (Fig. 6B). Since we observed increased proton leak OCR in senescent cells, we compared levels of ANTs with proton leak OCR. However, neither individual nor total ANT protein changes significantly correlate with changes in proton leak OCR (Fig. 6C and Fig. S6H).

In conclusion, senescent cells increase their overall metabolic activity. The cumulative changes in ANT protein levels correspond to alterations in the oxygen consumption rate following senescence induction.

### 3.7 Mitochondrial mass and ANT protein levels increase in glioblastoma cell line following temozolomide‐induced senescence

**Fig. 7:**
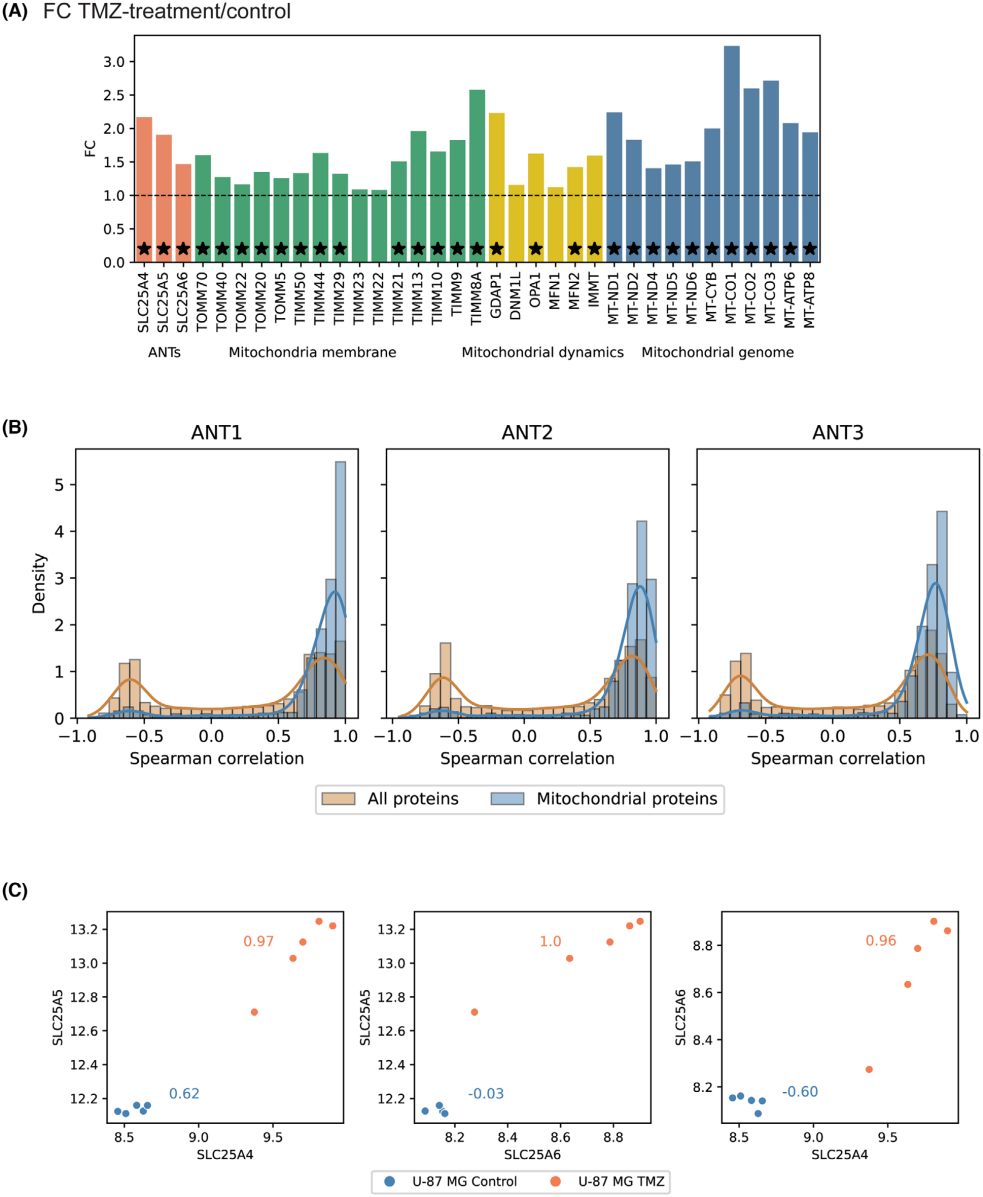
Proteomic analysis of U‐87 MG cells exposed to TMZ. (A) Changes in ANT proteins and proteins of mitochondria membrane, mitochondrial dynamics, and mitochondrial genome following TMZ treatment. Asterisks denote statistical significance according to Empirical Bayes moderated *t*‐statistics. (B) Spearman correlation of ANT proteins with all proteins and mitochondrial proteins. (C) Pearson correlation between individual ANT proteins in control U‐87 MG cells and post‐TMZ cells. Data were calculated from five experimental replicates, and each dot represents one experimental replicate.

To investigate the broader effects of senescence induction on changes in the cellular proteome, we analyzed the entire proteome of the human glioblastoma cell line (U‐87 MG) before and after treatment with TMZ (100 μm) using two‐dimensional liquid chromatography coupled to data‐dependent mass spectrometry analysis (2D LC–MS/MS). One thousand six hundred forty‐nine proteins were upregulated in the proteome of TMZ‐induced U‐87 MG cells. At the same time, 829 proteins were downregulated after treating cells with TMZ after 14 days of exposure. Among these, ANT1, ANT2, and ANT3 and mitochondrial genome‐encoded proteins (MT‐ND1, MT‐ND2, MT‐ND4, MT‐ND5, MT‐ND6, MT‐CYB, MT‐CO1, MT‐CO2, MT‐CO3, MT‐ATP6, MT‐ATP8) exhibited significant upregulation (Fig. 7A). Note that ANT4 was not detected. Additionally, all analyzed proteins related to mitochondrial dynamics (GDAP1, DNM1L, OPA1, MFN1, MFN2, IMMT) and mitochondrial membrane proteins (TOMM70, TOMM40, TOMM22, TOMM20, TOMM5, TIMM50, TIMM44, TIMM29, TIMM23, TIMM22, TIMM21, TIMM13, TIMM10, TIMM9, TIMM8A) were upregulated, with most showing significant changes (Fig. 7A). As in the case of glioblastoma and lung carcinoma dataset, correlations of ANT proteins are stronger with mitochondrial proteins (from MitoCarta3.0) than with all proteins (Fig. 7B). ANT proteins are even strongly correlated with most mitochondrial proteins. Notably, the intercorrelation between ANT proteins differed between control U‐87 MG cells and post‐TMZ cells (Fig. 7C). In control U‐87 MG cells, ANT1 exhibited a strong positive correlation with ANT2 and a strong negative correlation with ANT3. However, ANT2 and ANT3 were not correlated in control cells. Conversely, in post‐TMZ cells, all three ANTs showed strong positive intercorrelations, similar to our observation in the glioblastoma patient dataset. Considering TMZ‐induced expression of ANT1, ANT2, and ANT3, and according to our *μ*LC‐PRM results, the robust intercorrelation is induced by the increase in all ANTs together with an increase in total mitochondrial mass.

In conclusion, ANT proteins significantly correlate with mitochondrial proteins. In response to TMZ treatment, an increase in ANT protein levels co‐occurs with an increase of mitochondrial mass.

## 4 Discussion

ATP/ADP translocases are responsible for transporting ATP and ADP across mitochondrial membrane, playing a crucial role in cellular energy metabolism. Although ANT1, ANT2, and ANT3 isoforms share nearly 90% of the primary structure, some reports suggest they have distinct functions. Specifically, ANT1 and ANT3 facilitate the exchange of mitochondria‐produced ATP for cytosolic ADP during oxidative phosphorylation in differentiated cells. In contrast, ANT2 is associated with glycolytic metabolism, characteristic of growing normal and cancer cells. Proposed ANT2's specific role is the transport of ATP into mitochondria in exchange for ADP [49]. Beyond ATP/ADP transport, emerging evidence suggests that individual ANTs have additional roles, including opposing functions in regulating cell death. While ANT2 exhibits anti‐apoptotic activity, overexpression of ANT1 and ANT3 leads to a loss of mitochondrial membrane potential and activation of caspases [17]. Notably, repression of ANT1 and ANT2 has recently been linked to the cellular transition to senescence [17, 20], suggesting that ANTs play a role in energy metabolism changes associated with the onset of cellular senescence.

Previous studies suggest that components of the TGF‐beta signaling pathway actively repress ANT2 transcription during the induction of growth‐suppressive conditions *in vitro* [47], including cellular senescence [17]. However, an analysis of nine transcriptome datasets from different *in vitro* senescent cells obtained from publicly available databases confirmed a decrease in ANT2 transcript levels in senescent cells under only two conditions: erlotinib‐induced senescence of normal human bronchial epithelial (NHBE) cells and replicative senescence of human coronary artery endothelial cells (HCAECs). Interestingly, the transcriptome analysis of human benign tumors, where the presence of senescent cells is assumed, did not consistently show a decrease in the ANT2 transcript levels across all analyzed types, except for colon adenomas. Notably, significant differences in transcript levels were observed in all three ANT isoforms compared to normal tissues regarding upregulation and downregulation for all three ANT isoforms. A similar pattern emerges when examining different types of brain tumors.

Considering that transcript and protein levels do not always exhibit a positive correlation (see, e.g., [22]), we analyzed two datasets in which transcriptomes and proteomes were measured in tandem [38, 39]. Specifically, we correlated the transcript and protein levels of ANT1, ANT2, and ANT3. In the glioblastoma dataset, we observed a moderate correlation for ANT1, while in lung carcinoma, the correlation for ANT2 and ANT3 was weak. Interestingly, our *μ*LC‐PRM data from senescent models revealed a robust correlation between ANT2 and ANT3. It is worth noting that correlations between mRNA and protein abundances in large‐scale studies are generally moderate, and one of the factors influencing these correlations may be the reproducibility challenges associated with protein and transcript measurements [50]. These findings underscore that ANT protein levels do not always mirror their transcript levels.

We explored further correlations in both datasets to understand the regulatory mechanisms governing the levels of individual ANT isoforms. A surprising finding emerged: the levels of all three ANT isoforms are consistently maintained in proportion to the total proteome in glioblastoma and lung carcinoma. Additionally, ANT protein levels exhibit positive correlations with most mitochondrial proteins, such as mitochondria‐encoded components of the oxidative phosphorylation chain, proteins involved in regulating mitochondrial dynamics, and both inner and outer mitochondrial membrane proteins, including the transport system for the incorporation of mitochondrial proteins. These correlations suggest that the size of the mitochondrial compartment plays a pivotal role in determining ANT protein levels. The question of how these processes are mechanistically linked requires further research.

Interestingly, unlike normal brain tissue where the ratio of protein levels among individual ANT isoforms follows the order from highest to lowest (ANT1 to ANT3 and ANT2), glioblastoma and lung carcinoma exhibit variable ratios. However, this variability consistently correlates with mitochondrial mass, implying that individual ANT isoforms are interchangeable to maintain overall ATP/ADP transport capacity. An alternative explanation for this phenomenon could be the heterogeneity of the cell populations of the tumor tissue. Notably, a similar trend was observed in the temozolomide‐induced senescent U‐87 MG cell analysis. All three ANT isoforms correlated with mitochondrial mass, dynamics, and import machinery. Furthermore, in post‐TMZ cells, all three ANT isoforms showed robust intercorrelations.

Furthermore, we examined the relationship between the levels of individual ANT isoforms and the clinicopathological characteristics of individual patients with glioblastoma and lung cancer. In both cases, increased levels of ANTs correlated with tumor size at the time of diagnosis. Additionally, ANT2 mRNA positively correlated with the time from diagnosis to death. However, in the case of glioblastoma, tumor size did not correlate positively with adverse prognostic indicators such as Eastern Cooperative Oncology Group Performance Status (ECOG) score, KP score, and the number of days from diagnosis to death. These findings can be challenging to interpret. One possible explanation is that the compact and less diffuse growth of glioblastoma allows for more efficient surgical removal, leading to delayed disease recurrence. Simultaneously, larger tumors significantly impact the patient's physiological functions, resulting in a worse patient score. Alternatively, tumor size weakly but significantly negatively correlates with age, weight, and BMI, suggesting that patients with larger tumors are typically younger and leaner, potentially contributing to their longer life expectancy.

Since ANT protein levels do not reliably correlate with their transcript levels, mRNA‐based analysis cannot be used to assess ANT protein levels. Instead, quantifying proteins based on relative peptide levels processed through tryptic digestion offers a preferable approach. This method quantifies proteins based on unique peptide sequences specific to the corresponding protein or isoform [51]. However, tandem mass spectra generation in data‐dependent acquisition mode relies on ion signal intensity, resulting in the sequencing of only the most intense peptide precursors. This stochastic data collection approach is less effective when consistent MS detection of specific proteins is required. Particularly for downregulated proteins, there is a risk of missing critical information in the final dataset. To address this, parallel reaction monitoring (PRM) is a highly selective method for targeted protein quantification. PRM simultaneously monitors all fragmented ions of selected peptide precursors [52, 53]. In assessing ANT protein levels, targeted PRM monitoring of ANTs has proven valuable, as demonstrated herein in biological settings such as the development of IRIS. We exploited adjustments of the conventional (*μ*)LC‐MS system operating at the flow rate of tens μL·min^−1^, which had previously demonstrated superior performance in bottom‐up proteomic studies [52]. The *μ*LC‐PRM assessment offers a robust and time‐effective platform for targeted monitoring ANTs, requiring only 6 μg of cell lysate.

To validate the *μ*LC‐PRM‐based detection of ANT proteins, we analyzed changes in ANT isoforms during the establishment of IRIS in RPE‐1 cells. Our findings confirmed that the mRNA level of ANT2 declined during IRIS development, and a downregulation of ANT2 protein level accompanied this reduction in ANT2 transcript. Interestingly, the decrease of ANT2 protein levels persisted throughout the time course of senescence development, extending to at least day 32. This observation underscores that the development of the senescent phenotype is a long‐term process. Targeted monitoring of all three ANT isoforms revealed that the downregulation of ANT2 and ANT3 was followed by the increase of ANT1 protein levels and an increase in OCR characteristics, suggesting possible compensation between ANT isoforms and a change of energy metabolism. Previously, ANT2 deletion in HEK‐293 cells led to a decrease in maximal respiration. However, basal respiration, spare capacity, and ATP‐linked OCR were not affected. Interestingly, ANT2 deletion led to a decrease in proton leak OCR. It was hypothesized that the low levels of other ANT isoforms or other translocators are sufficient for basal cell metabolism [54]. Interestingly, ANT3 deletion in human conditionally immortalized renal proximal tubule epithelial cells led to a decrease of number of mitochondria per cell, but mitochondrial area per cell remained unaffected [55]. Furthermore, Hoogstraten *et al*. demonstrated that the deletion of ANT3 does not increase the sensitivity of cells to ANT inhibition, suggesting functional compensation among ANT isoforms [55]. Other studies have also described compensation at the protein level after the knockdown of individual ANT isoforms [56, 57], emphasizing the need to closely monitor changes in the level of other ANT isoforms. Our data similarly suggest that the overall increase in ANT isoforms, rather than any individual isoform, is responsible for the elevation in oxygen consumption rate during the development of cellular senescence, further highlighting the interchangeability of individual isoforms. Notably, in mice with deletion of all somatic ANT isoforms, murine ANT4 isoform (corresponding to human ANT4) expression is induced in somatic cells [56].

Generally, we observed metabolic upregulation in all tested senescent models apart from RPE‐1 cells expressing p16. Overexpression of p16 reduced maximal respiration capacity, consistent with the previously described increase in maximal respiration capacity following the downregulation of p16 [58]. The observed hypermetabolic phenotype of the senescent cells is consistent with other reports [59, 60, 61]. Furthermore, quiescent RPE‐1 cells exhibited lower levels of glycolysis and higher levels of maximal respiration, indicating a more energy‐efficient metabolism in quiescent cells. This finding aligns with previous reports on quiescent melanoma cells [62]. It is widely accepted that mitochondria increase in size and volume in senescent cells. However, this increase in mitochondrial mass primarily results from the accumulation of dysfunctional mitochondria [63]. Consequently, the elevated OXPHOS OCR was observed in all herein analyzed models except p16‐induced senescence. This finding aligns with numerous other reports indicating mitochondrial dysfunctions and OXPHOS deficiency in senescent cells, as recently reviewed elsewhere [64]. Interestingly, proton leak OCR contributed to the total increase of OXPHOS OCR. However, we did not find a significant correlation between changes in individual or total ANT levels and proton leak OCR, despite the known role of ANTs as proton gradient uncouplers [65, 66]. Previously, ANT2 expression was associated with glycolytic metabolism [2, 3, 4, 18, 67]. However, we did not observe the association of ANT2 with glycolysis measured in ECAR analysis in our dataset. Since the expression of CDK inhibitors during senescence establishment also affects cellular metabolism [58, 62], the relationships between ANTs, cellular senescence, and metabolism are complex and require further study.

Consistently with our findings, an increase in mitochondrial volume with a reduction of mitochondrial OXPHOS activity and an upregulation of a considerable number of mitochondrial proteins (ANT1–3, proteins of mitochondrial genome, metabolism, dynamics, and signaling) was observed in recent mitochondrial proteome analysis of senescent cells [68]. On the other hand, small translocase proteins of the inner mitochondrial membrane (TIMM9, TIMM10, TIMM13) were downregulated in senescent cells, and translocase proteins of the outer mitochondrial membrane (TOMM20, TOMM22, TOMM40, TOMM70) had no significant change in this study. However, these proteins in our proteomic analysis of U‐87 MG cells were upregulated after TMZ similar to other mitochondrial proteins [68].

Our data suggest that all ANT1, ANT2, and ANT3 isoforms contribute to increased respiration during the induction of senescence. Changes in their total protein levels rather than changes in a specific isoform correlate with alterations in maximal respiration OCR (Fig. 6A), supporting their interchangeability for respiration. The interchangeability is not surprising, given that ANT1, ANT2, and ANT3 exhibit similar kinetic properties when expressed in yeast [69].

Considering that the mitochondrial inner membrane is saturated with proteins [70], we anticipate that the increase in the total ANT pool is primarily linked to an expansion of total mitochondrial mass within the cell. This inference is supported by the correlation between ANT proteins and other mitochondrial proteins in our analysis of human normal and tumor proteomic data. In prostate cancer cells, ANT2 was found to be deacetylated by SIRT4 at lysine 105, which promoted ubiquitin‐mediated ANT2 degradation [71]. In mice, ANT1 and ANT2 were shown to inhibit translocase TIM23, leading to the stabilization of PINK1, which targets mitochondria to mitophagy by recruiting E3 ubiquitin ligase Parkin [72]. In human diploid dermal fibroblasts, ANT2 overexpression induces increased mitophagy [18]. For a review of the role of ANTs in mitophagy, see [15]. Therefore, ANT proteins regulate total mitochondrial mass, which probably contributes to the high correlation between ANT expression and total mitochondrial mass observed in our analysis. Furthermore, since ANT proteins stalled during mitochondrial import are cleared not by the proteasome but by mitochondrial protease Yme1p in yeast [73], we expect that intramitochondrial processes play an important role in sustaining ANT levels in human mitochondria. Taken together, the complex interplay between ANT and mitophagy likely contributes to the lack of tight regulation of ANT expression at the transcript level, which manifests in poor mRNA–protein correlation of mitochondrial proteins.

The increase in ANT1 protein level observed in all senescent models used in our study is intriguing, given that ANT1 is typically considered a pro‐apoptotic protein. However, compensatory changes in other components of the mitochondrial permeability transition pore (MPTP) may mitigate the pro‐apoptotic effect of ANT1. While ANT proteins are not essential for MPTP formation, they regulate it, as extensively reviewed [15]. Notably, ANT1 has been identified as the voltage sensor of the MPTP [74]. In senescent cells, MPTP opening increases, and ROS production triggers this process. Subsequently, MPTP‐induced ROS production forms a ‘vicious cycle’ [75]. Interestingly, senescent cells exhibit higher resistance to apoptosis [76], and the paradoxical protective roles of short‐term, infrequent MPTP opening may contribute to this resistance [77]. Alternatively, ANT1 overexpression might enhance the survival of cancer cells under oxygen‐restricted conditions. For instance, in rat hearts, ANT1 overexpression reduces ROS production and oxidative stress during ischemia, ultimately increasing the survival rate of infarcted rats [78].

## 5 Conclusions

This study analyzed transcriptome and proteome datasets from patients with glioblastoma and lung carcinoma to assess ANT transcript and protein levels. We found a weak correlation between ANT transcript and protein levels, but a robust correlation between mitochondrial mass and the mitochondrial import machinery.

To accurately measure the levels of individual ANT isoforms, we developed a *μ*LC‐PRM tool, which allowed us to determine the stoichiometric ratios of individual ANT isoforms. Using several *in vitro* models of cellular senescence, we demonstrated that the development of senescence is accompanied by a hypermetabolic state, characterized by a global increase in ANT protein levels, particularly ANT1. During this transition, ANTs are regulated post‐transcriptionally rather than at the transcription level.

## Conflict of interest

The authors declare no conflict of interest.

## Author contributions

ZL: Investigation, Writing—Original Draft, Visualization, DM: Formal analysis, Investigation, Writing—Original Draft, Writing—Review and Editing, Visualization, BS: Investigation, Conceptualization, Methodology, Formal analysis, Investigation, Data Curation, Writing—Review and Editing, MK: Investigation, Visualization, TM: Resources, Project administration, ZK: Investigation, AP: Investigation, PV: Investigation, Writing—Review and Editing, Visualization, DR: Investigation, LA: Resources, IF: Methodology, RK: Methodology, Data Curation, PK: Investigation, PS: Investigation, VT: Conceptualization, JB: Conceptualization, Resources, Project administration, JN: Conceptualization, Writing—Original Draft, Writing—Review and Editing, Supervision, MV: Conceptualization, Methodology, Formal analysis, Resources, Writing—Original Draft, Writing—Review and Editing, Visualization, Project administration, ZH: Conceptualization, Methodology, Resources, Writing—Original Draft, Writing—Review and Editing, Supervision, Project administration, Funding acquisition.

## Supporting information


**Fig. S1.** Changes in ANTs transcripts extracted from six publicly available datasets and their expression in single‐cell transcriptomic data of glioblastoma.
**Fig. S2.** Protein and mRNA correlation in glioblastoma and lung carcinoma.
**Fig. S3.** Selection of potential ANT peptides and optimization of μLC‐PRM method using ionizing radiation‐induced senescence in RPE‐1 cells.
**Fig. S4.** Validation of specificity of anti‐ANT antibodies and of senescence induction in IRIS RPE‐1.
**Fig. S5.** Detection of β‐galactosidase activity and ANT1 and ANT2 protein levels using immunoblotting in different senescence models.
**Fig. S6.** Characteristics of cellular energy metabolism in different senescence models.
**Table S1.** Unique peptides selected for the targeted quantification of ANT1, ANT2, and ANT3 isoforms by *μ*LC‐PRM.
**Table S2.** Unique peptides rejected from the targeted quantification of ANT1, ANT2, and ANT3 isoforms by *μ*LC‐PRM.
**Table S3.** Forward and reverse primers used for quantification of ANTs transcripts.

## Data Availability

The survey mass spectrometry data have been deposited to the ProteomeXchange Consortium via the PRIDE [79] partner repository with the dataset identifier PXD029523. The Skyline documents containing the targeted mass spectrometry data have been deposited on the Panorama server [80] and can be accessed on the Panorama Public website (https://panoramaweb.org/IRIS_ANT_switch_monitoring.url). Publicly available transcriptome databases analyzed in this study are listed in Table 1. Single‐cell transcriptomics data can be accessed at http://gbm.cells.ucsc.edu. The publicly available proteome‐transcriptome data used in this publication were generated by the Clinical Proteomic Tumor Analysis Consortium (CPTAC) and are available on the CPTAC Data Portal at: https://cptac‐data‐portal.georgetown.edu/cptac/s/S048 and Genomic Data Commons (GDC) at: https://portal.gdc.cancer.gov/projects/CPTAC‐3. A list of mitochondrial genes used is available in the MitoCarta3.0 inventory [41].
