# Supplementary material for: Determination of ADP/ATP translocase isoform ratios in malignancy and cellular senescence
Source: Mol Oncol. 2025 Apr 27;19(9):2619–47. doi: 10.1002/1878-0261.70039 (PMC12420346; doi:10.1002/1878-0261.70039)
Supplement: Supplementary file 1 — Fig. S1. Changes in ANTs transcripts extracted from six publicly available datasets and their expression in single‐cell transcriptomic data of glioblastoma. Fig. S2. Protein and mRNA correlation in glioblastoma and lung carcinoma. Fig. S3. Selection of potential ANT peptides and optimization of μLC‐PRM method using ionizing radiation‐induced senescence in RPE‐1 cells. Fig. S4. Validation of specificity of anti‐ANT antibodies and of senescence induction in IRIS RPE‐1. Fig. S5. Detection of β‐galactosidase activity and ANT1 and ANT2 protein levels using immunoblotting in different senescence models. Fig. S6. Characteristics of cellular energy metabolism in different senescence models. Table S1. Unique peptides selected for the targeted quantification of ANT1, ANT2, and ANT3 isoforms by μLC‐PRM. Table S2. Unique peptides rejected from the targeted quantification of ANT1, ANT2, and ANT3 isoforms by μLC‐PRM. Table S3. Forward and reverse primers used for quantification of ANTs transcripts. [file MOL2-19-2619-s001.pdf]

Supplemental document to original research article

Determination of ADP/ATP translocase isoform ratios in malignancy and cellular senescence

Zuzana Liblova[1$], Dominika Maurencova[1$], Barbora Salovska[1+], Marek Kratky[1], Tomas Mracek[2], Zuzana Korandova[2#], Alena Pecinova[2], Pavla Vasicova[1], David Rysanek[1], Ladislav Andera[1], Ivo Fabrik[3], Rudolf Kupcik[3], Pavel Kashmel[1], Pinky Sultana[1], Vojtěch Tambor[3], Jiri Bartek[1,4,5], Josef Novak[1*], Marie Vajrychova[1*], and Zdenek Hodny[1*]

[1]Laboratory of Genome Integrity, Institute of Molecular Genetics of the Czech Academy of Sciences, Prague, Czech Republic

[2]Laboratory of Bioenergetics, Institute of Physiology of the Czech Academy of Sciences, Prague, Czech Republic

[3]Biomedical Research Center, University Hospital Hradec Kralove, Hradec Kralove, Czech Republic

[4]Danish Cancer Society Research Center, Copenhagen, Denmark

[5]Department of Medical Biochemistry and Biophysics, Division of Genome Biology, Science for Life Laboratory, Karolinska Institute, Stockholm, Sweden

[$]equal contribution

*Corresponding authors: Zdenek Hodny, MD, PhD; Laboratory of Genome Integrity, Institute of Molecular Genetics of the Czech Academy of Sciences, Videnska 1083, CZ 142 20 Prague 4, Czech Republic; Tel: (420)241 063 151; E-mail: hodny@img.cas.cz, and Marie Vajrychova, PhD; Biomedical Research Center, University Hospital Hradec Kralove, Sokolska 581, 500 05 Hradec Kralove, Czech Republic; Tel: (420)495 832 017; E-mail: marie.vajrychova@fnhk.cz, and Josef Novak, PhD; Laboratory of Genome Integrity, Institute of Molecular Genetics of the Czech Academy of Sciences, Videnska 1083, CZ 142 20 Prague 4, Czech Republic; Tel: (420)241 063 159; E-mail: josef.novak@img.cas.cz.

[+]Current affiliation: Yale Cancer Biology Institute, Yale University, West Haven, CT, USA

[#]Department of Adipose Tissue Biology, Institute of Physiology of the Czech Academy of Sciences, Prague, Czech Republic

Supplementary figures

Supplementary Figure 1

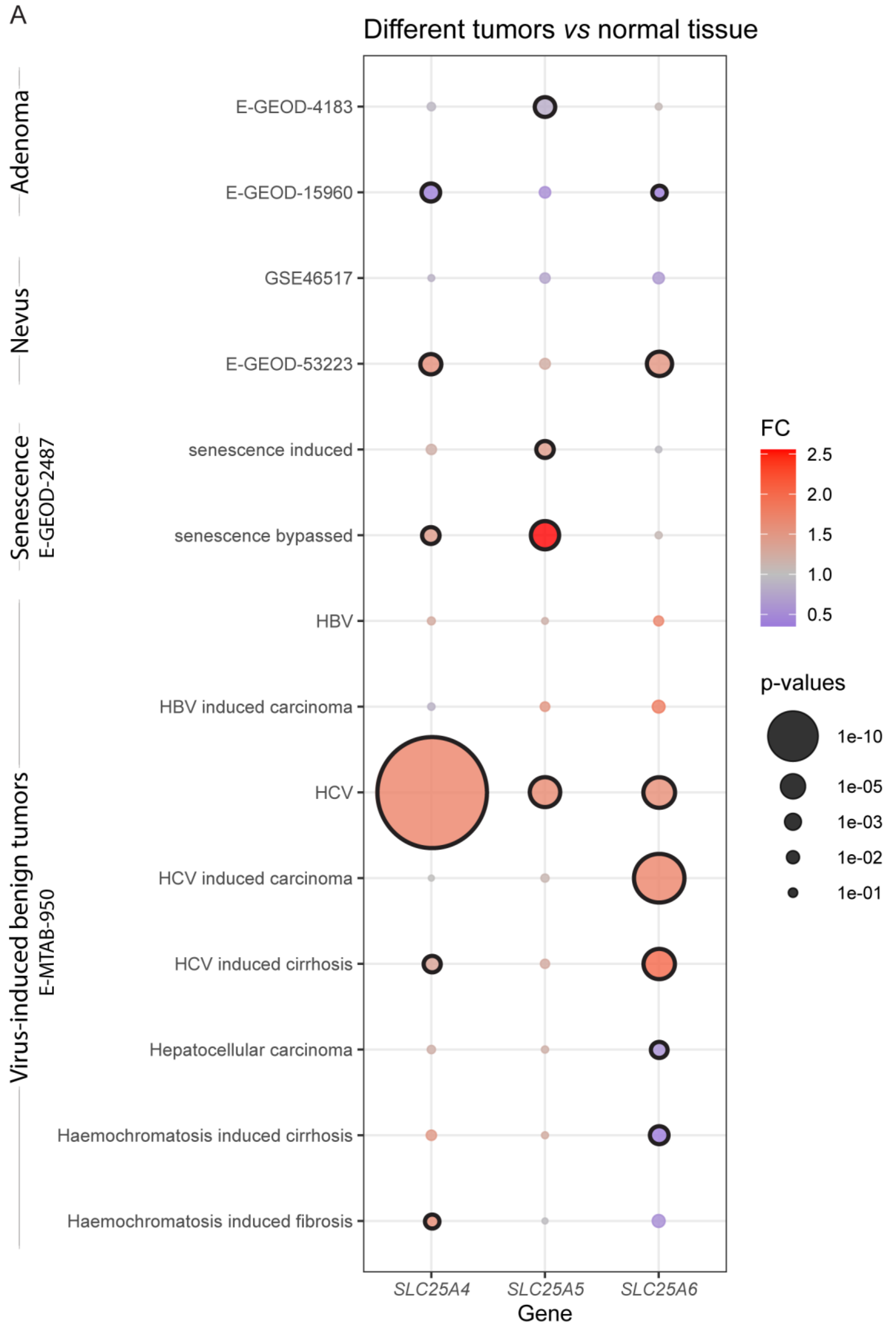

Supplementary Figure 1

B

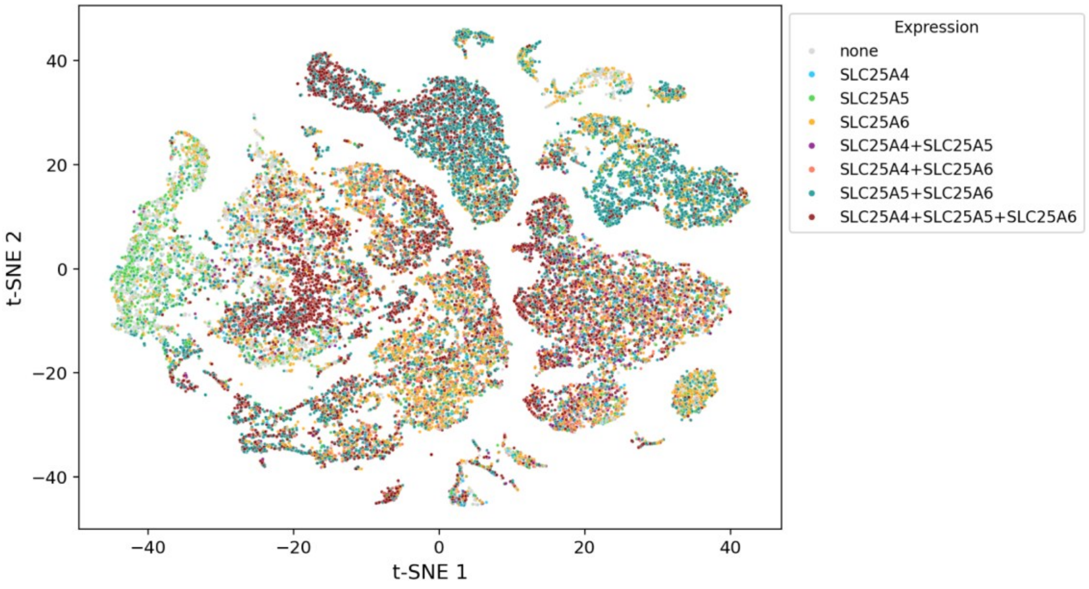

C

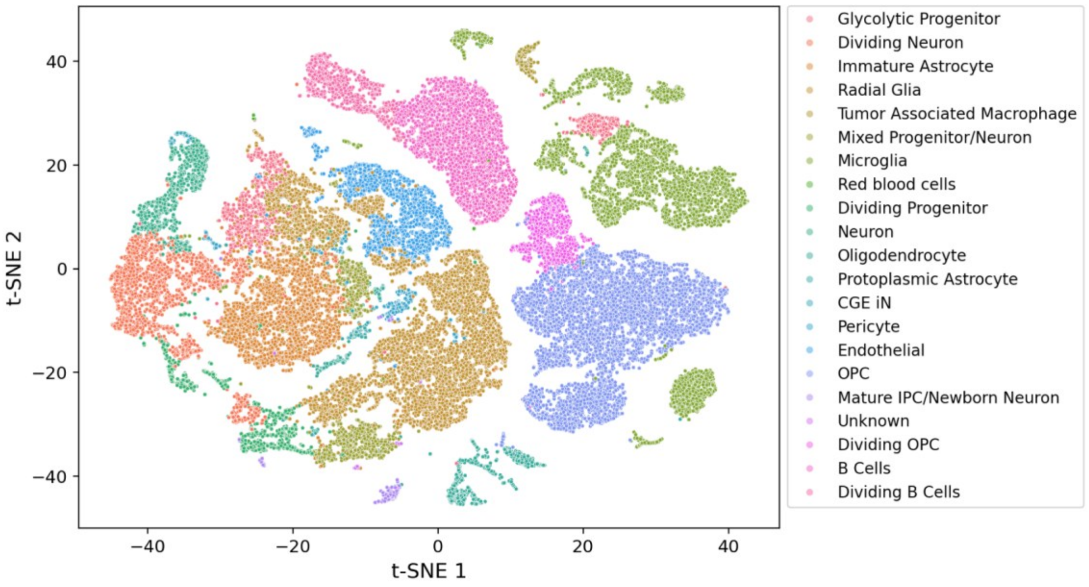

Supplementary Figure 2

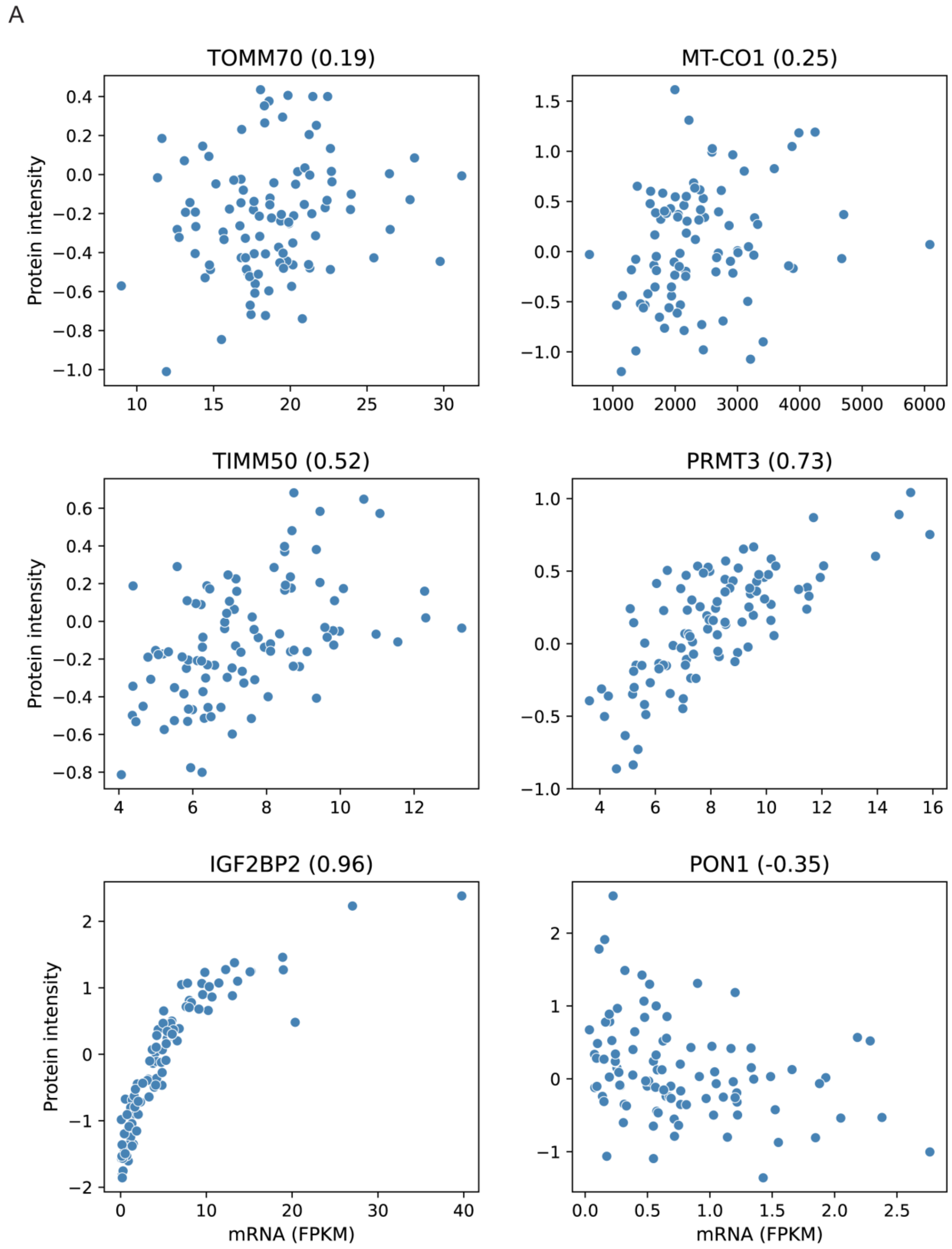

Supplementary Figure 2 (continued)

B

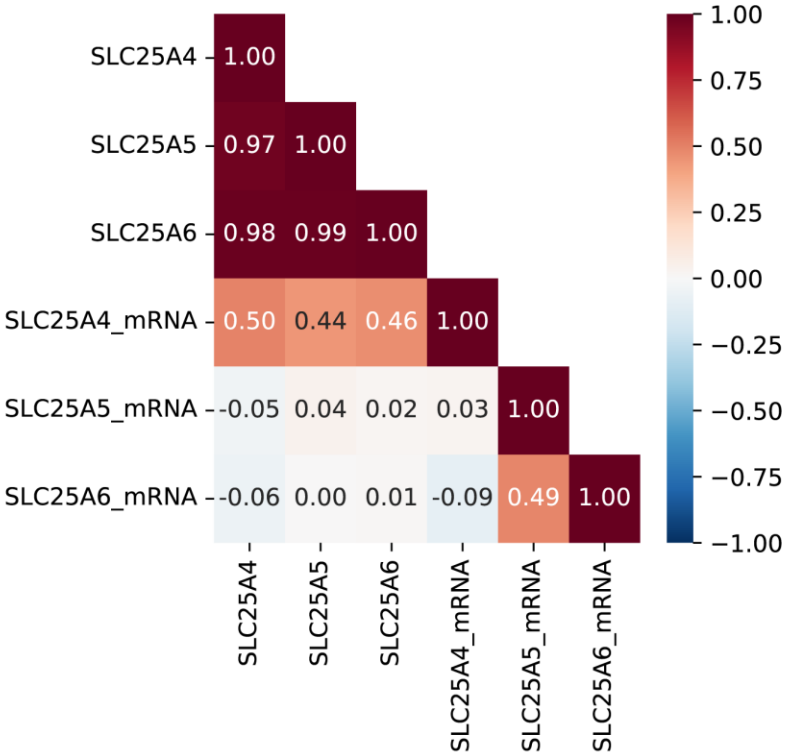

Supplementary Figure 2 (continued)

C

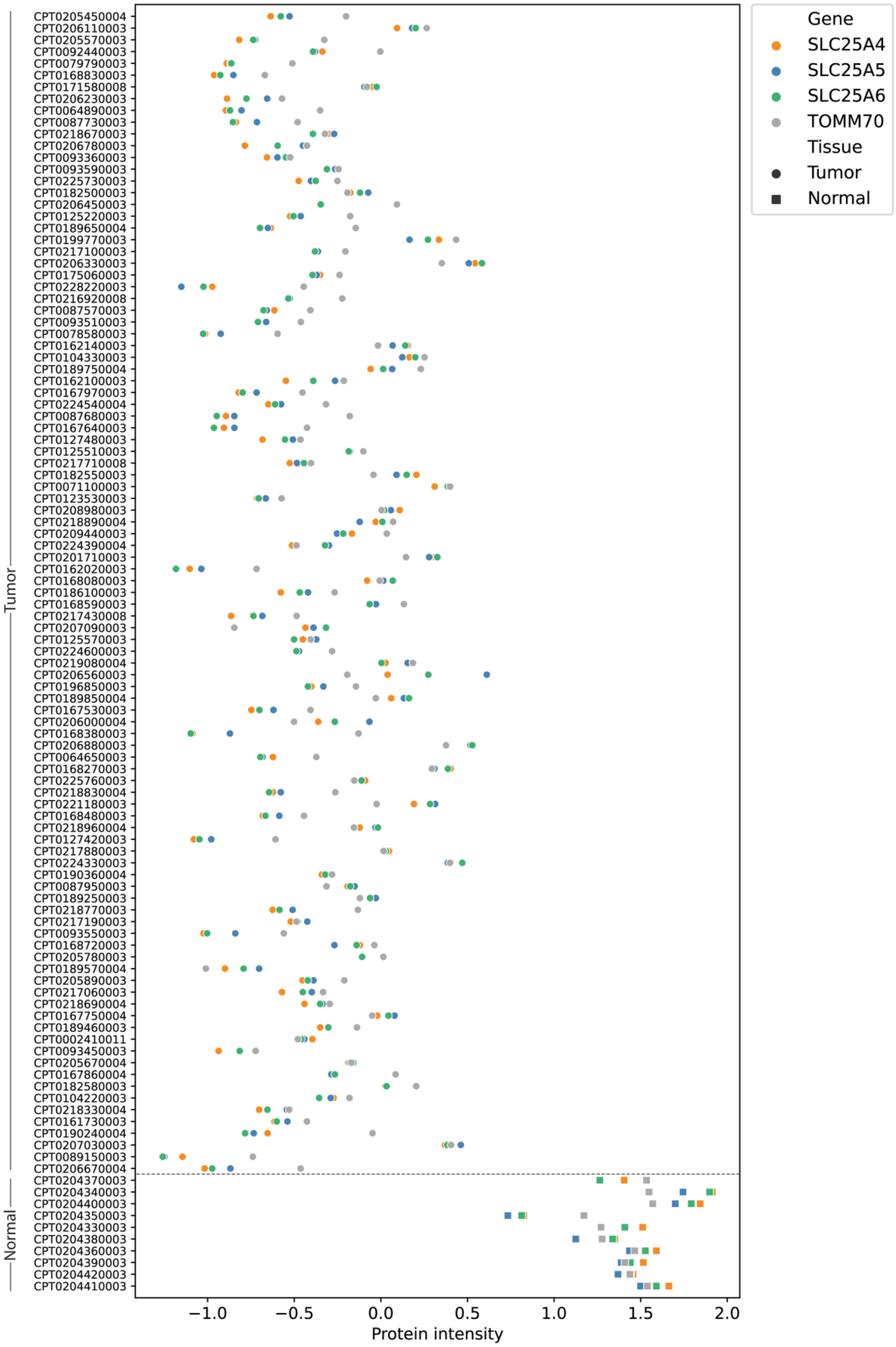

Supplementary Figure 2 (continued)

D

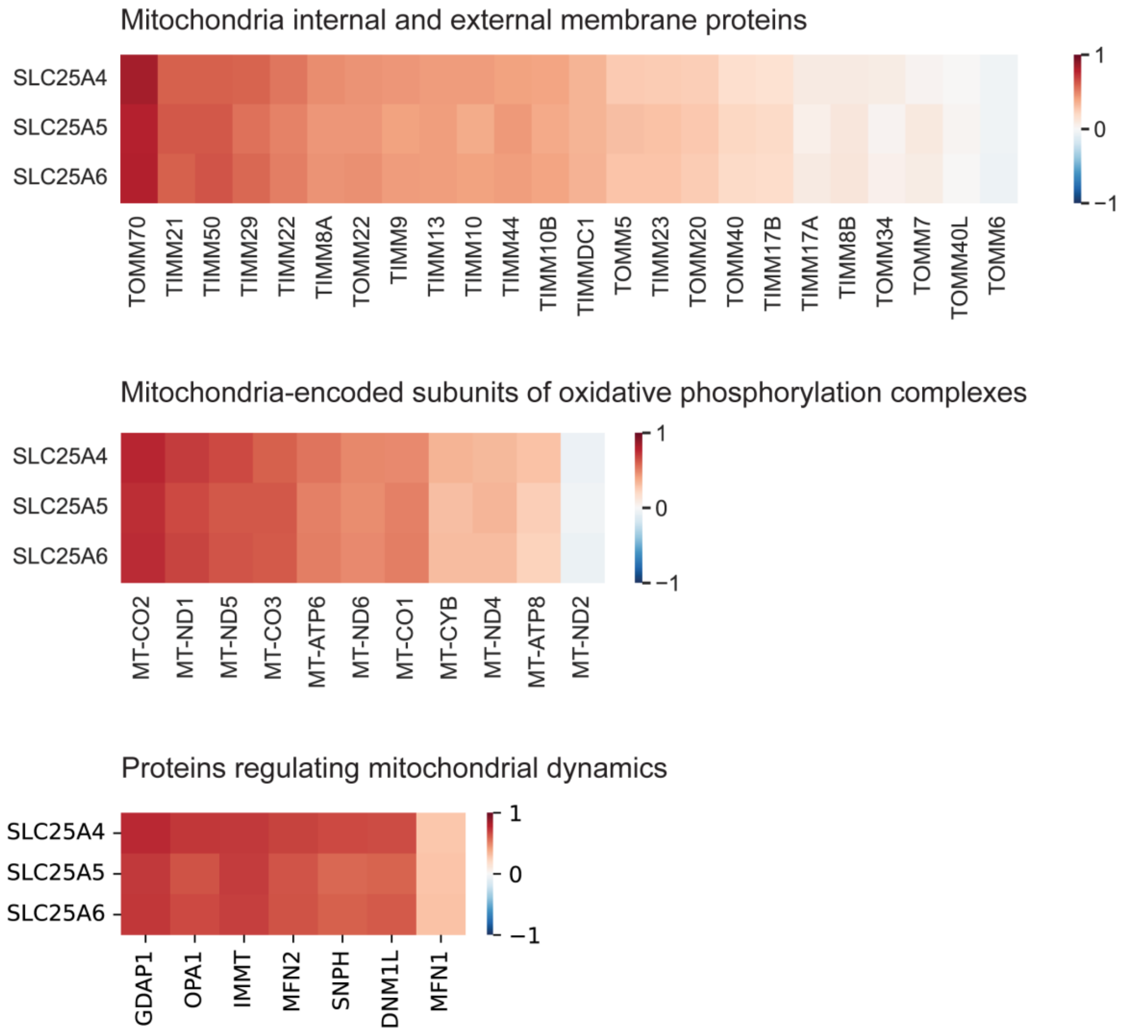

Supplementary Figure 2 (continued)

E

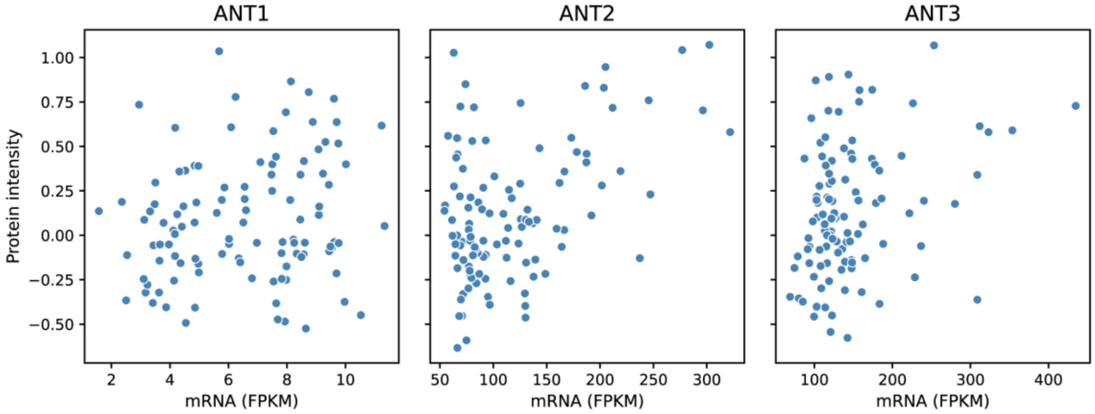

F

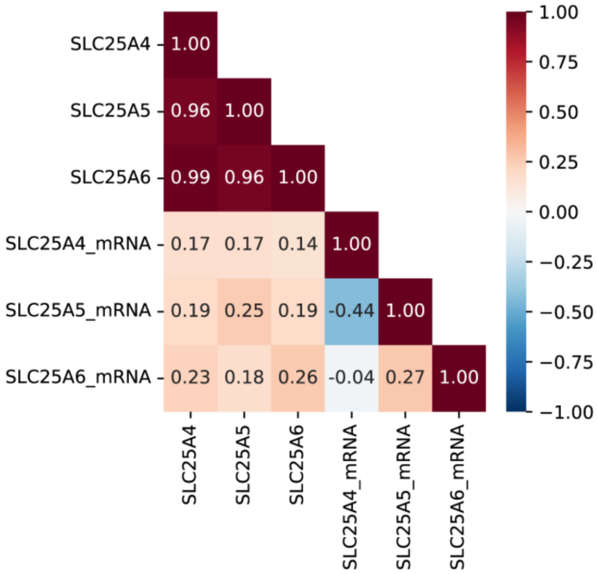

G

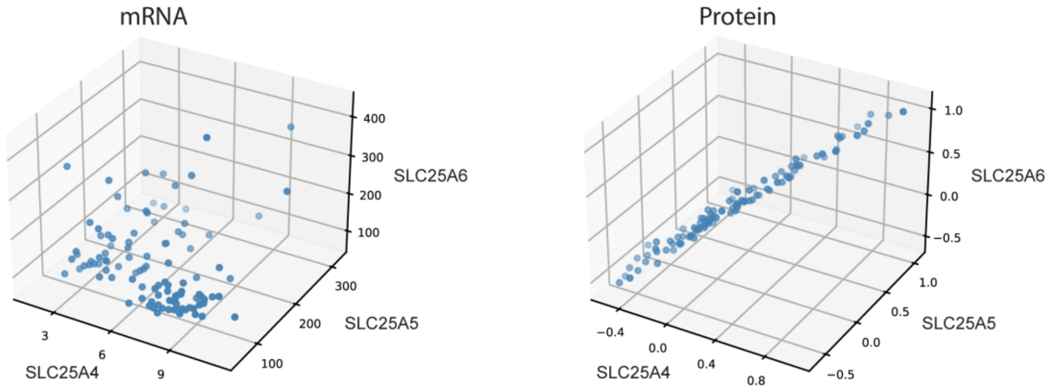

Supplementary Figure 2 (continued)

H

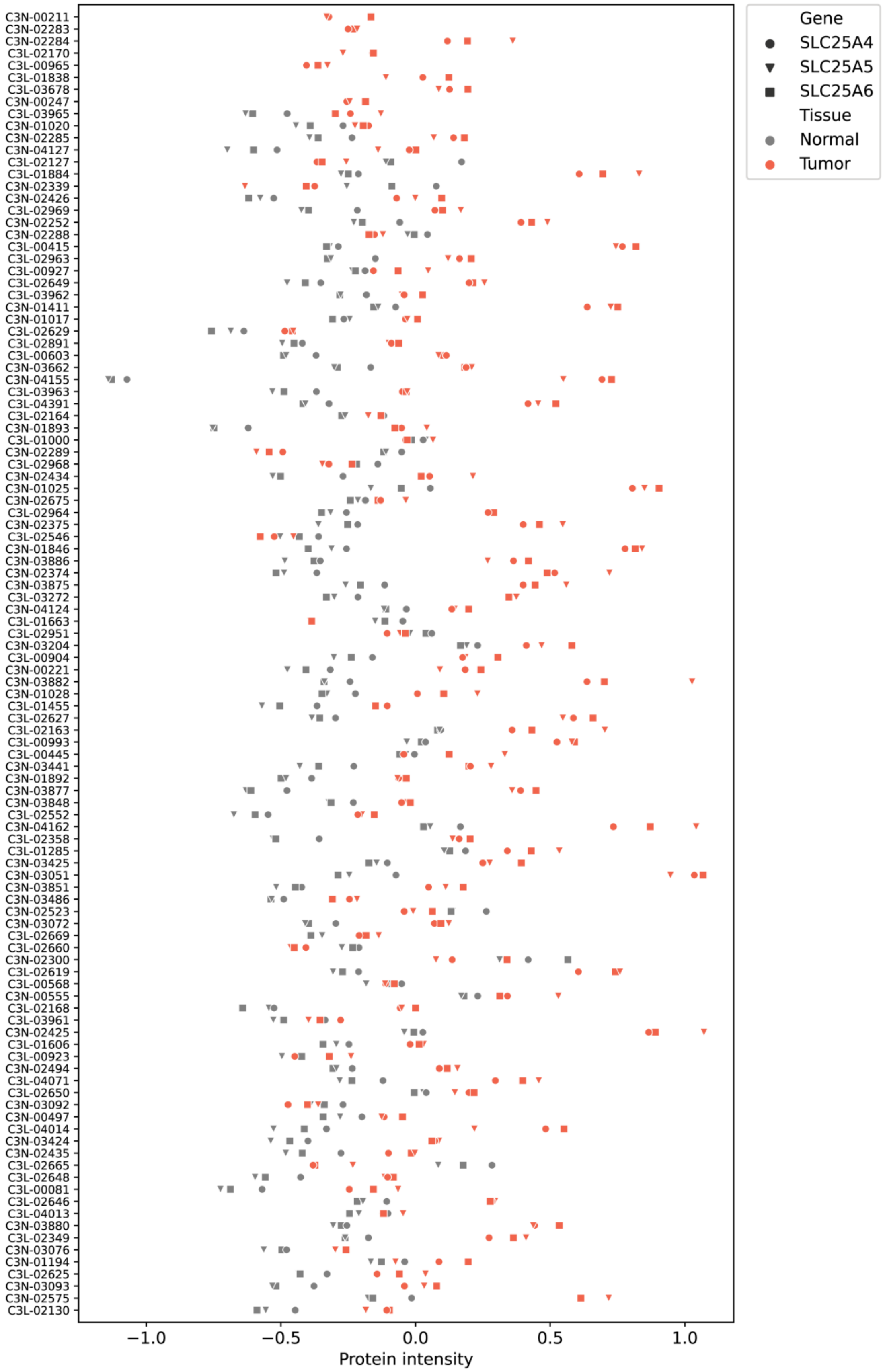

Supplementary Figure 2 (continued)

I

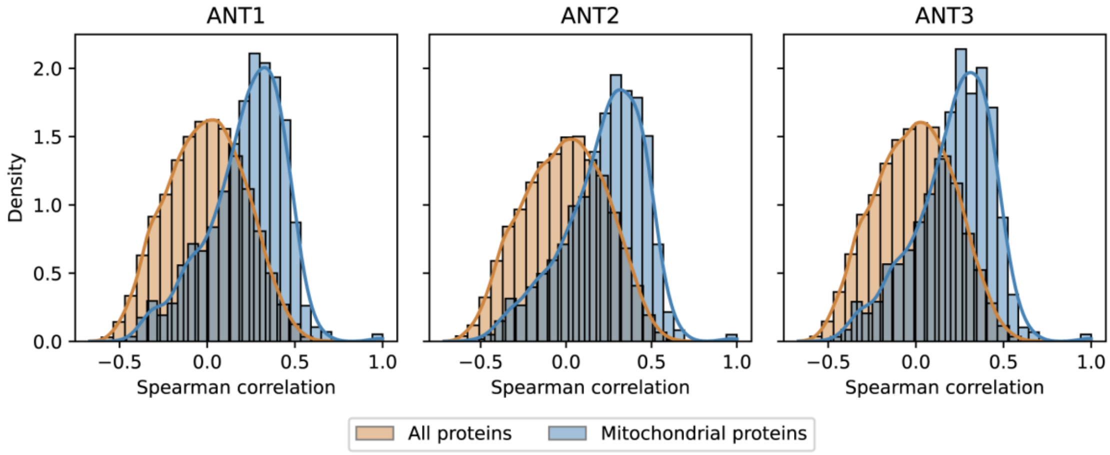

J

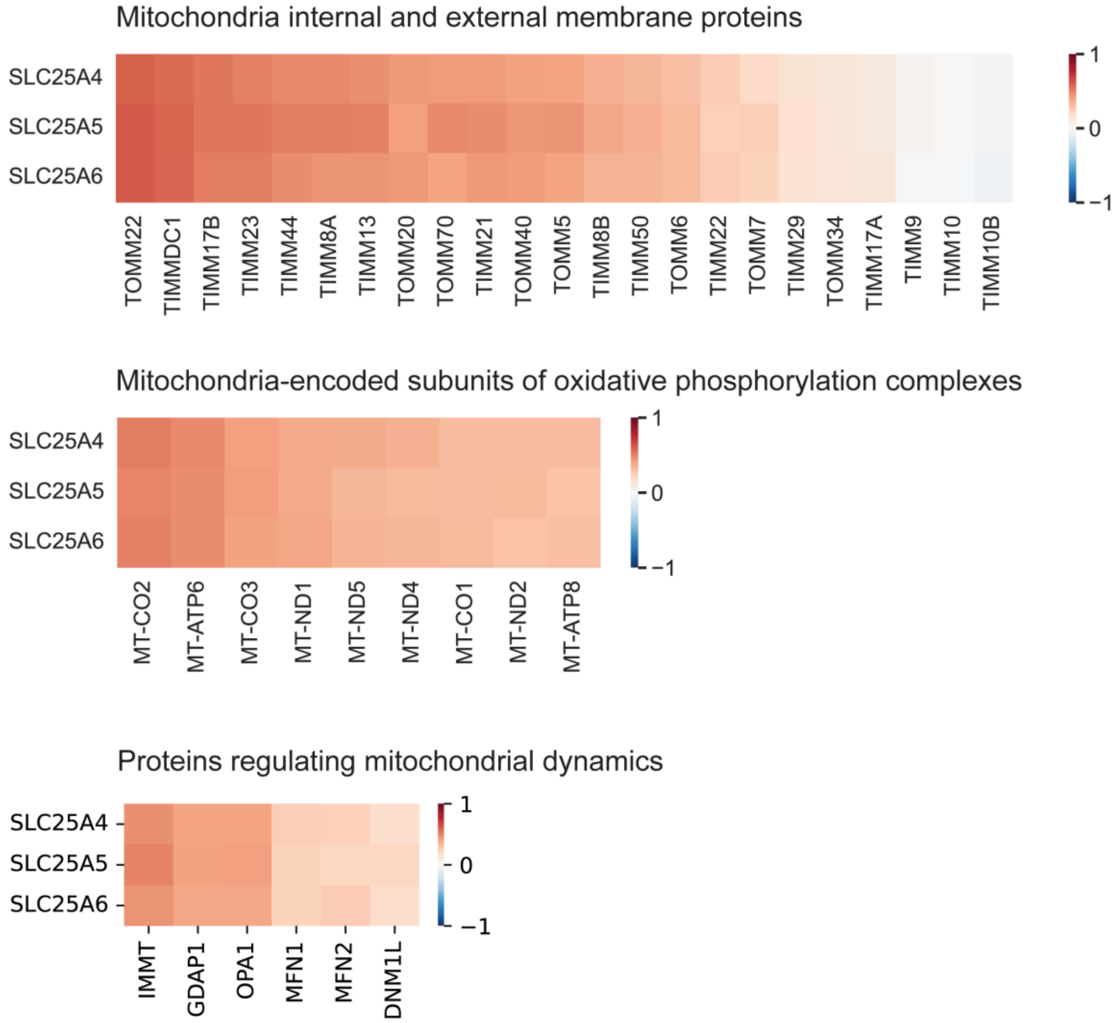

Supplementary Figure 2 (continued)

K

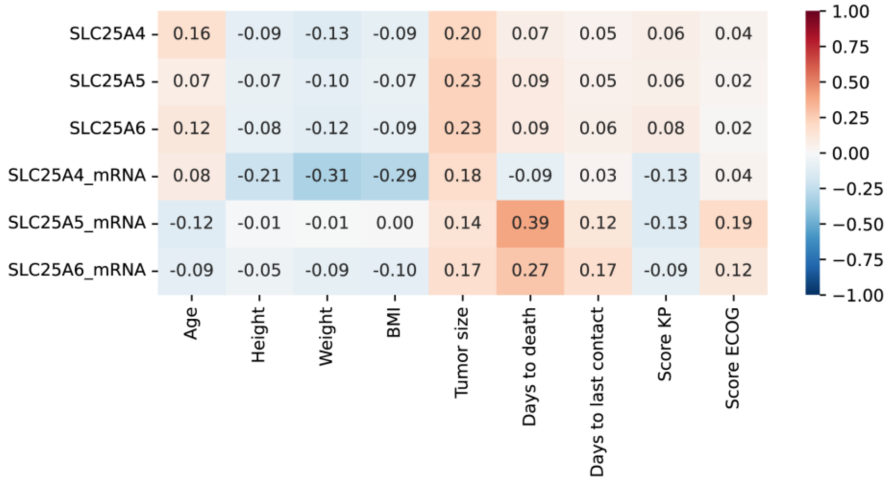

L

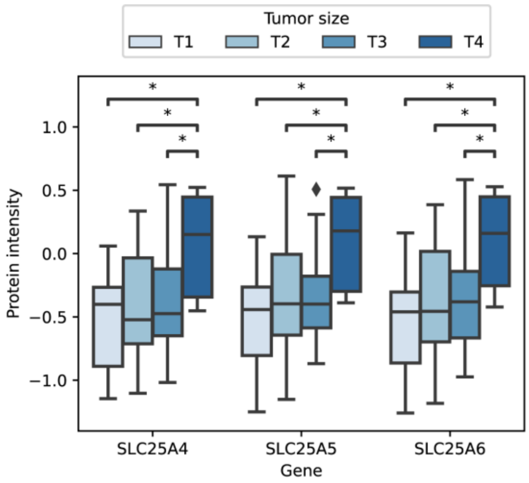

M

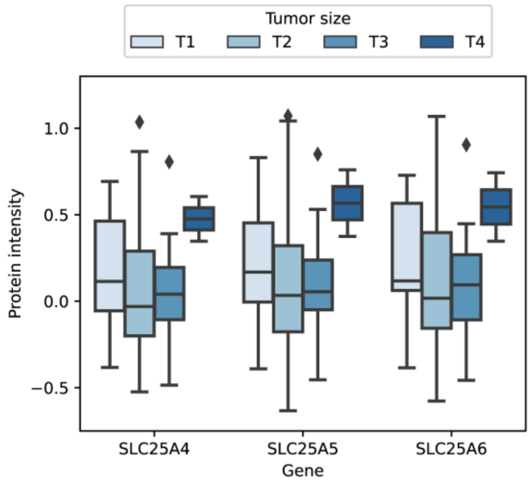

Supplementary Figure 3

A

Sequence alignment of human ANT isoforms

```
CLUSTAL O(1.2.4) multiple sequence alignment


Q9H0C2       MHREPAKKKAEKRLFDASSFGKDLLAGGVAAAVSKTAVAPIERVKLLLQVQASSKQISPE    60
P12235       ------------MGDHAWSFLKDFLAGGVAAAVSKTAVAPIERVKLLLQVQHASKQISAE    48
P05141       ------------MTDAAVSFAKDFLAGGVAAAISKTAVAPIERVKLLLQVQHASKQITAD    48
P12236       ------------MTEQAISFAKDFLAGGIAAAISKTAVAPIERVKLLLQVQHASKQIAAD    48
                             * ** **:****:***:****************** :****: :

Q9H0C2       ARYKGMVDCLVRIPREQGFFSFWRGNLANVIRYFPTQALNFAFKDKYKQLFMSGVNKEKQ    120
P12235       KQYKGIIDCVVRIPKEQGFLSFWRGNLANVIRYFPTQALNFAFKDKYKQLFLGGVDRHKQ    108
P05141       KQYKGIIDCVVRIPKEQGVLSFWRGNLANVIRYFPTQALNFAFKDKYKQIFLGGVDKRTQ    108
P12236       KQYKGIVDCIVRIPKEQGVLSFWRGNLANVIRYFPTQALNFAFKDKYKQIFLGGVDKHTQ    108
              :***::**:****:***.:*****************************:*:.**::..*

Q9H0C2       FWRWFLANLASGGAAGATSLCVVYPLDFARTRLGVDIGKGPEERQFKGLGDCIMKIAKSD    180
P12235       FWRYFAGNLASGGAAGATSLCFVYPLDFARTRLAADVGKGAAQREFHGLGDCIIKIFKSD    168
P05141       FWLYFAGNLASGGAAGATSLCFVYPLDFARTRLAADVGKAGAEREFRGLGDCLVKIYKSD    168
P12236       FWRYFAGNLASGGAAGATSLCFVYPLDFARTRLAADVGKSGTEREFRGLGDCLVKITKSD    168
             ** :* .**************.***********..*:**.  :*:*:*****::** ***

Q9H0C2       GIAGLYQGFGVSVQGIIVYRASYFGAYDTVKGLLPKPKKTPFLVSFFIAQVVTTCSGILS    240
P12235       GLRGLYQGFNVSVQGIIIYRAAYFGVYDTAKGMLPDPKNVHIFVSWMIAQSVTAVAGLVS    228
P05141       GIKGLYQGFNVSVQGIIIYRAAYFGIYDTAKGMLPDPKNTHIVISWMIAQTVTAVAGLTS    228
P12236       GIRGLYQGFSVSVQGIIIYRAAYFGVYDTAKGMLPDPKNTHIVVSWMIAQTVTAVAGVVS    228
             *: ******.*******:***:*** ***.**:**.**:. :.:*::*** **: :*: *

Q9H0C2       YPFDTVRRRMMMQSGEA--KRQYKGTLDCFVKIYQHEGISSFFRGAFSNVLRGTGGALVL    298
P12235       YPFDTVRRRMMMQSGRKGADIMYTGTVDCWRKIAKDEGAKAFFKGAWSNVLRGMGGAFVL    288
P05141       YPFDTVRRRMMMQSGRKGTDIMYTGTLDCWRKIARDEGGKAFFKGAWSNVLRGMGGAFVL    288
P12236       YPFDTVRRRMMMQSGRKGADIMYTGTVDCWRKIFRDEGGKAFFKGAWSNVLRGMGGAFVL    288
             ***************.   .  *.**:**: ** :.** .:**:**:****** ***:**

Q9H0C2       VLYDKIKEFFHIDIGGR      315
P12235       VLYDEIKKYV-------      298
P05141       VLYDEIKKYT-------      298
P12236       VLYDELKKVI-------      298
             ****::*:
```

selected ANT peptides   excluded ANT peptides

Supplementary Figure 3 (continued)

B

Methionine oxidation of GTDIM(ox)YTGTLDCWR peptide (ANT2)

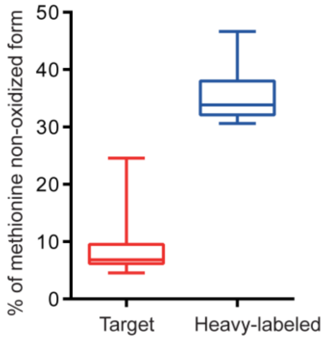

C

Insufficient signal of GIVDCIVR peptide (ANT3)

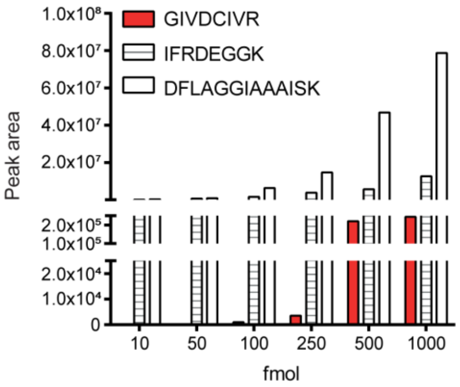

D

RT and FWHM shift of GIVDCIVR peptide (ANT3)

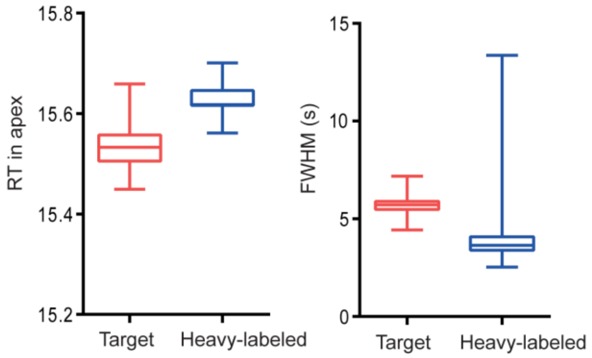

Supplementary Figure 3 (continued)

E

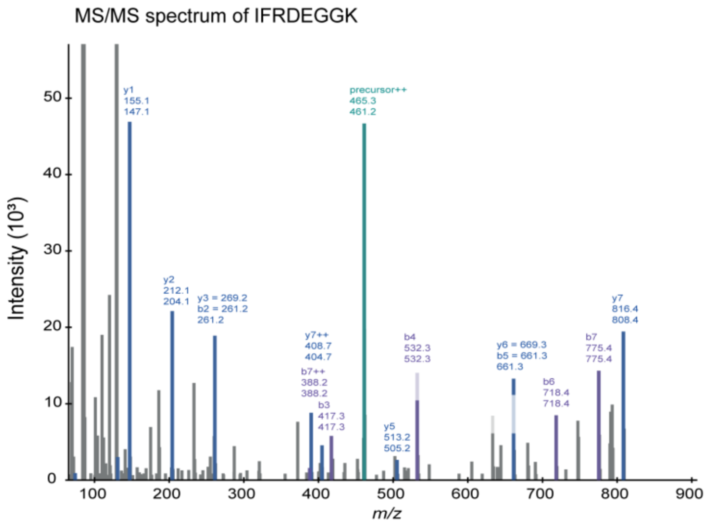

F

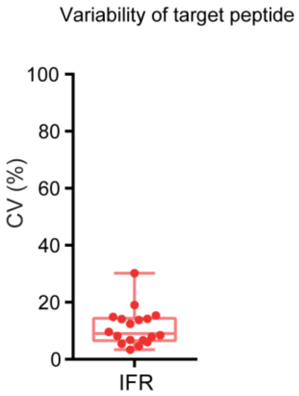

G

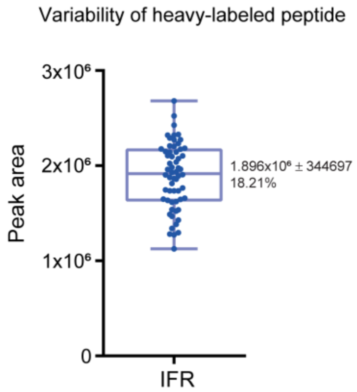

H

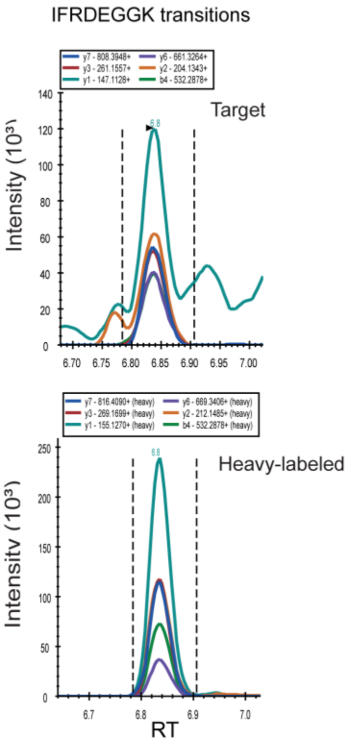

Supplementary Figure 3 (continued)

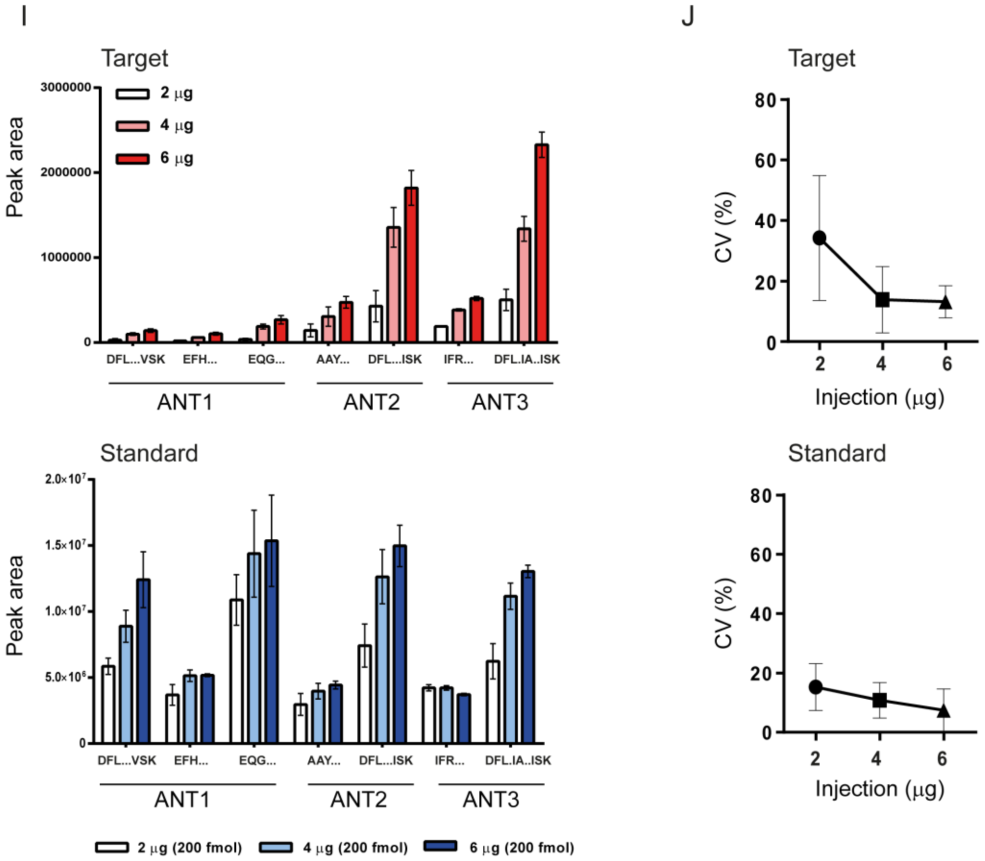

Supplemental Figure 3 (continued)

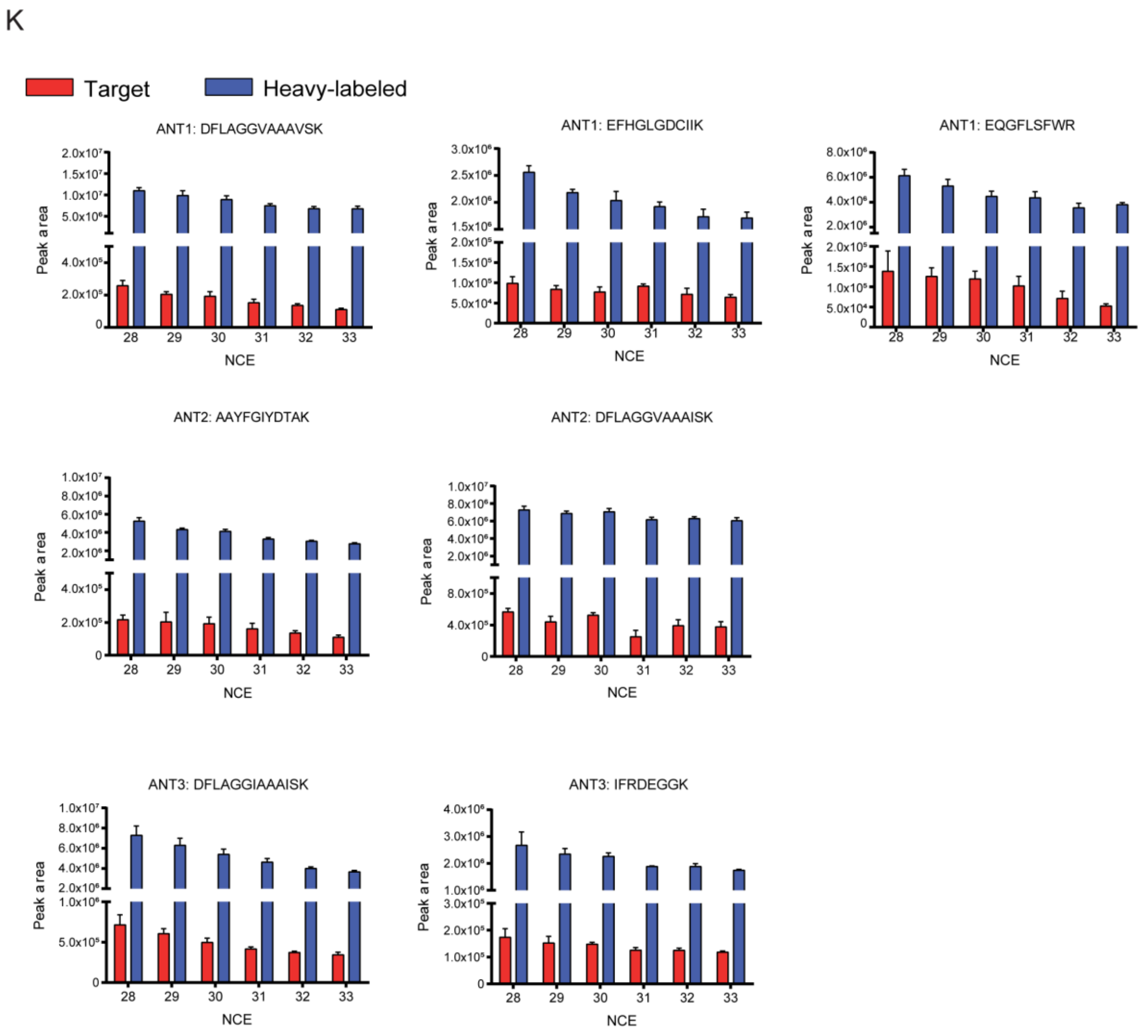

Supplementary Figure 3 (continued)

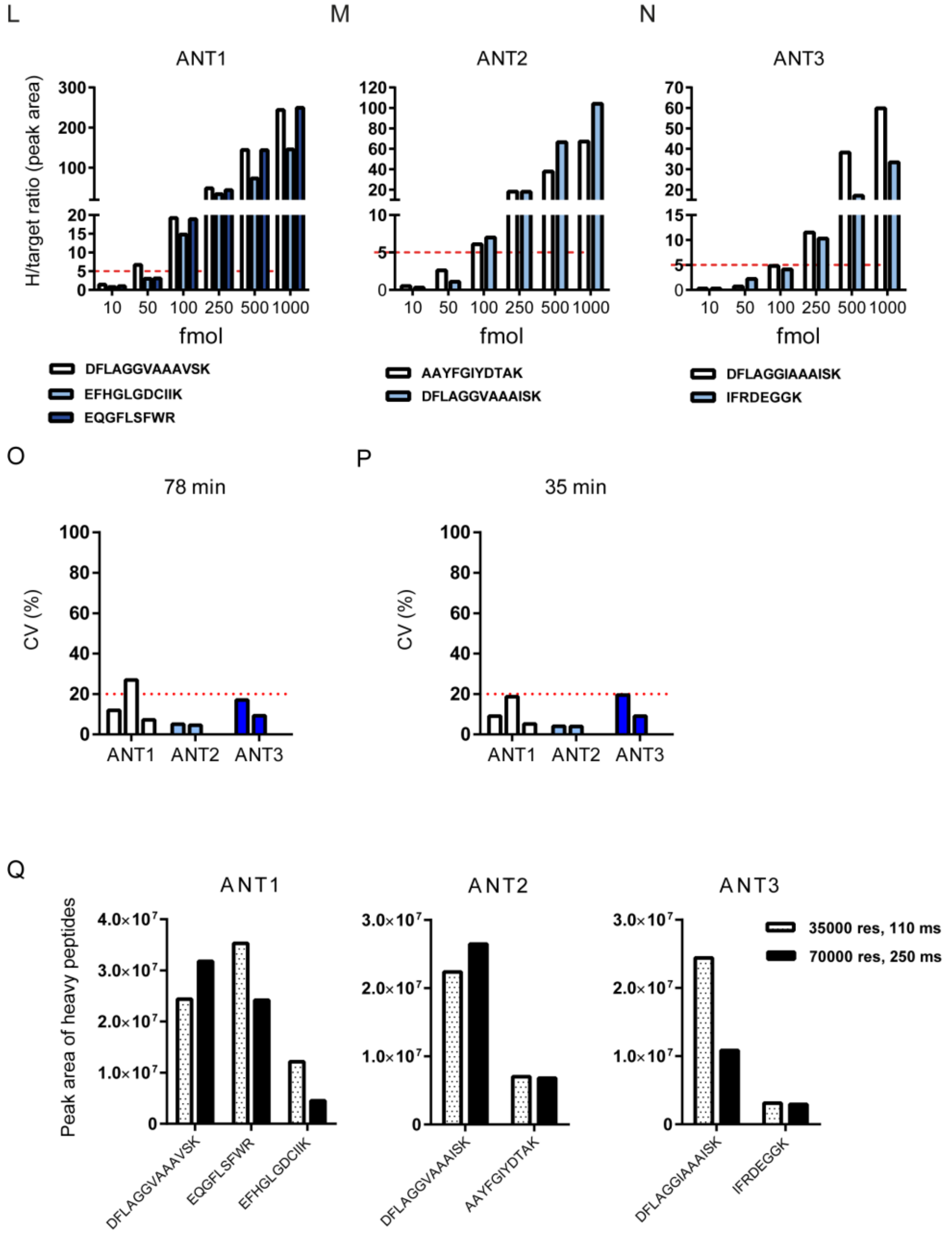

Supplementary Figure 3 (continued)

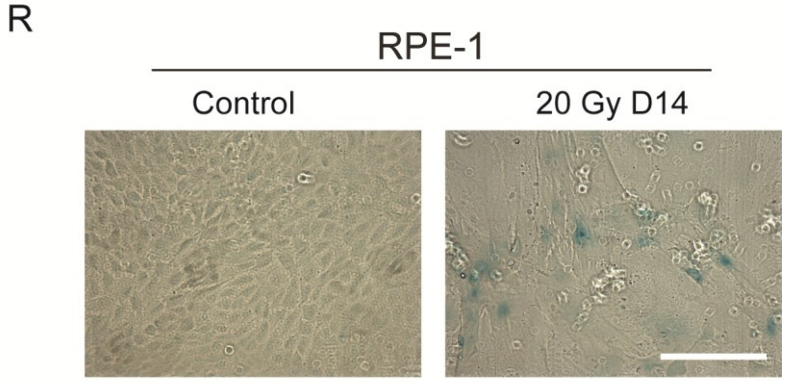

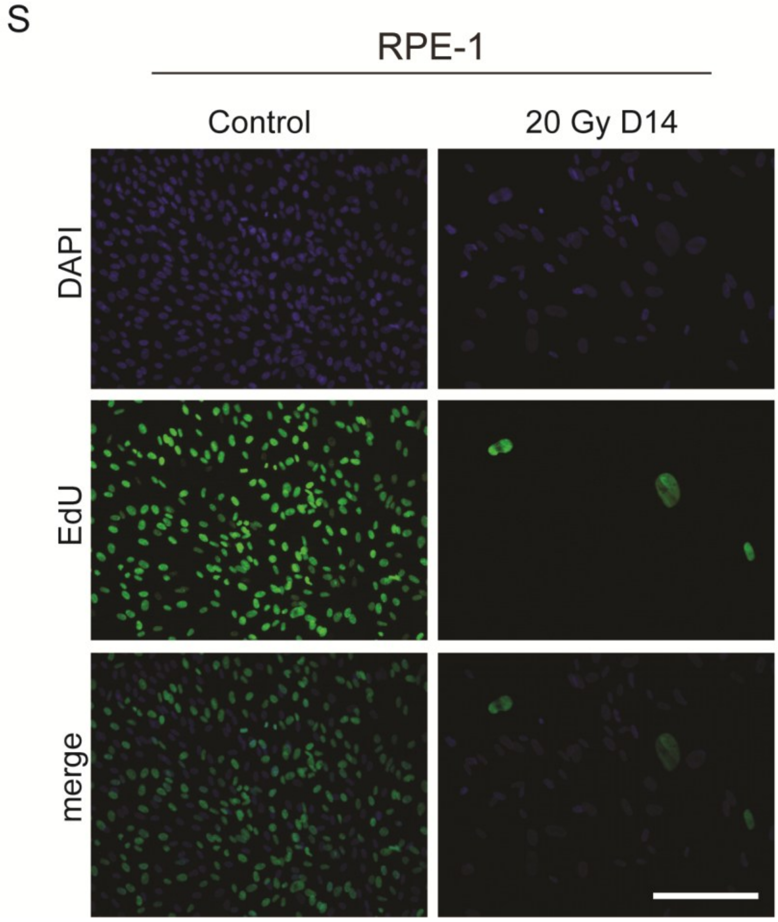

Supplementary Figure 3 (continued)

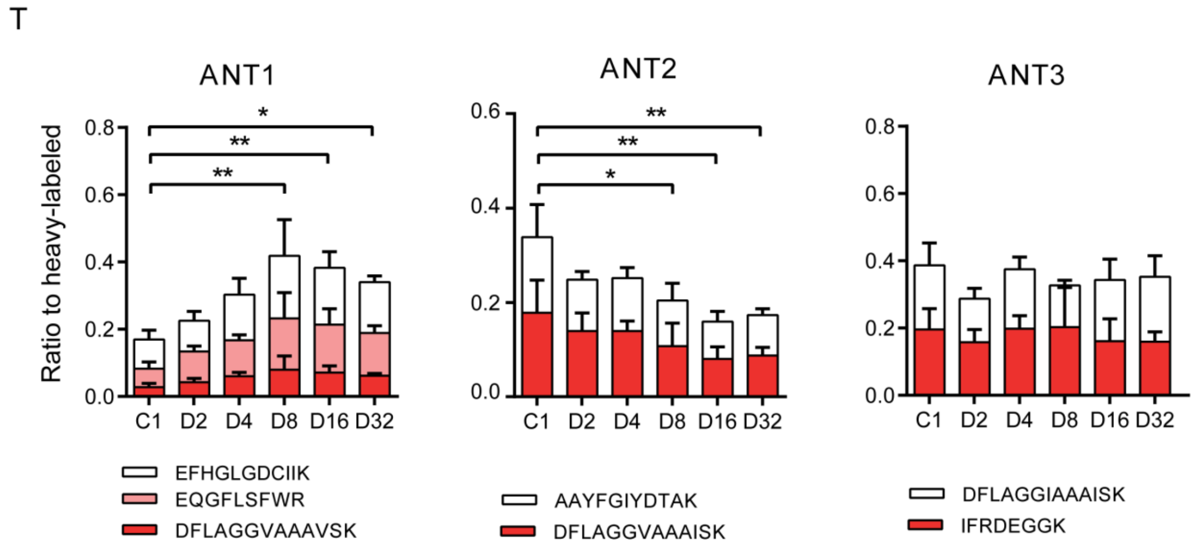

Supplementary Figure 4

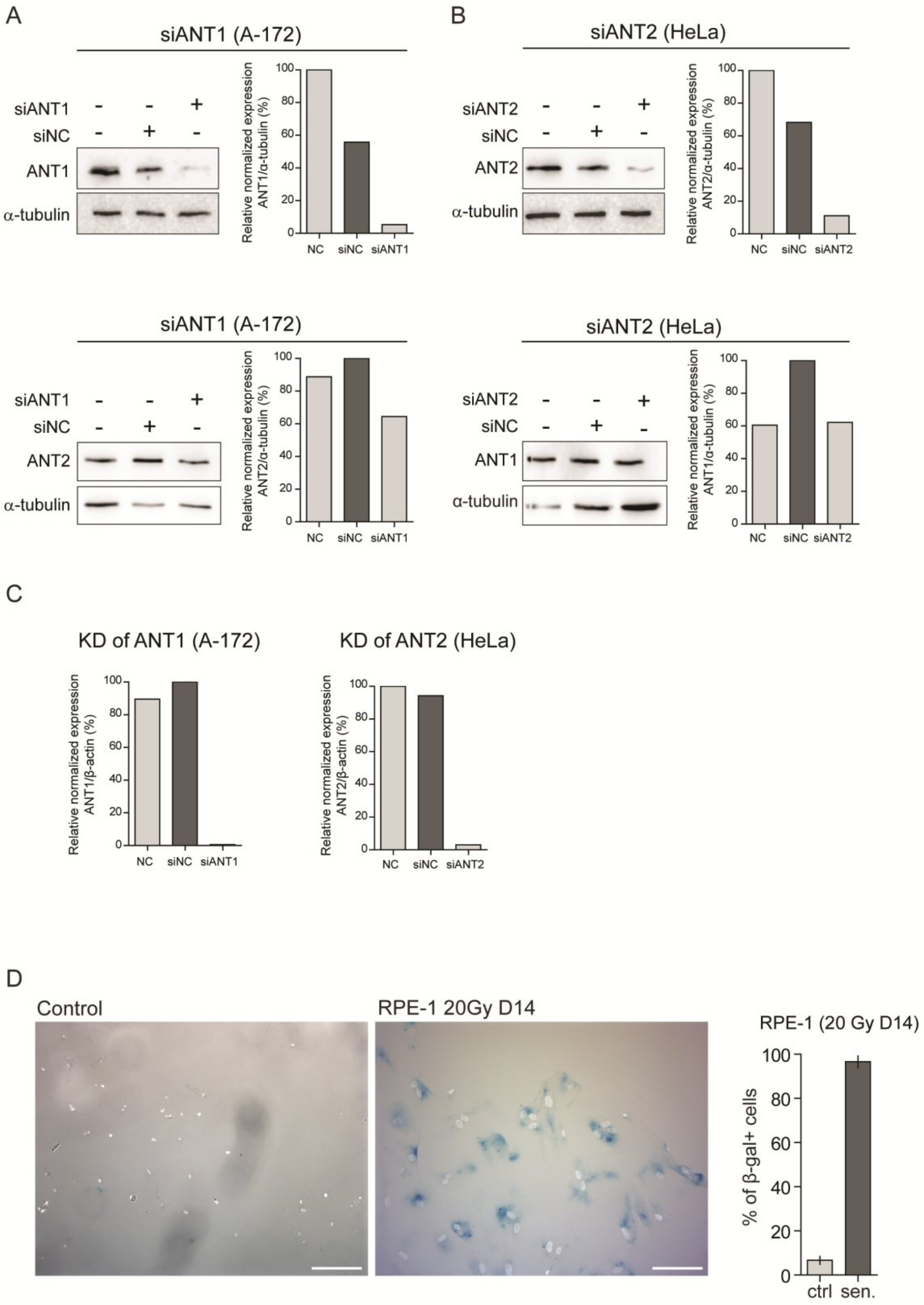

Supplementary Figure 5

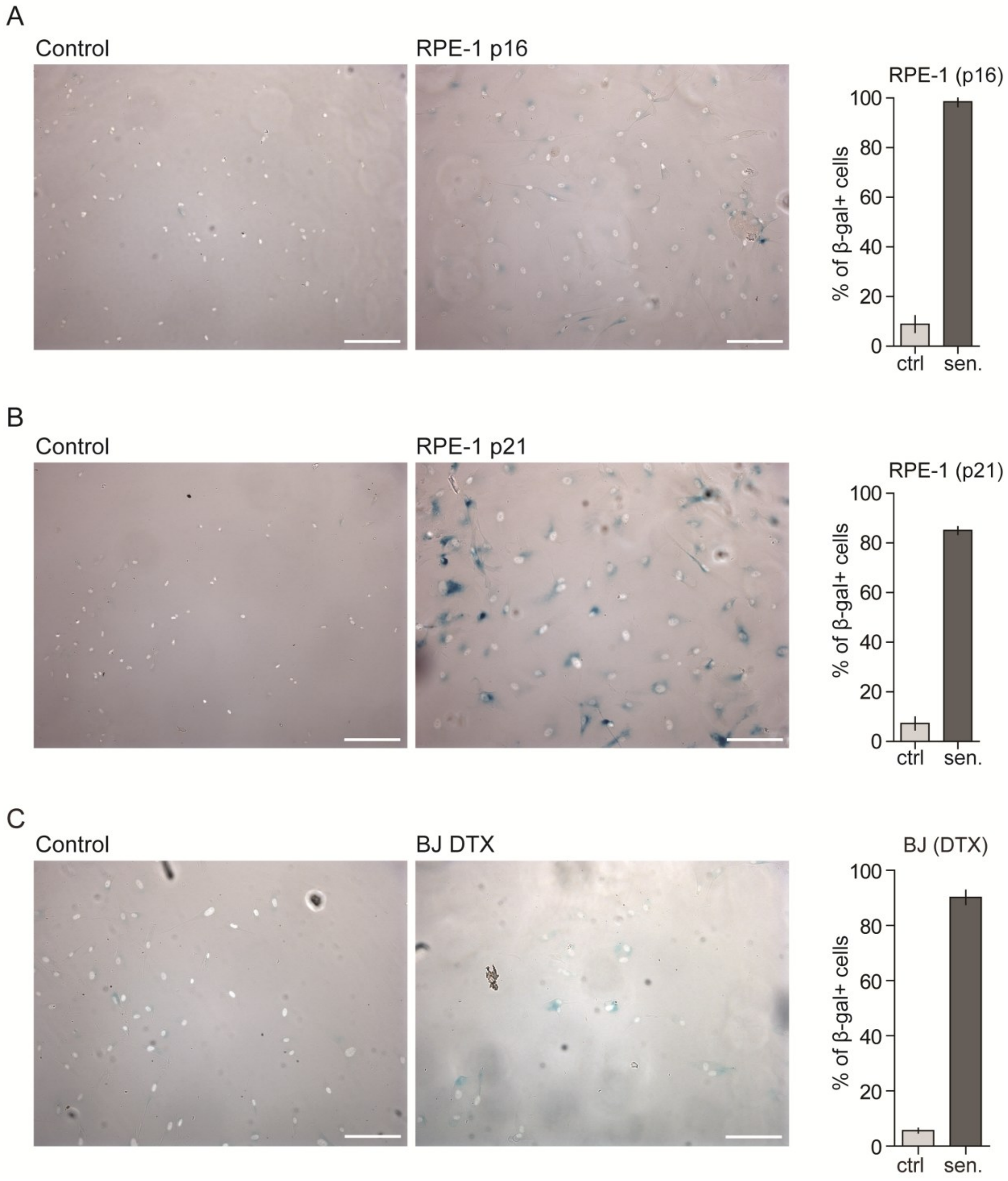

Supplementary Figure 5 (continued)

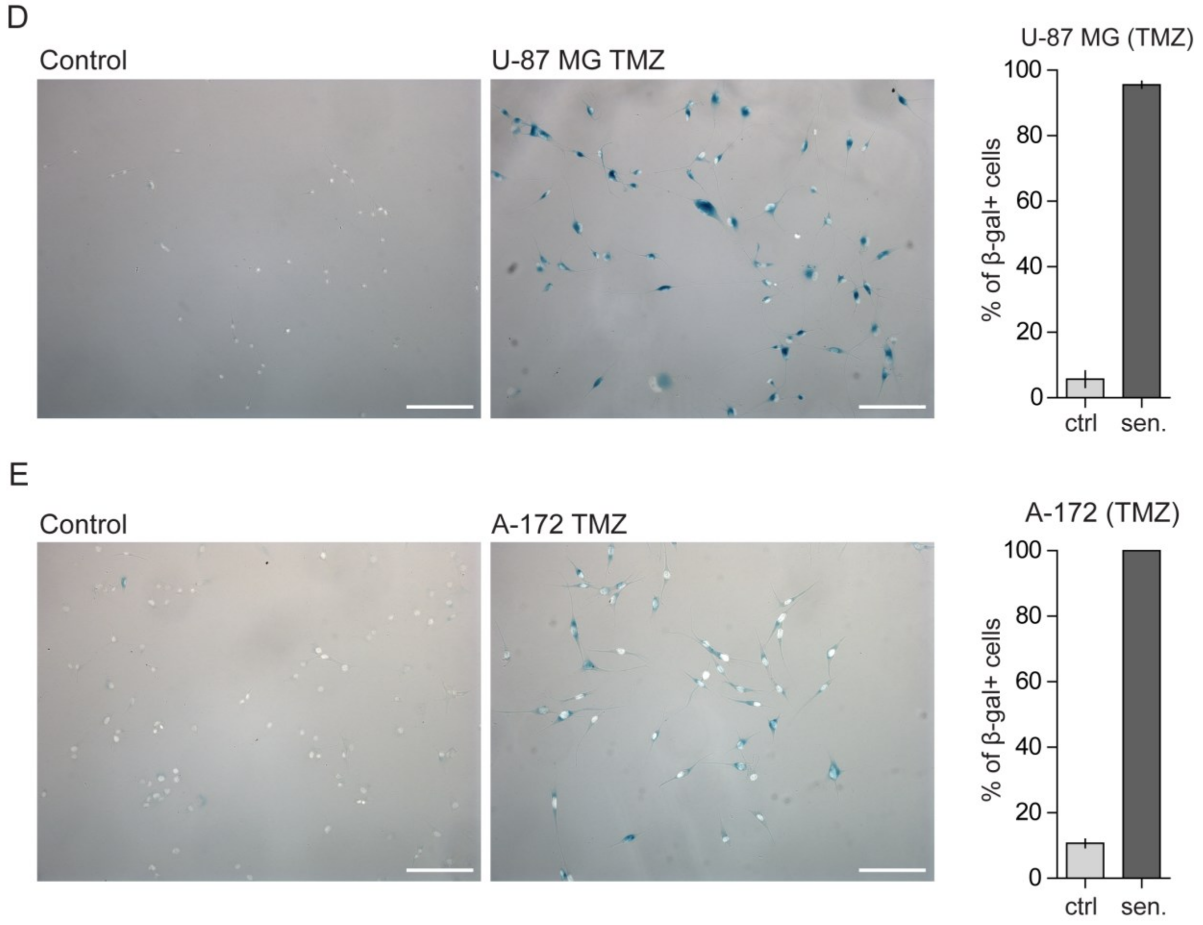

Supplementary Figure 5 (continued)

F

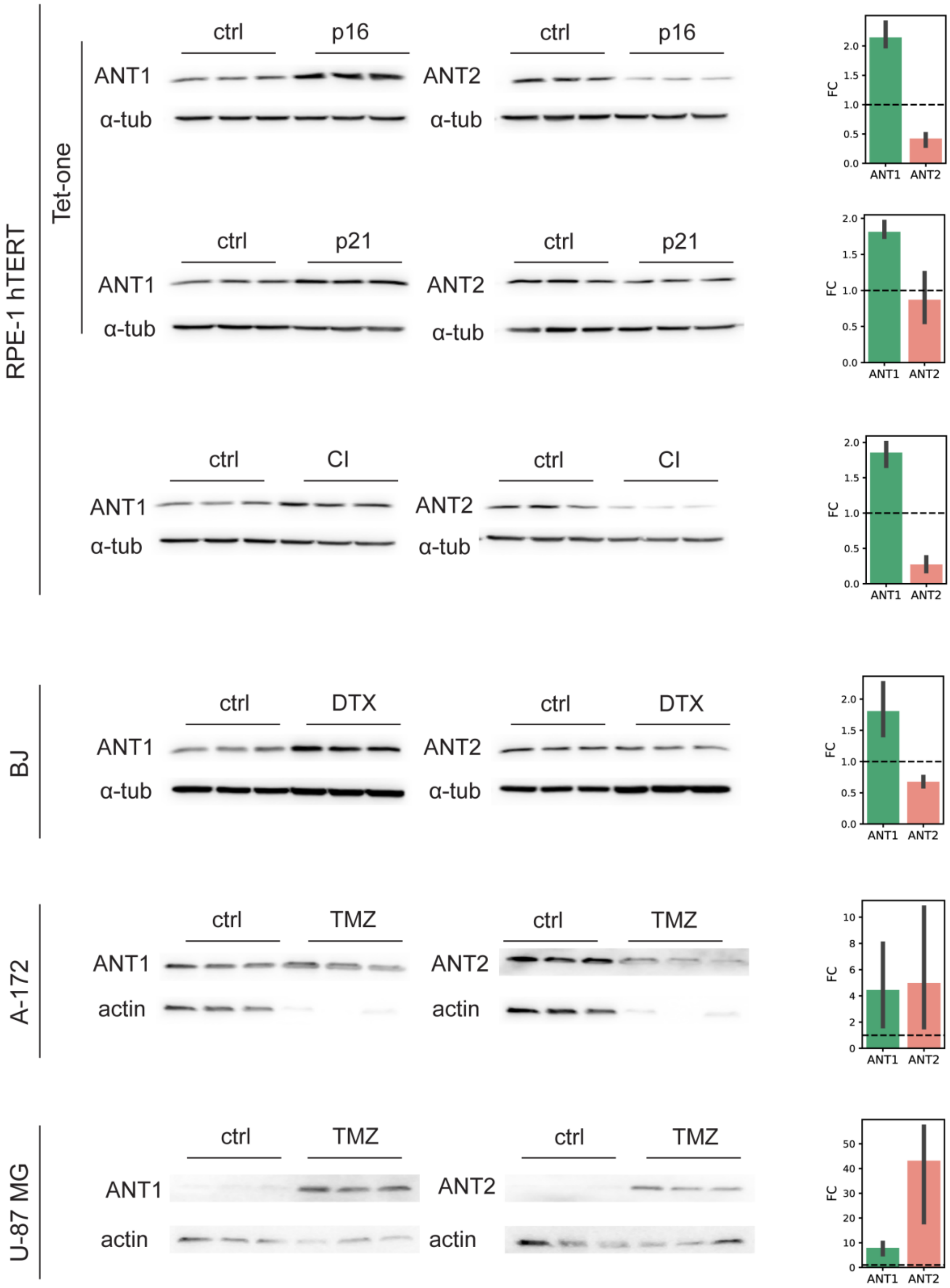

Supplementary Figure 6

A

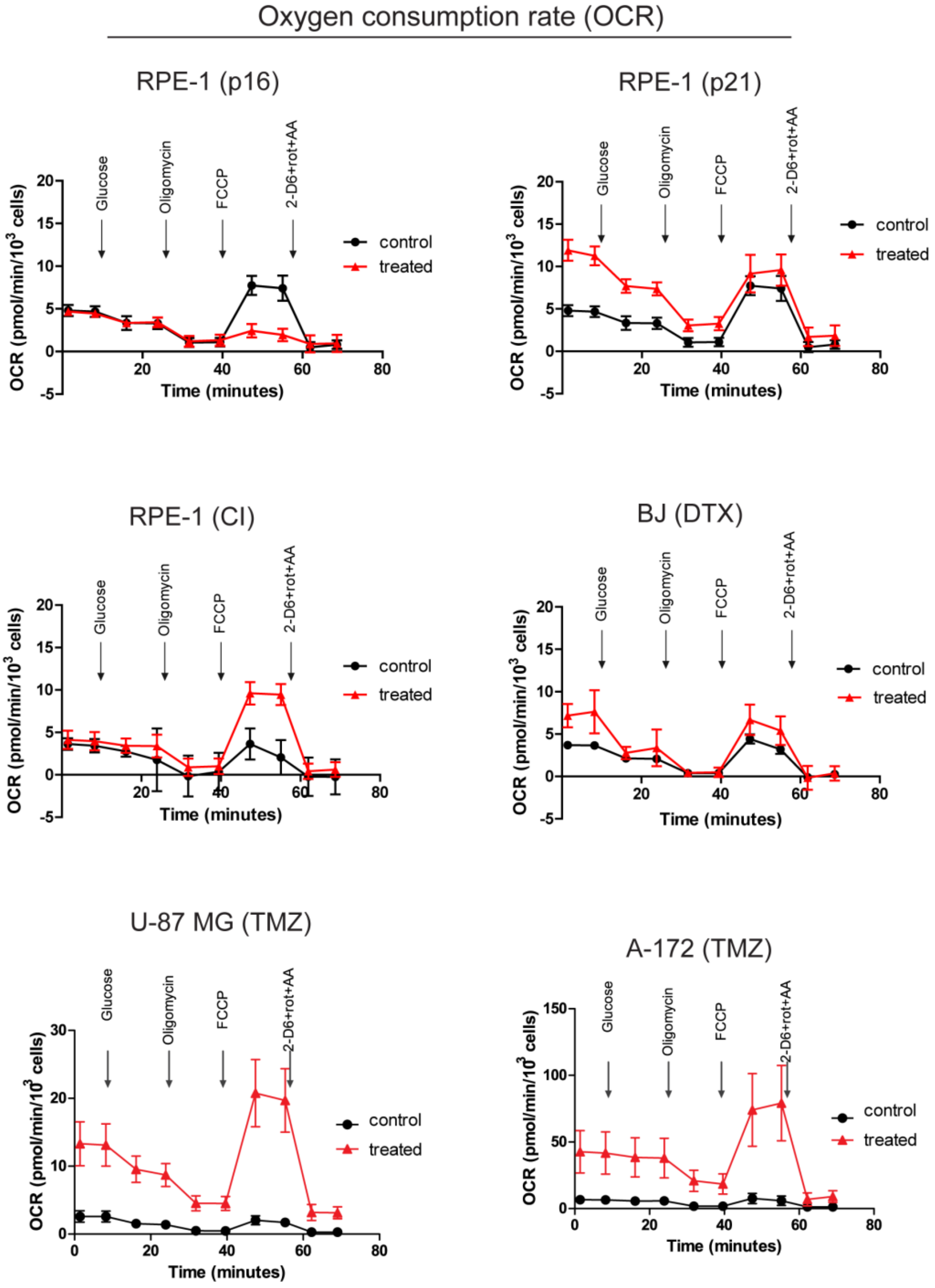

Supplementary Figure 6 (continued)

B

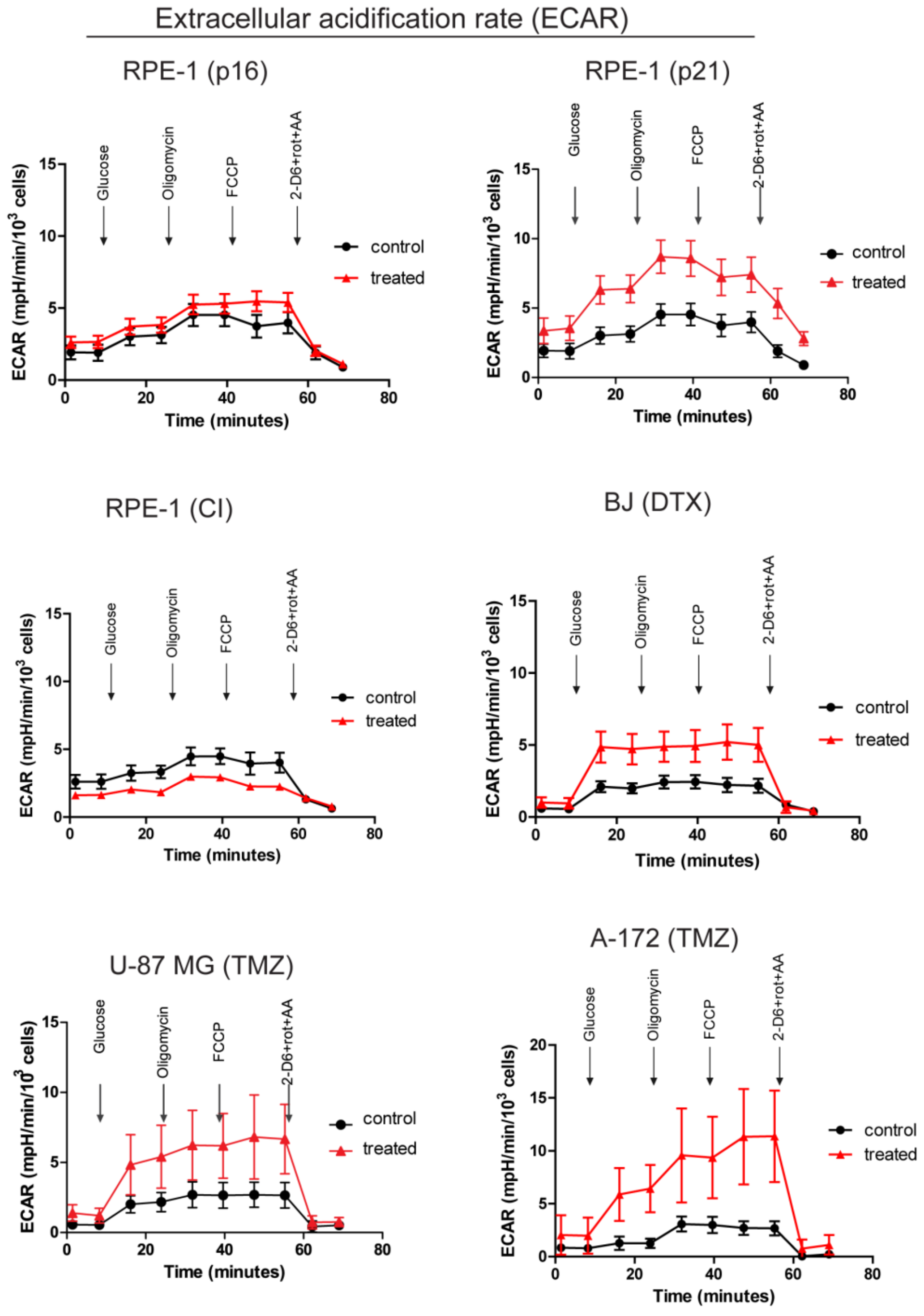

Supplementary Figure 6 (continued)

C

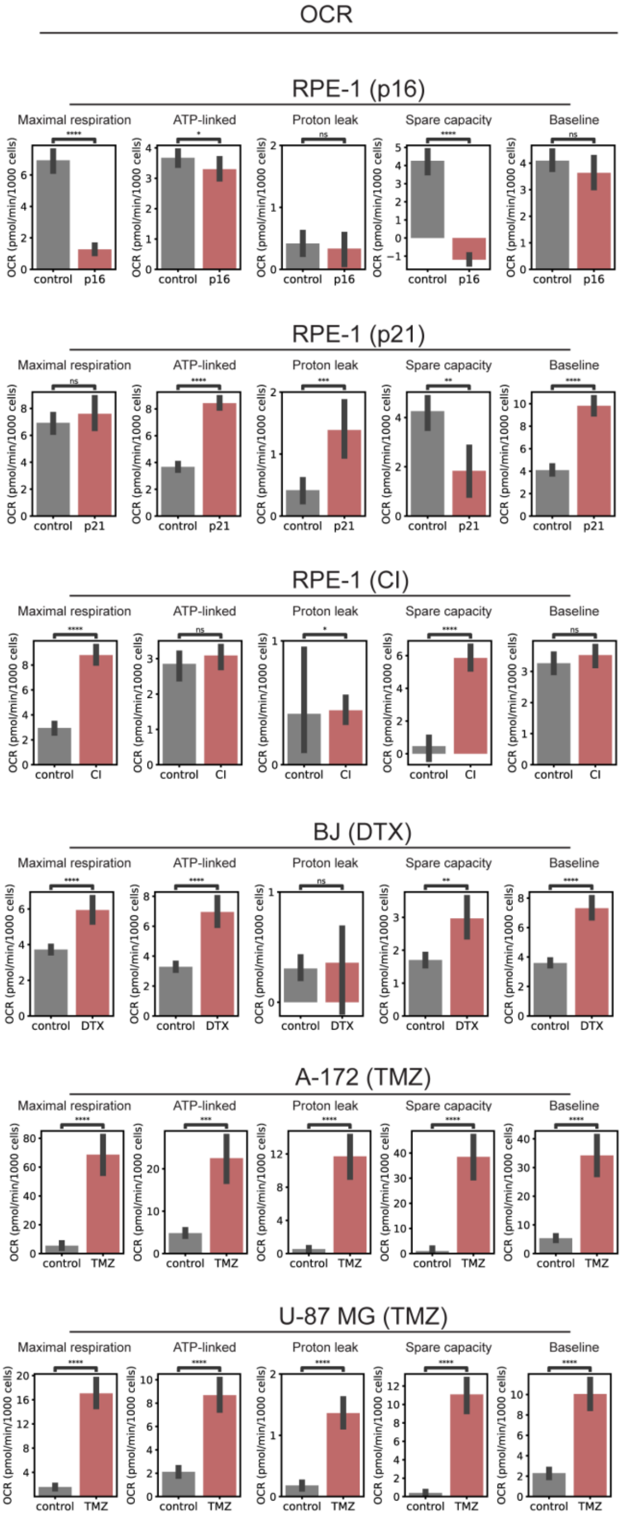

D

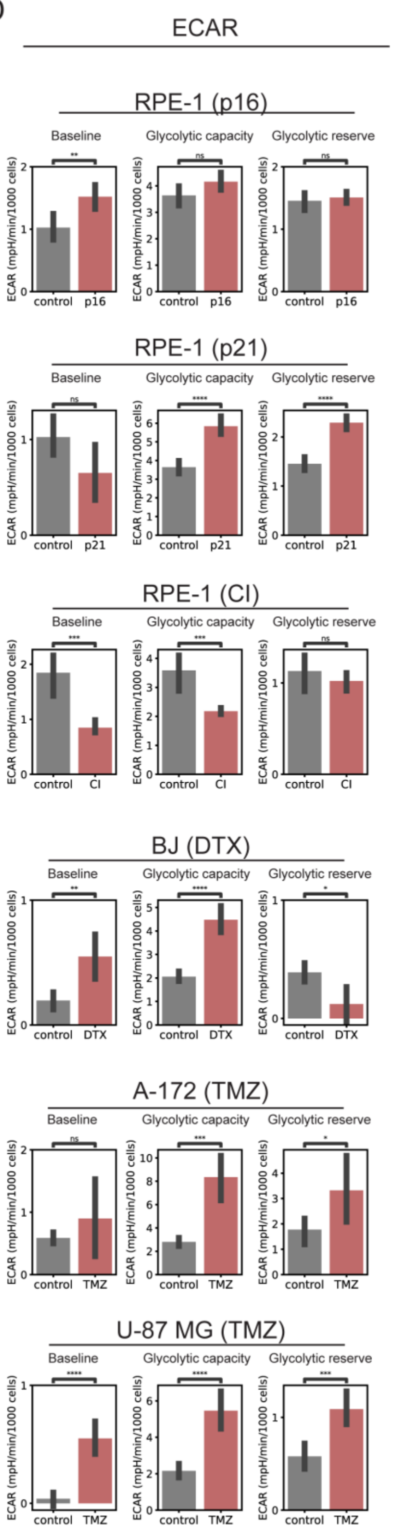

Supplementary Figure 6 (continued)

E

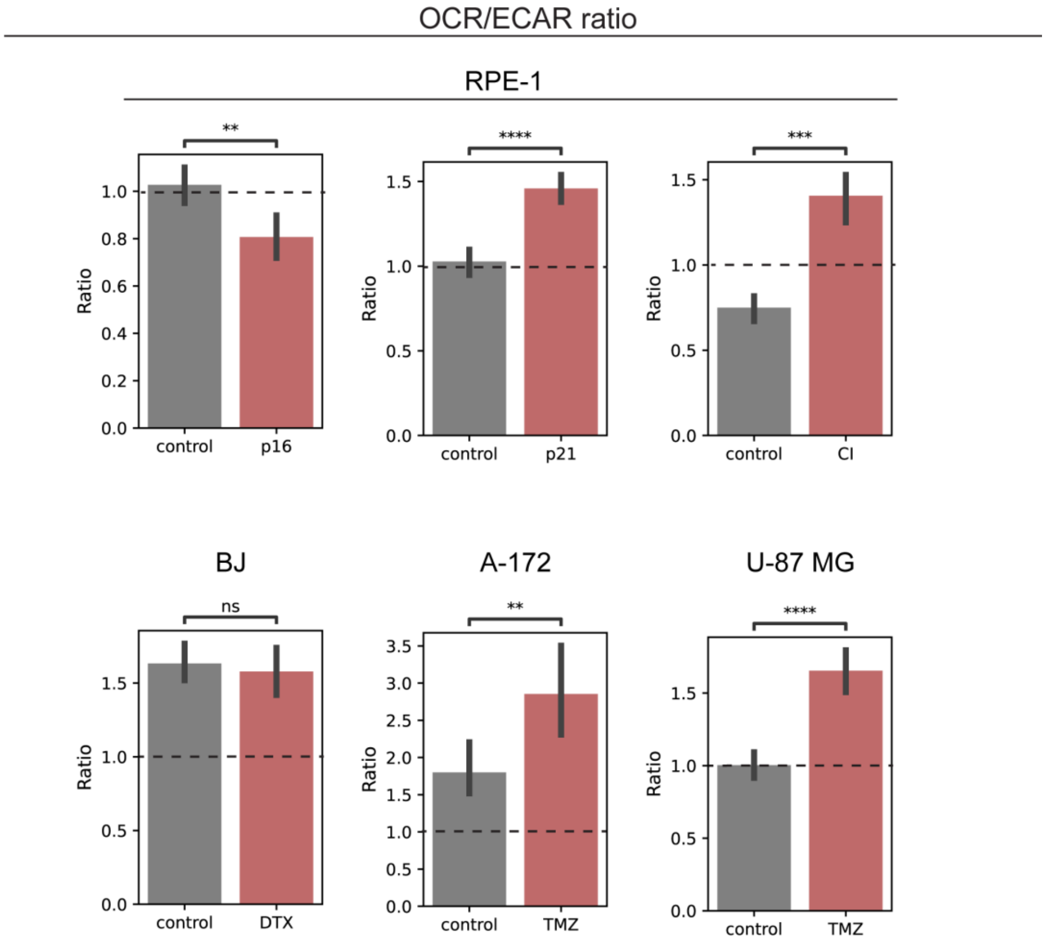

Supplementary Figure 6 (continued)

F

Maximal respiration OCR

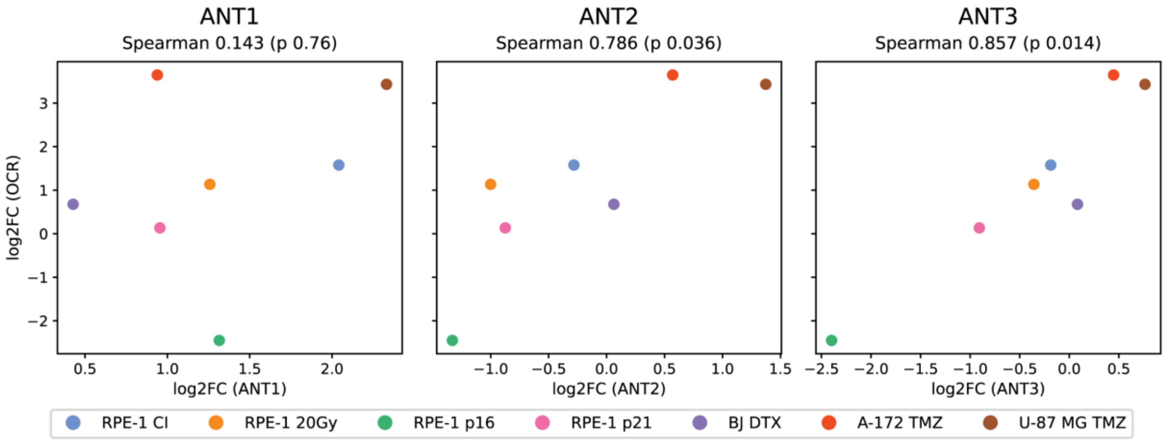

G

Glycolytic capacity ECAR

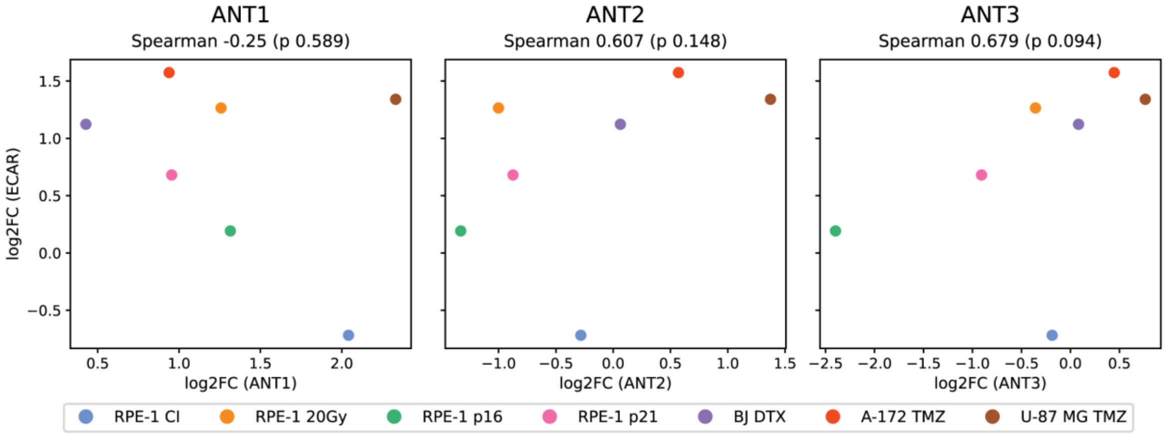

H

Proton leak OCR

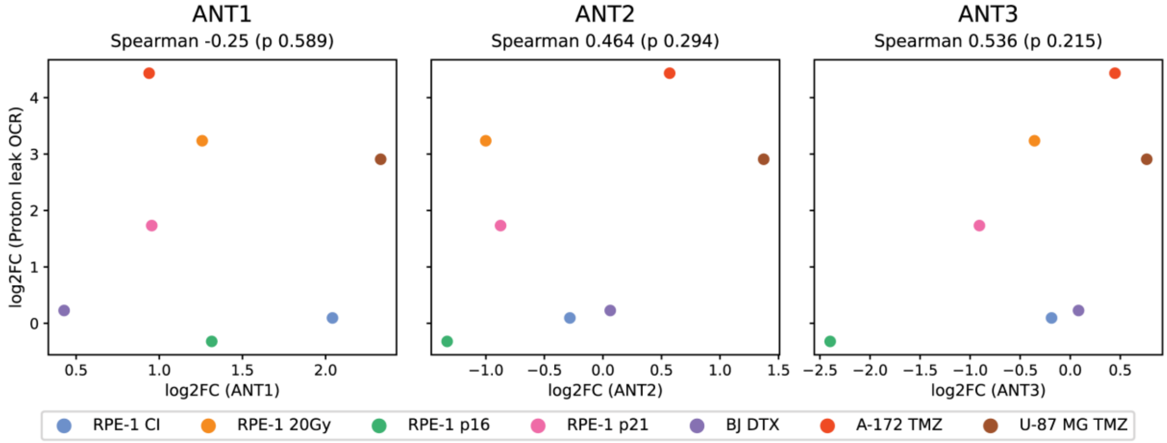

Supplementary figure legends

Supplementary Figure 1: **Changes in ANTs transcripts extracted from six publicly available datasets and their expression in single-cell transcriptomic data of glioblastoma.** (**A**) Changes in ANTs transcripts in pigment naevi, adenomas, liver lesions (HBV, hepatitis B Virus; HCV, hepatitis C Virus), and senescence-induced and senescence-bypassed cells are shown, with their statistical significance level (*p*-value) represented by the area of the individual dots and color-coded by fold change (FC) value. Significant changes (*p*-value less than 0.05) are marked with a black circle. t-SNE visualization of individual glioblastoma cells, color-coded according to (**B**) expressed ANTs, (**C**) cell type, and (**D**) overlapping expression of SLC25A5 and MKI67.

Supplementary Figure 2: **Protein and mRNA correlation in glioblastoma and lung carcinoma**. (**A**) Examples of mRNA and protein correlations in glioblastoma, where Spearman correlation coefficients are in parentheses. (**B**) Spearman correlation matrix for ANT proteins and mRNA in glioblastoma. (**C**) Chart with ANT1, ANT2, ANT3, and TOMM70 (a representative of mitochondrial proteins) protein intensity on the x-axis, all glioblastoma samples, and 10 normal brain tissue on the y-axis, where tumor and normal tissue are distinguished by point shape and individual proteins by color. (**D**) Spearman correlation matrices of ANT proteins with specific mitochondrial proteins in glioblastoma. (**E**) Scatter plots for ANT with transcript level (mRNA) on the x-axis and protein intensity on the y-axis. (**F**) Spearman correlation matrix for ANT proteins and mRNA in lung carcinoma. (**G**) 3D scatter plots for ANTs transcript level and ANTs protein intensity. (**H**) Chart with ANT protein intensity on the x-axis and all lung carcinoma samples on the y-axis with their paired normal adjacent tissues, where normal and tumor tissue are distinguished by color and ANT by point style. (**I**) Comparison of distributions of ANT correlations with all proteins and with mitochondrial proteins. (**J**) Spearman correlation matrices of ANT proteins with specific mitochondrial proteins in lung carcinoma. (**K**) Pearson correlation matrix for ANTs with patient characteristics in glioblastoma samples (Score KP, Karnofsky preoperative score; Score ECOG, Eastern Cooperative Oncology Group Performance Status). Boxplots of ANT protein intensities for different tumor sizes (classified using AJCC Cancer Staging System) in glioblastoma (**L**) and in lung carcinoma (**M**).

Supplementary Figure 3: **Selection of potential ANT peptides and optimization of μLC-PRM method using ionizing radiation-induced senescence in RPE-1 cells**. (**A**) Sequence alignment of ANTs. Multiple sequence alignment was conducted using Clustal Omega. Selected (red square) and excluded ANT (black square) peptides were depicted (Q9H0C2 – ANT4; P12235 – ANT1; P05141 – ANT2; P12236 – ANT3). (**B**) The artificial methionine oxidation of ANT2 peptide (GTDIM(ox)YTGTLDCWR) was estimated based on the peak area of its oxidized form and expressed in percent of the peak area of its methionine non-oxidized form (GTDIMYTGTLDCWR). (C, D) The low signal and stability of ANT3 peptide GIVDCVR during the development of IRIS. Data were collected at each time-point of the IRIS experiment, including control (C1, D2–D32). (**C**) The peak area of heavy-labeled ANT3 peptide GIVDCVR compared to the peak area of other ANT3 standard peptides in the concentration range from 10 to 1000 fmol on the background of 6 μg RPE-1 lysate. (**D**) Retention time

in peak apex (RT in apex) and the full-width half-maximum (FWHM, s) of target and heavy-labeled ANT3 peptide GIVDCVR. (E, F, G, and H) The one missed cleavage in ANT3 peptide IFRDEGGK has been detected. Data was collected at each time-point of the IRIS experiment, including control (C1, D2–D32). (**E**) MS/MS spectrum of IFRDEGGK peptide with efficiently interpreted y and b ions. (**F**) Peak area variability of the target peptide was expressed as a percent of coefficient variability from three technical replicates in each time-point of the IRIS experiment (C1, D2–D32). (**G**) Peak area variability of the heavy-labeled peptide was expressed based on the variance of values achieved in each time-point of IRIS experiment (C1, D2–D32). Mean ± standard deviation and coefficient variation in percent (%) are depicted. (**H**) Transitions for target and heavy-labeled peptide IFRDEGGK extracted in Skyline. The Optimization of appropriate injection of RPE-1 lysate in μLC system is shown in panels I, J, K**.** (**I**) The peak area of target peptides and synthetic isotopically labeled equivalents of ANT1, ANT2, and ANT3 after injections of 2, 4, and 6 µg of RPE-1 lysate. The peak area of each peptide is expressed as a mean of three technical replicates, and standard deviations are depicted as error bars. (**J**) The coefficient variability (CV (%)) was determined from three technical replicates for all target peptides and heavy-labeled standards. Standard deviations were depicted as error bars. An appropriate amount of ANT heavy peptides spiked into RPE-1 lysate to avoid overloading. (**K**) Optimization of normalized collision energy (NCE). The peak areas of target ANT peptides and heavy labeled standards (250 fmol) were extracted and compared upon various values of NCE (28 – 33%). Each value of peak area was expressed as a mean from three technical replicates along with standard deviations depicted as error bars. The isotopically labeled heavy peptides were added to 6 µg of RPE-1 cell lysate in the concentrations of 10, 50, 100, 250, 500, and 1000 fmol, and ratios of heavy peptides (h) to ANT1 (**L**), ANT2 (**M**), and ANT3 (**N**) target peptides (t) were determined. The dashed red line shows the 5-fold level of the heavy-labeled peptides relative to the target peptides. Reduction of μLC-PRM duration. The μLC-PRM robustness was determined as the coefficient of variability (CV (%)) in the case of method duration 78 min (**O**) and 35 min (**P**). The CV (%) was determined based on peak areas of appropriate heavy peptides from three technical replicates. The variability of 20% is depicted as a dashed red line. (**Q**) Peak areas of labeled heavy peptides in 35 min-method with MS resolution of 70,000 or 35,000 and maximum ion filling time 250 ms or 110 ms, respectively, were compared. Validation of senescence induction by (**R**) detection of β-galactosidase activity and (**S**) lack of EdU incorporation (24-hour pulse) in irradiated (14 days; D14) and control (proliferating) RPE-1 cells. (**T**) Contribution of individual ANT peptides to total measured ANT protein levels as shown in Fig. 3B. (R, S) Scale bar, 200 µm.

Supplementary Figure 4: ***Validation of specificity of anti-ANT antibodies and of senescence induction in IRIS RPE-1.*** To assess the specificity of ANT antibodies, RNA interference was utilized to knock down ANT1 and ANT2 transcripts in A-172 and HeLa cells, and expression of ANTs was normalized to the protein expression level of α-tubulin. Three biological replicates were used for each treatment. (**A**) Knock-down of ANT1 in A-172 cells and analysis of ANT1 (top) and ANT2 (bottom) by immunoblotting. (**B**) Knock-down of ANT2 in HeLa cells and analysis of ANT2 (top) and ANT1 (bottom) by immunoblotting. (**C**) Changes in mRNA levels were analyzed using qPCR. For quantification, data

were normalized to the mRNA level of β-actin and compared to the negative control without transfection. (**D**) The development of senescence in IRIS RPE-1 cells (20 Gy; 14 days; D14) was proved by detection of β-galactosidase activity. The nuclei were co-stained by DAPI (white). Scale bars, 200 µm. The quantification of β-gal positive cells is shown on the right.

Supplementary Figure 5: ***Detection of β-galactosidase activity and ANT1 and ANT2 protein levels using immunoblotting in different senescence models.*** Cells were stained for SA-beta-gal and nuclei (DAPI; white). For each senescence type, a representative image of control cells and cells following senescence induction is shown. At least 100 cells for each condition were analyzed to determine the percentage of cells positive for β-galactosidase activity. The tested models are (**A**) p16 CDK-inhibitor overexpression (RPE-1 p16), (**B**) p21 CDK-inhibitor overexpression (RPE-1 p21) in RPE-1 cells, (**C**) docetaxel-induced senescence in normal BJ fibroblasts (BJ DTX), and temozolomide-induced senescence in glioblastoma cell lines (**D**) U-87 MG TMZ, and (**E**) A-172 TMZ. (A – E) Scale bars, 200 µm. (**F**) Changes in ANT1 and ANT2 protein levels analyzed by immunoblotting are shown for different senescence models, as well as RPE-1 cells in contact inhibition (CI). The representative immunoblots from 3 biological replicates used to quantify ANT1 and ANT2 protein levels are shown. The protein levels were normalized to α-tubulin or β-actin, as indicated and the mean of fold change between control and senescence is shown. Three biological replicates were used for each treatment.

Supplementary Figure 6: ***Characteristics of cellular energy metabolism in different senescence models.*** Analysis of glycolysis and OXPHOS via quantification of OCR (**A**) and ECAR (**B**) were processed using Seahorse Analyzers in in RPE-1 overexpressing p16 (RPE-1 p16; three biological/ ten-eleven technical replicates) and p21 (RPE-1 p21; three biological/eleven technical replicates) CDK-inhibitor, in RPE-1 with contact inhibition (RPE-1 CI; three biological/twelve technical replicates), in normal BJ fibroblasts with docetaxel-induced senescence (BJ DTX; three biological/fourteen technical replicates), in glioblastoma cell lines with temozolomide-induced senescence (A-172 TMZ; three biological/twelve-fourteen technical replicates and U-87 MG TMZ; three biological/fifteen technical replicates). From obtained OCR and ECAR data, the (**C**) OCR characteristics, (**D**) ECAR characteristics, and (**E**) OCR/ECAR ratio (ATP-linked OCR divided by glycolytic ECAR) were computed for all tested senescence models. From obtained protein levels of ANT isoforms and OCR and ECAR data, the correlations between $\log_2$ fold changes of individual ANT proteins and (**F**) maximal respiration OCR $\log_2$ fold change, (**G**) glycolytic capacity ECAR $\log_2$ fold change and (**H**) proton leak OCR $\log_2$ fold change were calculated. Spearman's correlation coefficient (Spearman) and p-value are shown.

Supplementary tables

Supplementary Table 1. Unique peptides selected for the targeted quantification of ANT1, ANT2, and ANT3 isoforms by μLC-PRM.

	Peptide sequence
ANT1 *(SLC25A4)*	DFLAGGVAAAVSK
	EFHGLGDCIIK
	EQGFLSFWR
ANT2 (*SLC25A5*)	AAYFGIYDTAK
	DFLAGGVAAAISK
ANT3 (*SLC25A6*)	DFLAGGIAAAISK
	IFRDEGGK

Supplementary Table 2. Unique peptides rejected from the targeted quantification of ANT1, ANT2, and ANT3 isoforms by μLC-PRM.

	Peptide sequence	
ANT1 *(SLC25A4)*	GDHAWSFLK	N-terminal signal peptides prone to acetylation
ANT2 (*SLC25A5*)	TDAAVSFAK	N-terminal signal peptides prone to acetylation
	GTDIMYTGTLDCWR	artificial oxidation of methionine
ANT3 (*SLC25A6*)	TEQAISFAK	N-terminal signal peptides prone to acetylation
	GIVDCIVR	low signal even at a concentration of 1,000 fmol; shift in RT

Supplementary Table 3. Forward and reverse primers used for quantification of ANTs transcripts. Three different ANT1 primers were used for the determination of the ANT1 mRNA level.

		Forward	Reverse	References
ANT1 - a (exon 2-3)	ANT1	GCTGCCTACTTCGGAGTCTATG	TGCGACTGCCGTCACACTCTG	
ANT1 - b (exon 1-2)		ATCACGCTTGGAGCTTCCTAA	TGCTTCTCAGCACTGATCTGT	[1]
ANT1 - c (exon 2-3)		CAAGGGGATGCTGCCTGACC	GGACTGCATCATCATTCTACG	[2]
ANT2 (exon 2-3)	ANT2	GCCGCCTACTTCGGTATCTATG	CAGCAGTGACAGTCTGTGCGAT	[3]
ANT3 (exon 2-3)	ANT3	GGTGAAGATCACCAAGTCCGAC	ACCACGATGTGCGTGTTCTTGG	
	ACTB	CCAACCGCGAGAAGATGA	CCAGAGGCGTACAGGGATAG	

Supplementary references

1. Doczi J, Torocsik B, Echaniz-Laguna A, Mousson de Camaret B, Starkov A, Starkova N, Gal A, Molnar MJ, Kawamata H, Manfredi G, Adam-Vizi V & Chinopoulos C (2016) Alterations in voltage-sensing of the mitochondrial permeability transition pore in ANT1-deficient cells. Scientific reports 6, 26700, doi: 10.1038/srep26700.
2. Vial J, Huchede P, Fagault S, Basset F, Rossi M, Geoffray J, Soldati H, Bisaccia J, Elsensohn MH, Creveaux M, Neves D, Blay JY, Fauvelle F, Bouquet F, Streichenberger N, Corradini N, Bergeron C, Maucort-Boulch D, Castets P, Carre M, Weber K & Castets M (2020) Low expression of ANT1 confers oncogenic properties to rhabdomyosarcoma tumor cells by modulating metabolism and death pathways. Cell death discovery 6, 64, doi: 10.1038/s41420-020-00302-1.
3. Mrazkova B, Dzijak R, Imrichova T, Kyjacova L, Barath P, Dzubak P, Holub D, Hajduch M, Nahacka Z, Andera L, Holicek P, Vasicova P, Sapega O, Bartek J & Hodny Z (2018) Induction, regulation and roles of neural adhesion molecule L1CAM in cellular senescence. Aging (Albany NY) 10, 434-462, doi: 10.18632/aging.101404.
